# A Model of Yeast Cell-Cycle Regulation Based on a Standard Component Modeling Strategy for Protein Regulatory Networks

**DOI:** 10.1371/journal.pone.0153738

**Published:** 2016-05-17

**Authors:** Teeraphan Laomettachit, Katherine C. Chen, William T. Baumann, John J. Tyson

**Affiliations:** 1 Genetics, Bioinformatics, and Computational Biology Program, Virginia Polytechnic Institute and State University, Blacksburg, Virginia, United States of America; 2 Department of Biological Sciences, Virginia Polytechnic Institute and State University, Blacksburg, Virginia, United States of America; 3 Department of Electrical and Computer Engineering, Virginia Polytechnic Institute and State University, Blacksburg, Virginia, United States of America; Universitat Pompeu Fabra, SPAIN

## Abstract

To understand the molecular mechanisms that regulate cell cycle progression in eukaryotes, a variety of mathematical modeling approaches have been employed, ranging from Boolean networks and differential equations to stochastic simulations. Each approach has its own characteristic strengths and weaknesses. In this paper, we propose a “standard component” modeling strategy that combines advantageous features of Boolean networks, differential equations and stochastic simulations in a framework that acknowledges the typical sorts of reactions found in protein regulatory networks. Applying this strategy to a comprehensive mechanism of the budding yeast cell cycle, we illustrate the potential value of standard component modeling. The deterministic version of our model reproduces the phenotypic properties of wild-type cells and of 125 mutant strains. The stochastic version of our model reproduces the cell-to-cell variability of wild-type cells and the partial viability of the *CLB2*-*db*Δ *clb5*Δ mutant strain. Our simulations show that mathematical modeling with “standard components” can capture in quantitative detail many essential properties of cell cycle control in budding yeast.

## Introduction

The physiology of living cells is controlled by complex networks of interacting genes, proteins and metabolites [1]. These networks are often simplified by focusing on one level or another. A metabolic network treats genes and proteins as fixed parameters. A gene regulatory network focuses on how one gene controls another, skipping over the proteins that implement these control signals. A protein regulatory network, on the other hand, describes the production, degradation and post-translational modifications of proteins, without explicit reference to the nucleic acids that underpin protein synthesis. Such simplifications are entirely appropriate in certain circumstances, and the right way to model such networks also depends on the context. For instance, genetic control systems are often described by Boolean switching networks, where simple logical rules (Boolean functions) are used to describe the interactions among genes and to determine how the system updates its discrete state variables from one time-point to the next. Boolean models require no rate constants and are primarily used to capture the qualitative behavior of gene regulatory networks [2–4].

To describe the dynamical behavior of protein regulatory networks (PRNs), computational biologists have access to a range of mathematical modeling approaches, from discrete Boolean models to continuous models based on nonlinear ordinary differential equations (ODEs), to stochastic simulations with Gillespie-type models. ODE models are a common choice, because they track the evolution of a PRN continuously over time and their simulations can be compared in exquisite detail to experimental observations [5, 6]. The ODE approach, however, requires that the biochemical rate constants characterizing the reactions in the network be carefully estimated from experimental data. Parameter estimation for realistic models is a difficult task [7].

There have been some notable efforts to combine the advantageous features of ODE and Boolean models. Years ago Glass & Kauffman [8] used piecewise-linear differential equations to track continuous changes of protein concentrations (given by ODEs) in response to discrete changes in gene expression (given by a Boolean network). This approach was used elegantly by Uri Alon in his textbook on systems biology [9] and by a number of other research groups [10–12].

To incorporate molecular noise into network simulations, modelers have recourse to Gillespie's stochastic simulation algorithm (SSA) [13]—which simulates every reaction event and is statistically correct but comes at a high computational price—or less computationally expensive methods such as the chemical Langevin equation (CLE) [14] or stochastic Petri nets [15]. It is possible to include stochastic effects in Boolean models as well [16, 17].

Once a modeler adopts a particular quantitative approach, he or she faces the problem of choosing rate laws to represent the biochemical reactions in the network. Accurate stochastic modeling by SSA or CLE demands (in principle) that the network be described by elementary biochemical reactions with mass-action kinetics. ODE models are more flexible, and phenomenological rate laws (such as Michaelis-Menten kinetics, zero-order ultrasensitivity, and Hill functions) are often used. These phenomenological rate laws rely on some simplifications and assumptions that are, unfortunately, not always true in PRNs [18, 19].

To deal with the issues just described, we propose to model PRNs in terms of three classes of proteins, depending on the time scales of the reactions that govern their evolution. These three classes represent distinctly different “building blocks” of a network and are described by different types of mathematical equations. By connecting these building blocks in standard ways, we can construct a detailed model of a complex reaction network in a controlled fashion, much like a LEGO^®^ toy. We refer to the output of this approach as a **standard component model** (SCM). Our approach combines many advantageous features of continuous, discrete and stochastic approaches, while organizing the model in a simple and logical format.

The goal of this paper is to demonstrate that the SCM approach can be applied effectively to model a complex protein regulatory network, namely the network regulating cyclin-dependent kinase activities during the cell division cycle of budding yeast. We build the model in two steps. First, we build a simple SCM of the Start transition in the budding yeast cell cycle, to illustrate the general principles of deterministic and stochastic modeling by the standard-component method. Then we build a more complex SCM of the full cell division cycle of budding yeast, and test its accuracy by detailed comparisons to experimental data. Our presentation follows this outline:

1. Deterministic modeling by the standard-component method.
2. Molecular cell biology of the Start transition in budding yeast.
3. A “multisite phosphorylation” (MultiP) model of the Start transition.
4. A deterministic SCM of the Start Transition
5. A stochastic version of the SCM.
6. Comparison of the SCM and MultiP models of the Start transition.
7. An SCM of the full cell-cycle control system in budding yeast.
8. Deterministic simulations of the full SCM and comparison to the phenotypes of wild-type and mutant cells.
9. A stochastic version of the full SCM.
10. Comparison of stochastic simulations to the cell-to-cell variations exhibited by wild-type and mutant yeast cells.

## Results

### 1. Deterministic SCM

Proteins regulate one another by controlling their abundances through rates of synthesis and degradation and their activities through post-translational modification (e.g., phosphorylation and dephosphorylation), and by associating into multisubunit complexes. These three classes of reactions often proceed on different time scales. Synthesis and degradation cause rather slow changes in the total amount of a protein (time scale > 10 min). Phosphorylation and dephosphorylation cause faster changes in protein activity (time scale = 1–10 min). Rapid association and dissociation of protein complexes bring the complexes and subunits into equilibrium on a short time scale (1 min or less). In cases where this separation of time scales is known or suspected to be the case, we can partition the components of a PRN into three classes:

Class-1 variables track the total amounts of proteins, which evolve rather slowly in time due to synthesis and degradation.
Class-2 variables track the activities of proteins, which change faster due to covalent modifications.
Class-3 variables track protein complexes, which turn over rapidly by association and dissociation of subunits.

In principle, variables of these three classes can represent different forms of the same protein. For example, we can use a class-1 variable to represent the total amount of a protein, while using a class-2 variable to represent the fraction of the total protein that is phosphorylated, and a class-3 variable to represent the fraction of the total protein that is bound to a stoichiometric inhibitor.

In our formalism, class-1 variables (X_*i*_) are governed by pseudo-linear differential equations for protein synthesis and degradation

$$\frac{\text{d}X_{i}}{\text{d}t}=A_{i}-B_{i}\cdot X_{i}$$ (1)

The ODE is linear in *X*_*i*_, the number of molecules of species X_*i*_, but the rates of synthesis and degradation are functions of variables *Y*_*j*_ that may belong to any of the three classes. It is often possible to use linear functions for *A*_*i*_ and *B*_*i*_,

$$A_{i}=\alpha_{i0}+\underset{j\in J}{\sum }\alpha_{ij}\cdot Y_{j}$$ (2)

$$B_{i}=\beta_{i0}+\underset{j\in J}{\sum }\beta_{ij}\cdot Y_{j}$$ (3)

where *α*_*i*0_ and *β*_*i*0_ are basal rates of synthesis and degradation, and *α*_*ij*_ ·*Y*_*j*_ and *β*_*ij*_ ·*Y*_*j*_ are rates regulated by transcription factors and proteolytic enzymes, respectively. (In this case, the biochemical rate parameters *α*_*i*0_, *β*_*i*0_, *α*_*ij*_, and *β*_*ij*_ are all positive constants.) In other cases—especially for transcription factors that inhibit gene expression—nonlinear functions for *A*_*i*_ and *B*_*i*_ may be required.

Class-2 variables are governed by nonlinear ODEs of the form

$$\frac{\text{d}Y_{j}}{\text{d}t}=\gamma_{j}(Y_{j,\text{T}}\cdot H(\sigma_{j}\cdot W_{j})-Y_{j}),$$ (4)

where *Y*_*j*_ represents the activity of protein Y_*j*_ (e.g., the phosphorylated or the active form of Y_*j*_), *Y*_*j*_,_T_ is the total number of molecules of protein Y_*j*_, *γ*_*j*_ determines the time scale of the reaction, and *H*(*x*) = 1/(1 + *e*^−*x*^) is the sigmoidal function illustrated in S1 Fig. (*H* is a hyperbolic tangent function shifted along the y-axis. In population biology it is known as the “logistic” function. We refer to *H* as the “soft-Heaviside” function, because we use it to replace the step-like Heaviside function used in the piecewise-linear models of Glass, Kauffman and others.) In the soft-Heaviside function, *W*_*j*_ describes the net influence of all components in the network on the component Y_*j*_:

$$W_{j}=\pm \omega_{j0}+\underset{k\in K}{\sum }\omega_{jk}\cdot Y_{k}-\underset{l\in L}{\sum }\omega_{jl}\cdot Y_{l}.$$ (5)

In Eq 5, *ω*_*jk*_ and *ω*_*jl*_ are weights (always positive values) that describe the influences of variables *Y*_*k*_ and *Y*_*l*_ on the variable *Y*_*j*_. **K** represents all variables that have positive influences on the variable *Y*_*j*_, and **L** represents all variables that have negative influences on the variable *Y*_*j*_. *Y*_*k*_ and *Y*_*l*_ can be variables of any of the three classes of species. The background influence, *ω*_*j*0_, which can be preceded by either the positive or negative sign, determines the value of the soft-Heaviside function when protein Y_*j*_ is receiving no inputs from the other proteins in the network. The parameter *σ*_*j*_ controls the steepness of the soft-Heaviside function; see S1 Fig. In principle, the value of *σ*_*j*_ could be absorbed into the values of the *ω*’s, but we prefer to treat *σ*_*j*_ as a separate parameter and to think of the *ω*’s as relative interaction strengths. That way, we can vary the steepness of the soft-Heaviside function independently of the relative interaction strengths and *vice versa*.

Eq 4 shows that *H* (*σ*_*j*_·*W*_*j*_) determines the steady state of the variable *Y*_*j*_ as a fraction of the total amount *Y*_*j*,T_. If the total amount of the protein remains constant over time, *Y*_*j*,T_ is a constant parameter in the model. If the total amount of the protein changes in time, we can use a class-1 variable to keep track of *Y*_*j*,T_ while using a class-2 variable to keep track of the fraction of the protein that is in the active form.

Class-2 variables evolve to a steady state on a time scale that is proportional to *γ*_*j*_^−1^. Therefore, if *γ*_*j*_ is large, we can invoke the pseudo-steady state approximation for the class-2 variable:

$$Y_{j}=Y_{j,\text{T}}\cdot H(\sigma_{j}\cdot W_{j}).$$ (6)

Moreover, if both *γ*_*j*_ and *σ*_*j*_ are large, then the class-2 variable, *Y*_*j*_/*Y*_*j*,T_, behaves like a Boolean variable, switching rapidly between 0 and 1 in response to other components in the network (S1 Fig).

Eq 4 has been used to describe interactions in PRNs many times before [12, 20–23]. We use the soft-Heaviside function because biochemical reactions (such as phosphorylation and dephosphorylation) are nonlinear in nature and often show ultrasensitive, sigmoidal responses [24–26]. Different mechanisms have been proposed to give rise to ultrasensitivity, and different types of rate equations have been used to capture this response. For transcription factors binding to gene promoter regions, Hill functions are often used to express the highly nonlinear (ultrasensitive) response of gene transcription rate to transcription factor concentration. For post-translational modifications of proteins, a commonly used mechanism is zero-order ultrasensitivity, originally proposed by Goldbeter & Koshland (GK) [25]. The GK equation describes the steady-state activity of a protein modified by two Michaelis-Menten enzymes with opposing effects (activation and inactivation). More recently, multisite phosphorylation has received considerable attention as an alternative mechanism of ultrasensitivity in PRNs [26–28]. All these mechanisms ultimately lead to sigmoidal-like responses, similar to the soft-Heaviside function (S1 Fig). Therefore, we eschew specific assumptions about the molecular mechanisms of ultrasensitivity and simply use the soft-Heaviside function as a phenomenological law that captures the ultrasensitive responses that are characteristic of PRNs.

Class-3 variables describe the rapid association and dissociation of proteins in multi-subunit complexes. For example, if proteins Y and I bind together strongly in an inactive complex, then the number of Y molecules that are free (not bound to I) is given by

$$Y=\text{max}(0,Y_{\text{T}}-I_{\text{T}}\text{)}.$$ (7)

This equation is a good approximation if both association and dissociation rates are fast, and the equilibrium binding constant is large. The max function returns zero if the total amount of the protein, *Y*_T_, is less than the total amount of its inhibitor, *I*_T_, meaning that all of protein Y is sequestered in the complex. On the other hand, if *Y*_T_ > *I*_T_, then the free form of the protein, *Y*, is simply the excess of *Y*_T_ over *I*_T_.

If an inhibitor associates with two different proteins with comparable association and dissociation rates, the amounts of the free proteins can be approximated by

$$\begin{array}{l} Y_{1}=\text{max}\left(0,\frac{Y_{1,,\text{T}}}{Y_{1}{}_{,\text{T}}+Y_{2,\text{T}}}\cdot (Y_{1,\text{T}}+Y_{2,\text{T}}-I_{\text{T}})\right)\\ Y_{2}=\text{max}\left(0,\frac{Y_{2,\text{T}}}{Y_{1,\text{T}}+Y_{2,\text{T}}}\cdot (Y_{1,\text{T}}+Y_{2,\text{T}}-I_{\text{T}})\right)\\ I=\text{max}\left(0,I_{\text{T}}-Y_{1,\text{T}}-Y_{2,\text{T}}\right) \end{array}$$ (8)

where *I*_T_, *Y*_1,T_ and *Y*_2,T_ are the total amounts of the proteins, and *I*, *Y*_1_ and *Y*_2_ are the amounts of the free forms of the proteins. In Eq 8, we assume that the inhibitor is distributed equally between Y_1_ and Y_2_, according to the availability of the two proteins. If I binds much more strongly to Y_1_ than to Y_2_, then Eq 8 should be replaced by

$$\begin{array}{l} Y_{1}=\text{max}\left(0,Y_{1,\text{T}}-I_{\text{T}}\right)\\ Y_{2}=\text{min}\left\{\text{max}\left(0,Y_{1,\text{T}}+Y_{2,\text{T}}-I_{\text{T}}\right),Y_{2,\text{T}}\right\}\;\\ I=\text{max}\left(0,I_{\text{T}}-Y_{1,\text{T}}-Y_{2,\text{T}}\right) \end{array}$$ (8′)

In the following sections, we show how to use these three classes of variables as building blocks to construct an SCM of the molecular network controlling the cell division cycle in budding yeast. Because the full model is very complex, we approach it in a series of simpler steps.

### 2. The Start transition in budding yeast

Start is an event in G_1_ phase of the budding yeast cell cycle when a cell commits to a new round of DNA synthesis and mitosis. A crucial step of the Start transition is the translocation of Whi5, a stoichiometric inhibitor of SBF and MBF transcription factors, from nucleus to cytoplasm [29, 30]. SBF and MBF control the expression of *CLN2* and *CLB5* genes, which encode “cyclin” proteins Cln2 and Clb5, respectively. Cln2 and Clb5 bind to kinase subunits (Cdc28) to form heterodimers with “cyclin-dependent kinase” (CDK) activity. CDK activity generated at Start triggers initiation of DNA synthesis and bud emergence. Because kinase subunits are in excess over cyclin partners [31], CDK activity is determined solely by the abundance of cyclin proteins. For simplicity in illustrating the SCM approach for the Start transition, we combine Cln2- and Clb5-dependent kinase activities into a single variable, called ClbS. We also treat SBF and MBF as a single variable, called SBF.

**Fig 1:**
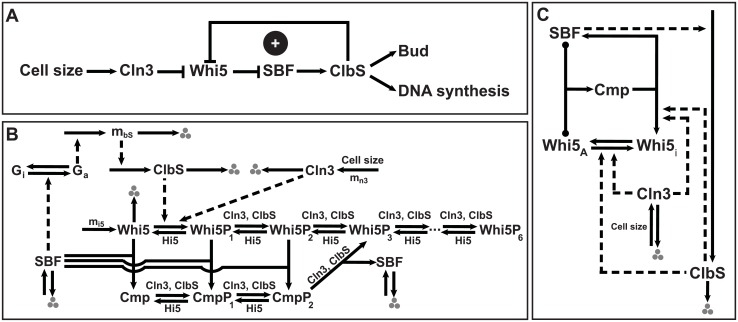
The Start transition. (A) Schematic diagram of the Start transition in budding yeast. In early G_1_, SBF is inactivated by its stoichiometric inhibitor, Whi5. As cell size increases, Cln3 accumulates and begins to phosphorylate Whi5. Phosphorylated Whi5 loses its ability to bind to SBF. As a result, SBF is free and promotes the production of ClbS (Cln2 and Clb5). ClbS exerts positive feedback on its own accumulation by further phosphorylating Whi5. The activation of SBF correlates with the onset of the Start transition. Subsequent accumulation of ClbS promotes both bud emergence and the G_1_/S transition. (B) Wiring diagram of the MultiP model. The first three forms of Whi5 (Whi5, Whi5P_1_, and Whi5P_2_) bind to SBF and inhibit its ability to activate the synthesis of ClbS. The higher phosphorylated forms are inactive and do not bind to SBF. The model also includes mRNA species for each protein component. (C) Wiring diagram of the standard component model. The 10 distinct forms of Whi5 in the MultiP model are replaced by two forms of Whi5 (active and inactive). For panels B and C, solid lines indicate chemical reactions (synthesis and degradation, phosphorylation and dephosphorylation, association and dissociation) and dashed lines indicate activatory or inhibitory influences of components on chemical reactions.

During normal cell cycle progression in budding yeast, the cell needs to grow sufficiently large to execute Start [32, 33]. The major players involved in “size control” of Start are Cln3 and Whi5. Whi5 prevents the Start transition by binding to and inhibiting SBF, and Cln3 promotes Start by phosphorylating and inactivating Whi5 [29,30]. The accumulation of Cln3 in G_1_ phase seems to depend on cell growth [34], and recent evidence suggests that Whi5 concentration is diluted out by cell growth [35]. As the cell grows, Cln3-dependent kinase phosphorylates Whi5, resulting in translocation of Whi5 from nucleus to cytoplasm and the release of its inhibition on SBF. Free SBF promotes the synthesis of ClbS, which stimulates its own expression by further phosphorylating Whi5. This positive feedback loop is thought to enforce the irreversible commitment of cells to the Start transition [36]. A schematic diagram illustrating the molecular basis of the Start transition is shown in Fig 1A.

Before constructing an SCM of the Start transition, we first describe a multisite phosphorylation (MultiP) model that will serve as a “reference point” for judging the adequacy of the SCM.

### 3. A multisite phosphorylation model of the Start transition

Our MultiP model is a simplified version of a model developed by Barik *et al*. [27] (see Fig 1B), who used mass-action kinetics to describe all the reactions involved in the Start transition and carried out detailed stochastic simulations based on Gillespie’s algorithm [13]. Our version of the model (see the equations in S1 Text) is governed by 20 molecular species, including both proteins and mRNAs (*m*_n3_, *m*_bS_, *m*_i5_, and *m*_hi5_ are mRNAs for Cln3, ClbS, Whi5, and Hi5, respectively). The 20 molecular species participate in 50 reactions, representing synthesis, degradation, phosphorylation, dephosphorylation, association, and dissociation of the species. Mass-action rate laws are used for all reactions. Each differential equation specifies how the number of molecules of each species changes with time.

Cell size (*V*) is assumed to increase exponentially. The rate of synthesis of each protein is assumed to be proportional to volume *V* × number of mRNA molecules *m* encoding the protein, because we assume that the number of ribosomes per cell increases proportionally to cell size *V*. In this way, we ensure that the concentration (*N*/*V*) of every constitutively expressed protein remains constant as the cell grows. The only exception is Cln3, whose synthesis rate—we assume—is proportional to the square of cell size (*V*^2^), as in Barik’s model [27]. This assumption, whose consequence is that the concentration of Cln3 increases exponentially as the cell grows, introduces a “size dependence” on the Start transition, which is needed to account for many properties of cell cycle progression in budding yeast. Although this assumption is sufficient to account for the observed size-dependence of yeast cell division, it is certainly not necessary. Other explanations are possible. Indeed, Schmoller *et al*. [35] have recently shown that Cln3 synthesis rate increases in direct proportion to *V*, so that its total concentration is nearly constant during G_1_ phase; and size dependence of the yeast cell cycle depends on diluting out Whi5, an inhibitor of Start, as the cell grows. This alternative will be explored in future versions of the model.

In Barik’s model [27], multisite phosphorylation of Whi5 is the source of nonlinearity necessary for the ultrasensitive Start transition. Whi5 is phosphorylated *in vivo* on ~10 CDK phosphorylation sites [37]. In Barik’s model Whi5 has seven phosphorylated states: Whi5, Whi5P_1_, Whi5P_2_, …, Whi5P_6_. In the model, the sites are phosphorylated sequentially and distributively [26]. The first three forms bind rapidly and strongly to SBF; the higher phosphorylated states (Whi5P_3_, …, Whi5P_6_) are inactive and unable to bind to SBF. Free SBF binds to and activates the ClbS gene (G_i_ + SBF **↔** G_a_). Cln3 and ClbS phosphorylate Whi5 (both free and in complex with SBF), while Whi5P_*i*_ species are dephosphorylated by an unspecified phosphatase, called Hi5 (“H” for phosphatase, “i5” for Whi5).

In Barik’s model [27], Whi5:SBF complexes are called Cmp, CmpP_1_, and CmpP_2_. Whi5P_2_ in the complex (CmpP_2_) is assumed not to get further phosphorylated. For Whi5P_2_ in the complex to be phosphorylated and thereby inactivated, Barik’s model supposes that Whi5P_2_ must first dissociate from CmpP_2_. We think this requirement is unnecessarily restrictive, so we allow the doubly-phosphorylated Whi5 in the complex (CmpP_2_) to be further phosphorylated, and the triply-phosphorylated Whi5 is assumed to immediately release SBF. We also assume, in the MultiP model, that the dissociation rate of SBF:Whi5P_*i*_ complexes is negligible.

### 4. An SCM for the Start transition

Fig 1C is the SCM representation of the Start transition. We assign the components in this diagram to the three classes of variables proposed above.

Cln3 and ClbS are described by class-1 variables:

$$\frac{\text{d}Cln3}{\text{d}t}=k_{\text{s},\text{n}3}\cdot V^{2}\cdot \underset{m_{\text{n}3}}{\underset{︸}{\frac{k_{\text{s},\text{mn}3}}{k_{\text{d},\text{mn}3}}}}-k_{\text{d},\text{n}3}\cdot Cln3$$ (9)

$$\frac{\text{d}ClbS}{\text{d}t}=k_{\text{s},\text{bS}}\cdot V\cdot \underset{m_{\text{bS}}}{\underset{︸}{\frac{k_{\text{s},\text{mbS}}}{k_{\text{d},\text{mbS}}}\cdot \underset{G_{\text{a}}}{\underset{︸}{\left(\frac{SBF}{SBF+\frac{k_{\text{d},\text{g}}\cdot V}{k_{\text{a},\text{g}}}}\right)}}}}-k_{\text{d},\text{bS}}\cdot ClbS.$$ (10)

In these equations, *V* = cell size, which we assume increases exponentially in time, *V*(*t*) = *V*_0_*e*^*μt*^, where *μ* is the specific growth rate of the cell. For each protein, its rate of synthesis is the product of (rate of translation, *k*_s,…_) × (number of mRNAs, *m*_…_) × (cell size, *V*). We explicitly account for the number of mRNAs in our synthesis rates (the factors labeled *m*_n3_ and *m*_bS_), as the mRNA number will be important later for representing noise terms. For simplicity, we assume (in the deterministic SCM) that mRNAs are always at their steady state levels. As in the MultiP model, we assume that the synthesis rate of Cln3 is proportional to the square of cell size (*V*^2^) so that the concentration of Cln3 (number/volume) will increase exponentially as the cell grows. The activity of the gene encoding ClbS, *G*_a_, depends on active SBF binding to the promoter region of the gene.

Since multisite phosphorylation of Whi5 gives rise to its ultrasensitive response to total CDK activity, we use a class-2 variable for the active form of Whi5 and a class-1 variable for the total amount of Whi5:

$$\frac{\text{d}Whi5_{\text{A}}}{\text{d}t}=\gamma \cdot (Whi5_{\text{T}}\cdot H(\sigma \cdot W_{\text{i5}})-Whi5_{\text{A}})$$ (11)

$$W_{\text{i}5}=\frac{\omega_{\text{dp},\text{i}5}}{V}\cdot Hi5-\frac{\omega_{\text{p},\text{i}5}}{V}\cdot Cln3-\frac{{\omega^{\prime }}_{\text{p},\text{i}5}}{V}\cdot ClbS$$ (12)

$$\frac{\text{d}Whi5_{\text{T}}}{\text{d}t}=k_{\text{s},\text{i}5}\cdot V\cdot \underset{m_{\text{i}5}}{\underset{︸}{\left(\frac{k_{\text{s},\text{mi}5}}{k_{\text{d},\text{mi}5}}\right)}}-k_{\text{d},\text{i}5}\cdot Whi5_{\text{T}}$$ (13)

$$\frac{\text{d}Hi5}{\text{d}t}=k_{\text{s},\text{hi}5}\cdot V\cdot \underset{m_{\text{hi}5}}{\underset{︸}{\left(\frac{k_{\text{s},\text{mhi}5}}{k_{\text{d},\text{mhi}5}}\right)}}-k_{\text{d},\text{hi}5}\cdot Hi5$$ (14)

where *H*(*x*) is the soft-Heaviside function defined earlier (see S1 Fig). We assume that the phosphorylation and dephosphorylation of Whi5 are independent of its binding state to SBF. In this way, we are able to use two differential equations (Eqs 11 and 13) to represent 10 distinct states of Whi5 and Whi5:SBF complexes. Here, *Whi5*_A_ (Eq 11) represents the total amount of active Whi5 both free and bound to SBF (it includes *Whi5*, *Whi5P*_1_, *Whi5P*_2_, *Cmp*, *CmpP*_1_, and *CmpP*_2_ in the MultiP model); whereas (*Whi5*_T_ –*Whi5*_A_) represents the other four inactive forms of Whi5 (*Whi5P*_3_ to *Whi5P*_6_). Hi5 (the phosphatase that re-activates Whi5) is described by a class-1 variable in Eq 14.

In Eq 12, the interaction coefficients *ω*_p,i5_, *ω′*_p,i5_ and *ω*_dp,i5_ are all positive numbers. The signs—positive or negative—in front of each term determine whether the interaction is activating or inhibiting (dephosphorylation or phosphorylation, in this case).

Assuming that binding between active forms of Whi5 and SBF is rapid and strong, we describe free SBF as a class-3 variable

$$SBF=\text{max}(0,\;SBF_{\text{T}}-Whi5_{\text{A}})\;.$$ (15)

This equation indicates that free SBF is equal to the excess of the total SBF over the total active Whi5, where *SBF*_T_ (Eq 16) is represented by a class-1 variable.

$$\frac{\text{d}SBF_{\text{T}}}{\text{d}t}=k_{\text{s},\text{bf}}\cdot V-k_{\text{d},\text{bf}}\cdot SBF_{\text{T}}\;$$ (16)

Because the original model of Barik *et al*. [27] did not have an mRNA species for SBF, we have not included mRNA for SBF in the SCM.

Using the SCM approach, we reduce the complexity of the Start model to seven differential equations plus one algebraic equation (from 20 equations in S1 Text for the MultiP model). The parameter values used in the SCM (Table 1) are inherited, for the most part, from the parameter values in Barik *et al*. [27]. The parameter values assigned by Barik *et al*. were estimated from experimental data wherever possible. For example, protein degradation rates were calculated from protein half-life measurements in the literature, and synthesis rate constants were assigned to agree with the average number of protein molecules observed for an asynchronous population of yeast cells growing on glucose medium. For phosphorylation and dephosphorylation reaction, there are no experimentally measured rate constants. Barik *et al*. assigned values to these rate constants so that their model compared well with the observations of Di Talia *et al*. [38], and we have done the same in assigning values to *γ*, *σ* and the *ω*’s in Eqs 11 and 12. (S1 Table specifies four additional parameter values needed for the MultiP model.)

**Table 1:** Parameter values for the standard component model of the Start transition.

Parameter	Description	Value
*k*_a,g_	Rate constant for association of SBF to *CLBS* promoter	0.25 fL molec^−1^ min^−1^
*k*_d,bf_	Rate constant for degradation of SBF	0.01 min^−1^
*k*_d,bS_	Rate constant for degradation of ClbS protein	0.1 min^−1^
*k*_d,g_	Rate constant for dissociation of SBF from *CLBS* promoter	12 min^−1^
*k*_d,hi5_	Rate constant for degradation of Hi5 phosphatase	0.01 min^−1^
*k*_d,i5_	Rate constant for degradation of Whi5	0.01 min^−1^
*k*_d,mbS_	Rate constant for degradation of *CLBS* mRNA	0.25 min^−1^
*k*_d,mhi5_	Rate constant for degradation of *HI5* mRNA	0.7 min^−1^
*k*_d,mi5_	Rate constant for degradation of *WHI5* mRNA	0.7 min^−1^
*k*_d,mn3_	Rate constant for degradation of *CLN3* mRNA	1 min^−1^
*k*_d,n3_	Rate constant for degradation of Cln3 protein	0.14 min^−1^
*k*_s,bf_	Rate constant for synthesis of SBF	1.53 molec fL^−1^ min^−1^
*k*_s,bS_	Rate constant for synthesis of ClbS protein	0.3 fL^−1^ min^−1^
*k*_s,hi5_	Rate constant for synthesis of Hi5 phosphatase	0.1275 fL^−1^ min^−1^
*k*_s,i5_	Rate constant for synthesis of Whi5 protein	0.715 fL^−1^ min^−1^
*k*_s,mbS_	Rate constant for synthesis of *CLBS* mRNA	11.5 molec min^−1^
*k*_s,mhi5_	Rate constant for synthesis of *HI5* mRNA	7 molec min^−1^
*k*_s,mi5_	Rate constant for synthesis of *WHI5* mRNA	5.25 molec min^−1^
*k*_s,mn3_	Rate constant for synthesis of *CLN3* mRNA	7.5 molec min^−1^
*k*_s,n3_	Rate constant for synthesis of Cln3 protein	0.0024 fL^−2^ min^−1^
<*m*_min,bS_>	Minimum number of *CLBS* mRNA molecules	1 molec
<*m*_min,hi5_>	Minimum number of *HI5* mRNA molecules	0
<*m*_min,i5_>	Minimum number of *WHI5* mRNA molecules	0
<*m*_min,n3_>	Minimum number of *CLN3* mRNA molecules	0
*γ*	Rate constant for Whi5 dephosphorylation	0.15 min^−1^
*μ*	Specific growth rate of cells	0.007 min^−1^
*σ*	Steepness of soft-Heaviside function	0.1
*ω*_dp,i5_	Interaction coeff for dephos’n of Whi5 by Hi5 phos’tase	0.12 fL molec^−1^
*ω*_p,i5_	Interaction coeff for phos’n of Whi5 by Cln3-dep kinase	6.2 fL molec^−1^
*ω*^'^_p,i5_	Interaction coeff for phos’n of Whi5 by ClbS-dep kinase	0.33 fL molec^−1^

In both models, initial conditions are set as follows: *Cln3* = *ClbS* = *SBF* = 0, and initial conditions for all other variables are set at their steady state levels (see Table 2 for the SCM and S2 Table for the MultiP model). In the MultiP model, at the beginning of the simulation, all Whi5 is in the unphosphorylated form and all SBF is complexed with Whi5. In the SCM, at the beginning of the simulation, all Whi5 is in the active form and all SBF is complexed with active Whi5.

**Table 2:** Initial conditions for simulations of the standard component model of the Start transition in Figs 3 and 5.

Variable	Number	Concentration
*ClbS*	0	0 nM
*Cln3*	0	0 nM
*Hi5*	1275	213 nM
*SBF*_T_	1530	255 nM
*Whi5*_A_	5363	894 nM
*Whi5*_T_	5363	894 nM
*V*	10 fL	

### 5. Stochastic version of SCM

Deterministic models are usually sufficient to predict the average behavior of a population of cells. However, at the level of individual cells, molecular regulatory networks operate under noisy conditions. A major source of noise inside cells are fluctuations of the numbers of molecules of biochemical species undergoing random events of synthesis and degradation. These inevitable fluctuations are referred to as “molecular noise”. Under the influence of such noise, the number of molecules, *N*, of a biochemical species fluctuates around the value predicted by the deterministic model. For a simple synthesis-degradation process, the variance of these fluctuations is equal to the mean value, ⟨*N*⟩, of the number of molecules [39]. Hence, the coefficient of variation (CV = standard deviation/mean) of the number of molecules is expected to be $1/\sqrt{\langle N\rangle }$, becoming proportionally larger as the mean number of molecules becomes smaller. For yeast cells, which carry only a few copies of some macromolecules (e.g., mRNA species), the fluctuations are potentially large.

To adapt an SCM for stochastic simulations, we add Langevin-type fluctuations to our equations for class-1 variables:

$$\frac{\Delta X_{i}}{\Delta t}=A_{i}-B_{i}\cdot X_{i}+\underset{\text{Protein}\;\text{noise}}{\underset{︸}{\sqrt{A_{i}+B_{i}\cdot X_{i}}\cdot \frac{\zeta_{1}(t)}{\sqrt{\Delta t}}}}+\underset{\text{mRNA}-\text{inherited}\;\text{noise}}{\underset{︸}{X_{i}\sqrt{2B_{i}\cdot \frac{1}{\langle m_{i}\rangle }\cdot \frac{B_{i}}{B_{i}+k_{\text{dm}}}}\cdot \frac{\zeta_{2}(t)}{\sqrt{\Delta t}}}}.$$ (17)

In this equation, molecular noise is attributed to random fluctuations at two levels: protein and mRNA. Fluctuations at the protein level are described by the chemical Langevin approximation [40]. *A*_*i*_ and *B*_*i*_ represent the protein synthesis and degradation rates as described in Eqs 2 and 3, respectively. *ζ*_1_ (*t*) and *ζ*_2_ (*t*) are independent random variables, each chosen from a normal distribution N(0, 1) with mean = 0 and standard deviation = 1. Δ*t* is the step size of the numerical integration. The last term in Eq 17 represents noise at the protein level inherited from fluctuations in mRNA molecules, and we next explain the origin of this term.

Since the number of mRNA molecules in a cell is typically much less than the numbers of protein molecules encoded by the mRNA, stochastic effects arising from mRNA fluctuations could contribute significantly to fluctuations in protein abundance. Instead of explicitly including mRNA dynamics in our SCM, as done in the Barik *et al*. model [27], we take an alternative approach. Pedraza and Paulsson [39] have shown that the square of the CV of protein numbers caused by mRNA fluctuations (at steady state) follows the equation

$${\text{CV}}_{\text{mRNA}-\text{inherited}\;\text{noise}}^{2}=\frac{1}{\langle m\rangle }\cdot \frac{\tau_{\text{m}}}{\tau_{\text{m}}+\tau_{\text{p}}},$$ (18)

Where ⟨*m*⟩ is the average number of mRNA molecules at steady state, and *τ*_m_ and *τ*_p_ are half-lives of mRNAs and proteins, respectively. The last term of Eq 17 was derived to approximate the CV^2^ predicted by Eq 18 under steady state conditions for the protein and mRNA levels (see S2 Text for the derivation). In Eq 17, *k*_dm_ is the rate constant for mRNA degradation; so the term *B*_*i*_ /(*B*_*i*_ *+ k*_dm_) is equal to *τ*_m_ /(*τ*_m_ + *τ*_p_) in Eq 18.

There is still a problem with the mRNA noise term in Eq 17. When ⟨*m*_*i*_⟩ is small (e.g., the case of a low level of the transcription factor SBF in Eq 10), the Langevin approximation breaks down and the noise term becomes very large. To avoid this, we replace ⟨*m*_*i*_⟩ with ⟨*m*_*i*_⟩ + ⟨*m*_min,*i*_⟩ in our simulation, where ⟨*m*_min,*i*_⟩ is assumed to be a minimum number of mRNA molecules always present in the cell. Eq 17 then becomes

$$\begin{array}{l} \frac{\Delta X_{i}}{\Delta t}=A_{i}-B_{i}\cdot X_{i}+\underset{\text{Protein}\;\text{noise}}{\underset{︸}{\sqrt{A_{i}+B_{i}\cdot X_{i}}\cdot \frac{\zeta_{1}(t)}{\sqrt{\Delta t}}}}\\ \;\;\;+\underset{\text{mRNA}-\text{inherited}\;\text{noise}}{\underset{︸}{X_{i}\sqrt{2B_{i}\frac{1}{\langle m_{i}\rangle +\langle m_{\text{min},i}\rangle }\cdot \frac{B_{i}}{B_{i}+k_{\text{dm}}}}\cdot \frac{\zeta_{2}(t)}{\sqrt{\Delta t}}}}, \end{array}$$ (19)

We treat ⟨*m*_min,*i*_⟩ as a parameter, and its value may vary from one mRNA species to another.

We do not incorporate protein and mRNA noise terms into variables of classes 2 and 3. We assume that protein phosphorylation and dephosphorylation reactions and the association and dissociation of protein complexes occur fast enough to bring any fluctuations quickly back to the average dynamics.

To create a stochastic SCM for the budding yeast Start transition, we modify the equations of class-1 variables by adding Langevin-type noise terms, as in Eq 19. For example, the stochastic equation for Cln3 is:

$$\begin{array}{l} \frac{\Delta Cln3}{\Delta t}=A_{\text{n}3}-B_{\text{n}3}\cdot Cln3+\sqrt{A_{\text{n}3}+B_{\text{n}3}\cdot Cln3}\cdot \frac{\zeta_{1}(t)}{\sqrt{\Delta t}}\\ +Cln3\sqrt{2B_{\text{n}3}\frac{1}{\langle m_{\text{n}3}\rangle +\langle m_{\text{min},\text{n}3}\rangle }\cdot \frac{B_{n3}}{B_{\text{n}3}+k_{\text{d},\text{mn}3}}}\cdot \frac{\zeta_{2}(t)}{\sqrt{\Delta t}}\\ A_{\text{n}3}=k_{\text{s},\text{n}3}\cdot V^{2}\cdot \frac{k_{\text{s},\text{mn}3}}{k_{\text{d},\text{mn}3}}\\ B_{\text{n}3}=k_{\text{d},\text{n}3}\\ \langle m_{\text{n}3}\rangle =\frac{k_{\text{s},\text{mn}3}}{k_{\text{d},\text{mn}3}}, \end{array}$$ (20)

where *A*_n3_ and *B*_n3_ represent the synthesis and degradation rates of Cln3 protein, and ⟨*m*_n3_⟩ is the average number of *CLN3* mRNA molecules calculated at steady state. ⟨*m*_min,n3_⟩ is a parameter representing the minimum number of *CLN3* mRNA molecules present in the cell. The full list of stochastic equations is given in S3 Text.

Barik *et al*. [27] used Gillespie's SSA to simulate all chemical reactions in their stochastic model. They assumed that hyper-phosphorylation of Whi5 corresponds to its nuclear export (as an indication of the Start transition). Their model accurately predicted the timing of the transition and its dependence on cell size at birth, when compared with experimental observations of Di Talia *et al*. [38].

In the next section, we compare deterministic and stochastic simulations of our SCM to deterministic and stochastic simulations of the MultiP model in S1 Text, and we show that the SCM, a model considerably less complex than MultiP, accounts for most of the dynamic properties of the system.

### 6. Comparisons of the Start model simulations

**Fig 2:**
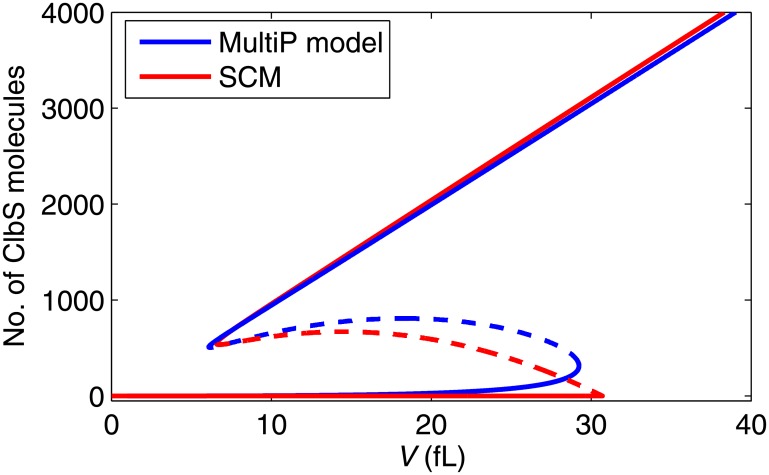
One-parameter bifurcation diagram. The steady-state number of ClbS molecules is plotted as a function of (fixed) cell volume. Solid line: stable steady states; dashed line: unstable steady states; blue lines: multisite phosphorylation (MultiP) model; red lines: standard component model (SCM). Both models exhibit a region of bistability between *V* ≈ 6 fL and *V* ≈ 30 fL. The right bifurcation point (at *V* ≈ 30 fL) corresponds to the threshold size for the Start transition.

To validate our approach, we compare qualitative and quantitative aspects of the SCM to the MultiP model. Fig 2 shows the one-parameter bifurcation curves for both models, in which we plot the steady-state number of ClbS molecules as a function of cell volume (*V*) as the bifurcation parameter. Both models exhibit bistability within a range of *V* from ~6 fL to ~30 fL. Hence, both models agree on cell size at the Start transition (~30 fL) for wild-type cells. Newborn cells in G_1_ phase of the cell cycle are captured by the stable steady state with ClbS level very low. They must grow to a size of ~30 fL, before the lower stable steady state disappears at a saddle-node bifurcation point. For *V* > 30 fL, the number of ClbS molecules rises rapidly to the high steady-state level, corresponding to the Start transition. In S2 Fig, we use two-parameter bifurcation diagrams to show how the SCM and the MultiP model behave when the synthesis rates of Cln3 and ClbS are varied at various (fixed) cell size.

**Fig 3:**
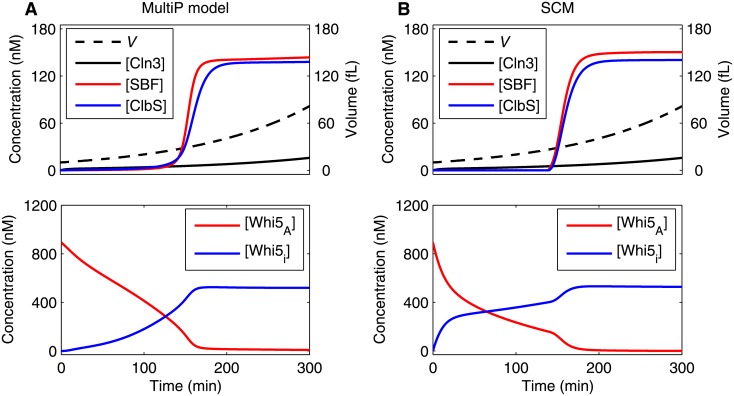
Deterministic trajectories simulated by the MultiP model and the SCM of the Start transition. Both models are simulated for 300 min, starting with *V* = 10 fL at *t* = 0. Initial conditions for the SCM are specified in Table 2 and for the MultiP model in S2 Table. In these panels we plot concentration (in nM), which is calculated from molecule number and volume (in fL) by the formula “concentration” = “number”/(0.6 × “volume in fL”). In the MultiP model, *Whi5*_A_ = *Whi5* + *Whi5P*_1_ + *Whi5P*_2_ + *Cmp* + *CmpP*_1_ + *CmpP*_2_. In both models, *Whi5*_i_ = *Whi5*_T_ –*Whi5*_A_. The changes in *Whi5*_A_ and *Whi5*_i_ over the first 100 min are quite different in the two models because their descriptions of the Whi5 activation process are quite different. Nonetheless, they predict similar timing for the cell cycle transitions. We presume that the Start and G_1_/S transitions occur when [SBF] = 15 nM and [ClbS] = 37.5 nM, respectively. (These values are 50% of the maximum concentrations from the original MultiP model [27], not 50% of the final concentrations shown in this figure). The MultiP model (left panels) executes the Start and G_1_/S transitions at *t* = 142 min and *t* = 152 min, respectively. The SCM (right panels) executes the Start and G_1_/S transitions at *t* = 145 min and *t* = 153 min, respectively.

Next we compare time-series dynamics of the two models in their deterministic formulations (i.e., simulations of the nonlinear ODEs). In Fig 3, we show simulations of a cell that starts with *V* = 10 fL at *t* = 0. The two models show very similar time courses for the control proteins. As in the multisite phosphorylation paper [27], we mark the Start transition as the time when the concentration of active SBF increases above 15 nM and the G_1_/S transition as the time when ClbS concentration increases above 37.5 nM. Concentration in nM is calculated from molecule number *N* and volume *V* in fL by the formula

$$\text{concentration}\;(\text{nM})\;=\;\frac{\text{number}\;\text{of}\;\text{molecules}}{N_{A}\;({\text{mol}}^{-1})\;\times \;V\;(\text{fL})}\times 10^{9}\;\frac{\text{nmol}}{\text{mol}}\times 10^{15}\;\frac{\text{fL}}{\text{L}}\;=\;\frac{N}{0.6\;V(\text{fL})}$$

where *N*_A_ is Avogadro’s number, 6.02 × 10^23^.

**Fig 4:**
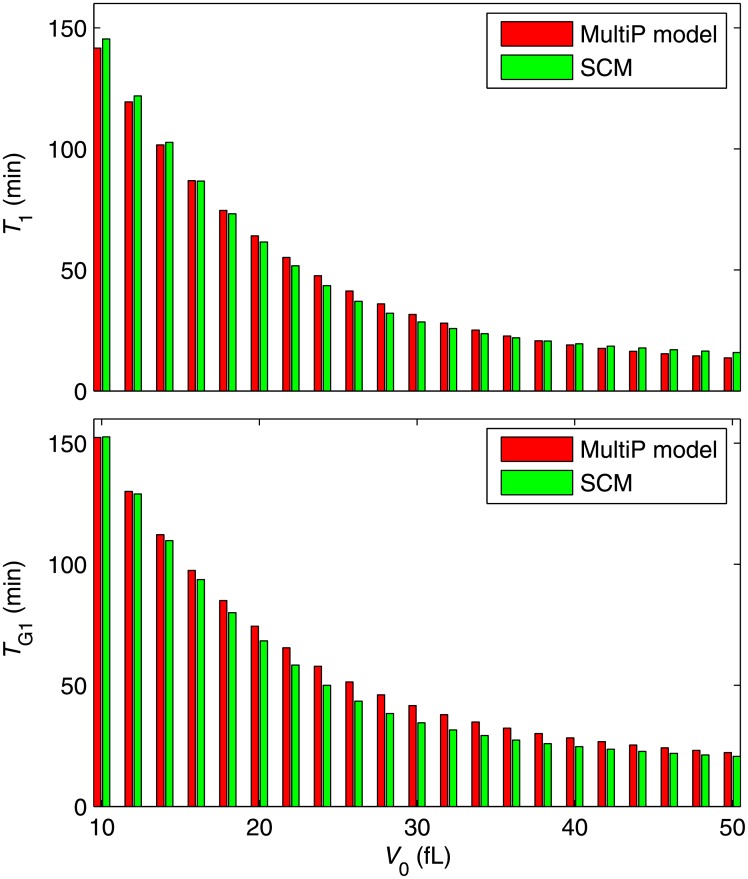
Deterministic simulations of the relation between initial cell size (*V*_0_) and cell age at the Start transition (*T*_1_) (upper panel) and cell age at the G_1_/S transition (*T*_G1_) (lower panel) for the Start models. Red bars: MultiP model; green bars: SCM. The left-most bars of the figure correspond to the time-series simulations in Fig 3.

Since it is well known that the smaller is cell size at birth the longer is the time to initiate bud formation and to enter S phase, we measure the durations from birth (*t* = 0) to the Start transition (*T*_1_) and to the G_1_/S transition (*T*_G1_) for various values of initial volume, *V*_0_. In Fig 4, we plot *T*_1_ and *T*_G1_ as functions of initial cell size. (The left-most bars of the figure correspond to the time-series simulations in Fig 3). The results show that the SCM is quantitatively comparable to the more complex MultiP model. The figure also shows that *T*_1_ and *T*_G1_ decrease as birth size increases, consistent with experimental observations [38]. The gap between Start and the G_1_/S transition, *T*_2_ = *T*_G1_ –*T*_1_, is small in both models (S3 Fig) and nearly independent of birth size [38].

**Fig 5:**
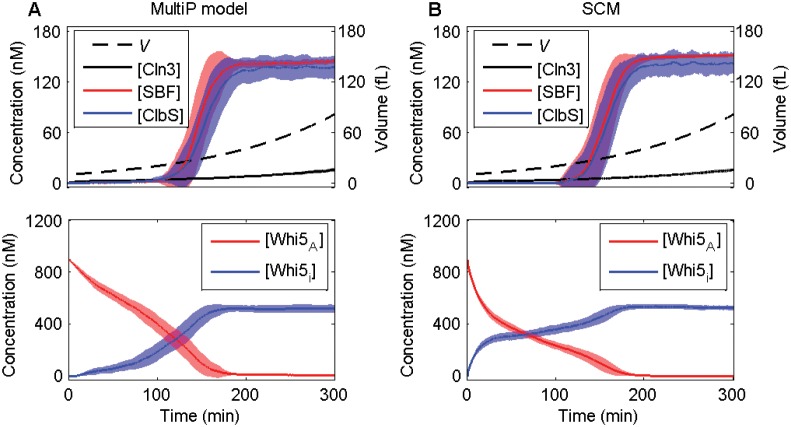
Stochastic trajectories generated by the MultiP model and the SCM of the Start transition. Means (lines) and standard deviations (shadows) calculated from 100 independent trajectories are shown for each model, starting with *V* = 10 fL at *t* = 0. Initial conditions for the SCM are specified in Table 2 and for the MultiP model in S2 Table. As in Fig 3 we plot “concentration in nM” = “number of molecules”/(0.6 × “volume in fL”). The MultiP model is simulated by Gillespie’s SSA and the SCM is simulated by the chemical Langevin approach described in the text.

**Fig 6:**
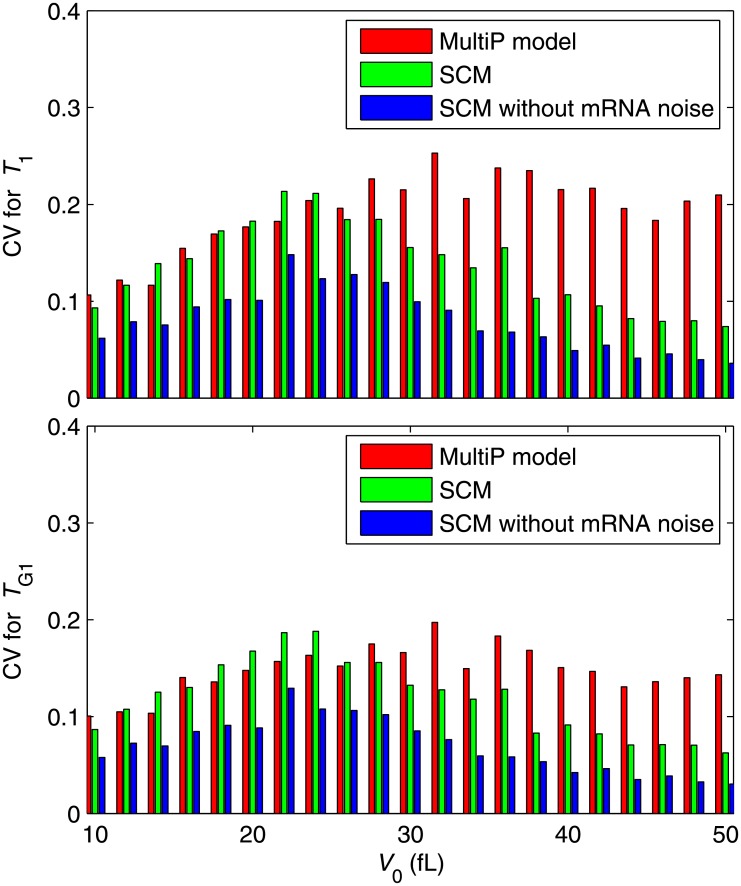
Stochastic simulations of *T*_1_ and *T*_G1_ for the Start models. As in Fig 4, we compute the times from birth (*t* = 0, *V* = *V*_0_) to the Start transition (*T*_1_, when [SBF] = 15 nM for the first time; upper panel) and to the G_1_/S transition (*T*_G1_, when [ClbS] = 37.5 nM for the first time; lower panel). For each model we compute 100 stochastic trajectories and calculate the mean and standard deviation of the time to the event. The mean times agree well with the deterministic simulations in Fig 4. Here we plot the coefficient of variation (CV = standard deviation/mean) of the times, in order to judge how well stochastic CLE simulations of the SCM (green bars) compare with SSA simulations of the MultiP model (red bars). Clearly the SCM under-estimates the variability of the transitions at large birth size. Removing mRNA noise from the SCM (blue bars) makes matters worse, as expected.

**Fig 7:**
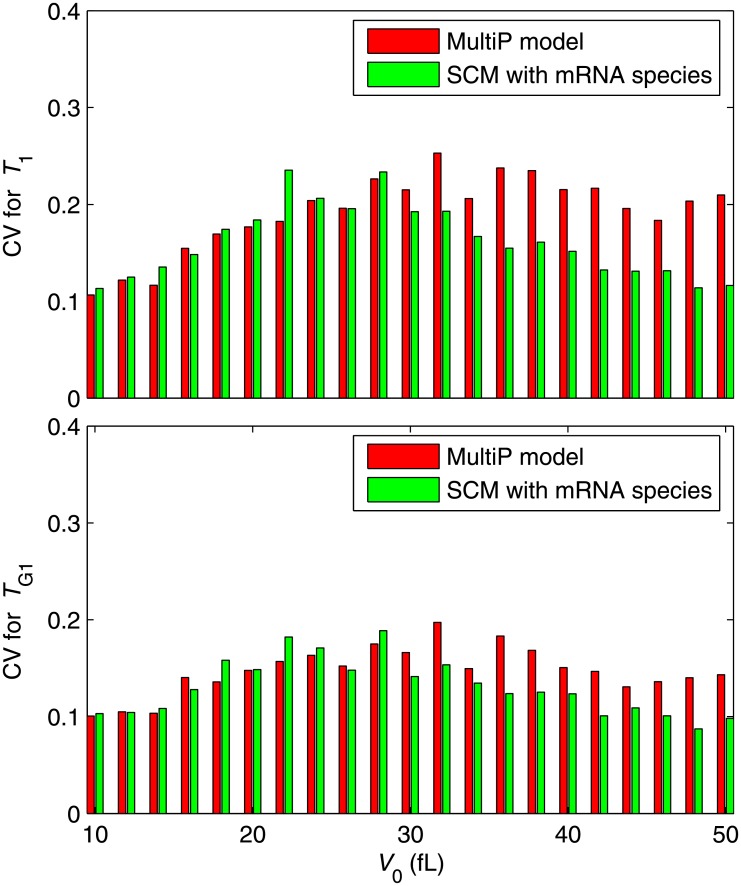
Stochastic simulations of *T*_1_ and *T*_G1_ for the Start SCM with explicit account of fluctuating mRNA species. As in Fig 6, except now we have added synthesis and degradation of mRNA species explicitly to the SCM. The remaining discrepancies are attributable in part to differences between the models and in part to simulating mRNA noise by CLE rather than SSA.

Next we study stochastic properties of the models. The MultiP model is simulated by SSA [13] while the SCM is simulated by the CLE approximation described above. S4 Fig compares the steady-state distributions of Cln3, SBF and ClbS at fixed volume for the MultiP model and the SCM. Fig 5 shows envelopes of sample trajectories from stochastic simulations of both models, starting with *V* = 10 fL at *t* = 0. Distributions of *T*_1_ and *T*_G1_ can be computed for the stochastic models as functions of *V*_0_. For each value of *V*_0_, we do 100 independent simulations of each model and compute the average values and standard deviations of *T*_1_ and *T*_G1_. The average values show similar patterns to the deterministic simulations in Fig 4 (see S5 Fig). The coefficient of variation (CV) is shown in Fig 6 and the distributions of *T*_1_ and *T*_G1_ are shown in S6 Fig. The results show that the noise intensities of both models agree well at small *V*_0_ but not at large *V*_0_ (red and green bars in Fig 6). Our SCM without mRNA fluctuations (i.e., discarding the mRNA-inherited noise term from the equations) shows less intensity of noise (blue bars in Fig 6), indicating that mRNA fluctuations are important. By expanding the SCM to include mRNA synthesis and degradation explicitly and simulating mRNA fluctuations using CLEs (see equations in S4 Text), we obtained much better agreement with the fully stochastic MultiP model (Fig 7), but at a cost of considerably more complexity.

With this simple model of the Start transition in budding yeast, we have shown that the SCM approach can capture essential dynamical features of a complex control network in quantitative detail. Encouraged by these results, we built an SCM of the full network of molecular controls of the budding yeast cell cycle, as described in the next section.

### 7. An SCM for the budding yeast cell cycle control system

**Fig 8:**
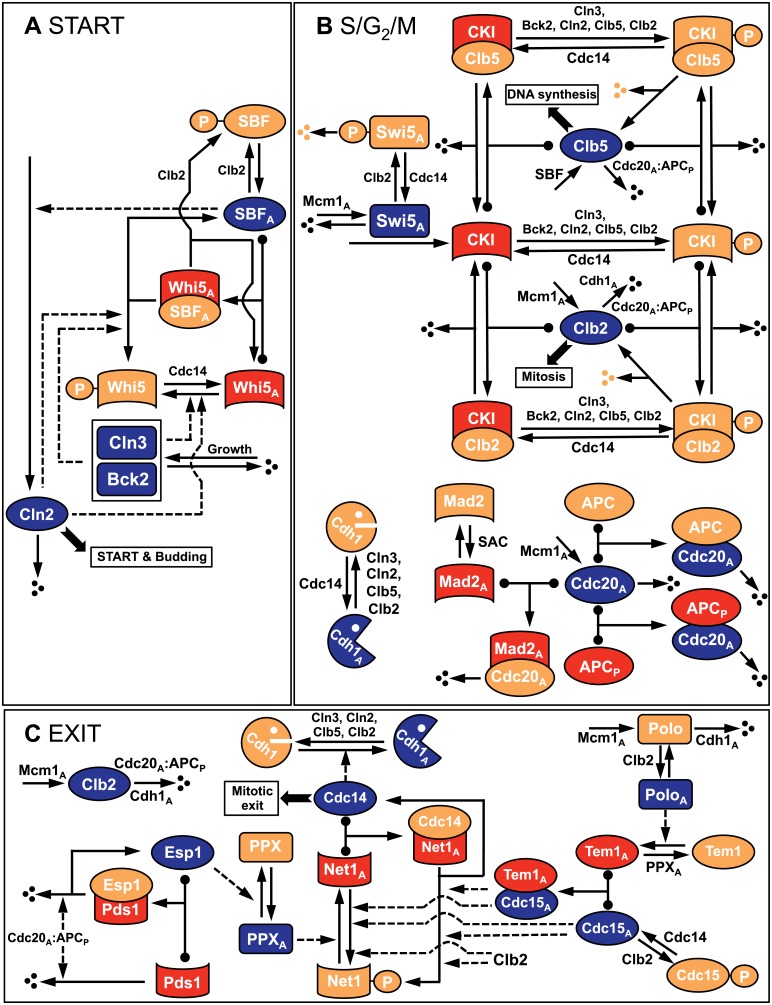
Wiring diagram of the full cell cycle control network in budding yeast. The network consists of three major modules: Start, S/G_2_/M, and Exit. Red and blue icons: active forms of components; orange icons: inactive forms. Solid lines: chemical reactions (synthesis and degradation, phosphorylation and dephosphorylation, association and dissociation); dashed lines: activatory or inhibitory influences of components on chemical reactions. T-shaped reaction arrows with black circles on the reactants side of the arrow indicate reversible association of two proteins to form a complex. T-shaped arrows without black circles represent irreversible reactions. Not all reactions are shown on this figure; see the equations in Table 4 for complete details.

Our cell cycle model (Fig 8) is based on the interaction network proposed in [41] with some modifications in the Start transition (by adding the inhibition of SBF by Whi5) and mitotic exit (by adding the stimulatory effects of Polo kinase and separase Esp1 on Cdc14 release), according to the models proposed in [36] and [42], respectively. In the model of Chen *et al*. [41] (Chen-2004, hereafter), each component is represented in terms of a concentration that has been scaled to a dimensionless number, called its normalized concentration, [] _n_. Cell volume, *V*_n_, is also dimensionless. In order to compare SCM simulations directly to Chen-2004, we switch from numbers of molecules to normalized concentrations, using the “characteristic concentrations” listed in Table 3. To be more specific, [S]_n_ = [S]/*c*_S_ = *N*_S_/(0.6 *V*∙c_S_), where [S] = concentration of species S in nM, *c*_S_ = characteristic concentration of S in nM, *N*_S_ = number of S molecules in volume *V* (in fL), and *V* = *V*_n_ c_vol_.

**Table 3:** Variables, initial values and characteristic concentrations for the standard component model of the full cell cycle control system.

Variable*	Description	Class	Initial Value	Characteristic Concentration
[APC_P_]_n_	Active (phosphorylated) form of APC	2	0.1216	150 nM
[Bck2]_n_	Total concentration of Bck2	1	0.0308	40 nM
[BUD]_n_	Progress to bud emergence	-	0.0488	-
[Cdc14]_n_	Active form of Cdc14 phosphatase	3	1.8914	18 nM
[Cdc15_A_]_n_	Active form of Cdc15 kinase	2	0.9823	8 nM
[Cdc20_T_]_n_	Total Cdc20, an APC partner	1	1.2422	150 nM
[Cdc20_A_]_n_	Active form of Cdc20	3	0.7925	
[Cdc20_A_:APC_P_]_n_	Complex between Cdc20_A_ and APC_P_	3	0.1216	
[Cdc20_A_:APC]_n_	Complex between Cdc20_A_ and APC	3	0.6710	
[Cdh1_A_]_n_	Active form of Cdh1, an APC partner	2	0.9574	150 nM
[CKI_P_]_n_	Phosphorylated forms of Sic1 & Cdc6	2	0	
[CKI_T_]_n_	Total cyclin inhibitors Sic1 & Cdc6	1	0.4012	40 nM
[Clb2]_n_	Active forms of cyclins Clb1 & Clb2	3	0.1011	
[Clb2_T_]_n_	Total cyclins Clb1 & Clb2	1	0.2687	40 nM
[Clb5]_n_	Active forms of cyclins Clb5 & Clb6	3	0.1412	
[Clb5_T_]_n_	Total cyclins Clb5 & Clb6	1	0.3752	40 nM
[Cln2]_n_	Total cyclins Cln1 & Cln2	1	0.1343	40 nM
[Cln3]_n_	Total cyclin Cln3	1	0.0757	40 nM
[Esp1]_n_	Active form of separase	3	0.4706	3.3 nM
[Mad2_A_]_n_	Active form of Mad2, a spindle assembly checkpoint protein	2	0.4497	150 nM
[Mcm1_A_]_n_	Active form of transcr factor for Clb2	2	0	100 nM
[Net1_A_]_n_	Active form of inhibitor of Cdc14	2	0.1086	18 nM
[ORI]_n_	Progress to DNA synthesis	-	0.0710	-
[Pds1_T_]_n_	Securin, an inhibitor of Esp1	1	0.0294	3.3 nM
[Polo_A_]_n_	Active form of Cdc5 kinase	2	0.2073	
[Polo_T_]_n_	Total Cdc5 kinase	1	0.2915	100 nM
[PPX_A_]_n_	Active form of a phosphatase	2	0.0128	100 nM
[SBF]_n_	Active (free) form of SBF transcription factor	3	0	22 nM
[SBF_A_]_n_	Unphosphorylated form of SBF	2	0.6560	
[SPN]_n_	Progress to spindle assembly	-	0.0389	
[Swi5_A_]_n_	Active form of Swi5	2	0.6333	
[Swi5_T_]_n_	Total Swi5, transcription factor of CKI	1	0.6333	57.5 nM
[Tem1_A_]_n_	Active form of Tem1, a G-protein kinase	2	0.8592	8 nM
[Tem1_A_:Cdc15_A_]_n_	Complex between Tem1_A_ and Cdc15_A_	3	0.8592	
[Whi5_A_]_n_	Active (unphosphorylated) form of Whi5	2	1.7238	22 nM
*V*n	Cell size (in normalized volume unit)	-	1.1460	28 fL

* […]_n_ refers to normalized (dimensionless) concentration variables

In budding yeast, as in all eukaryotes, cyclin/Cdk complexes are the main regulators of cycle progression. The kinase subunit, Cdc28, being far more abundant than all cyclin proteins, is always available to bind to any cyclins present in the cell [31]. Therefore, we use concentrations of cyclins to indicate the activities of cyclin/Cdc28 complexes (neglecting Cdc28 itself in the model). As in previous models, components that have similar functions are treated as single variables; for instance, “SBF” stands for SBF and MBF, “Cln2” for Cln1 and Cln2, “Clb2” for Clb1 and Clb2, “Clb5” for Clb5 and Clb6, and “CKI” for Sic1 and Cdc6.

For the cell cycle control system, class-1 variables include proteins whose concentrations change slowly over time due to the activities of transcription factors and proteolytic enzymes. This class includes, for example, the total concentrations of cyclin proteins (Cln2, Cln3, Clb2, and Clb5) and the total concentration of the stoichiometric inhibitor CKI. The synthesis of Cln2 and Clb5 is regulated by the transcription factor SBF, the synthesis of Clb2 is regulated by the transcription factor Mcm1, and the synthesis of CKI is controlled by the transcription factor Swi5. The degradation of Clb2 and Clb5 is regulated by the proteolytic factors Cdc20 and Cdh1 in association with an E3 ubiquitin ligase called the Anaphase Promoting Complex (APC).

The properties of many cell cycle-control proteins can be regulated by phosphorylation and dephosphorylation. For instance, phosphorylated Whi5 is a less potent inhibitor of SBF, and phosphorylated CKI is more susceptible to proteolysis. We use class-2 variables to represent the fractions of these proteins that are in “active” forms. The regulation of protein activity by phosphorylation and dephosphorylation is believed to be essential for the bistable switches that govern cell cycle transitions [6, 27, 36, 41, 43–45]. Class-2 variables capture the nonlinear behavior of these reactions in terms of the “soft-Heaviside” functions built into the SCM formulation.

Class-3 variables represent the free forms of proteins that form tight complexes with stoichiometric binding partners. For example, both Clb5 and Clb2 are inhibited by binding to CKI, as is Cdc14 inhibited by binding to Net1. In these cases, the “max” function employed by SCMs is a reasonable approximation because stoichiometric inhibitors usually bind strongly and rapidly to their partners. This strong-binding assumption is part of the original ODE model as well [41].

In Fig 8 we have divided the regulatory network into three modules (Start, S/G_2_/M, and Exit), which provides a useful framework for constructing the SCM. In Table 3 we classify the regulatory proteins into the three classes of SCM variables, and in Table 4 we translate Fig 8 into the differential and algebraic equations that define the dynamics of the network according to the SCM approach.

**Table 4:** Equations for the standard component model of the full cell cycle system.

**Cell Growth**	
$\frac{\text{d}V_{\text{n}}}{\text{d}t}=\mu \cdot V_{\text{n}}$	(21)
**Start Module**	
$\frac{\text{d}{\left[\text{Cln}3\right]}_{\text{n}}}{\text{d}t}=k_{\text{s},\text{cln}3}\cdot \frac{D_{\text{cln}3}\cdot V_{\text{n}}}{J_{\text{cln}3}+D_{\text{cln}3}\cdot V_{\text{n}}}-k_{\text{d},\text{cln}3}\cdot {\left[\text{Cln}3\right]}_{\text{n}}$	(22)
$\frac{\text{d}{\left[\text{Bck}2\right]}_{\text{n}}}{\text{d}t}=k_{\text{s},\text{bck}2}\cdot V_{\text{n}}-k_{\text{d},\text{bck}2}\cdot {\left[\text{Bck}2\right]}_{\text{n}}$	(23)
$\frac{\text{d}{\left[\text{Cln}2\right]}_{\text{n}}}{\text{d}t}=k_{\text{s},\text{cln}2}+k_{\text{s},\text{cln}2,\text{bf}}\cdot {\left[\text{SBF}\right]}_{\text{n}}-k_{\text{d},\text{cln}2}\cdot {\left[\text{Cln}2\right]}_{\text{n}}$	(24)
$\frac{\text{d}{\left[{\text{Whi5}}_{\text{A}}\right]}_{\text{n}}}{\text{d}t}=\gamma \cdot \left({\left[{\text{Whi5}}_{\text{T}}\right]}_{\text{n}}\cdot H\left(\sigma \cdot W_{\text{Whi5}}\right)-{\left[{\text{Whi5}}_{\text{A}}\right]}_{\text{n}}\right)$	(25)
$\begin{array}{l} W_{\text{Whi}5}=\omega_{\text{dp},\text{whis}5}+\omega_{\text{dp},\text{whis}5,14}\cdot {\left[\text{Cdc}14\right]}_{\text{n}}\\ \;\;\;\;-\omega_{\text{p},\text{whis}5,\text{n}3}\cdot {\left[\text{Cln}3\right]}_{\text{n}}-\omega_{\text{p},\text{whis}5,\text{k}2}\cdot {\left[\text{Bck}2\right]}_{\text{n}}-\omega_{\text{p},\text{whis}5,\text{n}2}\cdot {\left[\text{Cln}2\right]}_{\text{n}}-\omega_{\text{p},\text{whis}5,\text{b}5}\cdot {\left[\text{Clb}5\right]}_{\text{n}} \end{array}$	(26)
$\frac{\text{d}{\left[{\text{SBF}}_{\text{A}}\right]}_{\text{n}}}{\text{d}t}=\gamma \cdot \left({\left[{\text{SBF}}_{\text{T}}\right]}_{\text{n}}\cdot H\left(\sigma \cdot W_{\text{sbf}}\right)-{\left[{\text{SBF}}_{\text{A}}\right]}_{\text{n}}\right)$	(27)
$W_{\text{sbf}}=\omega_{\text{dp},\text{sbf}}-\omega_{\text{p},\text{sbf},\text{b}2}\cdot {\left[\text{Clb}2\right]}_{\text{n}}$	(28)
${\left[\text{SBF}\right]}_{\text{n}}=\text{max}\left(0,{\left[{\text{SBF}}_{\text{A}}\right]}_{\text{n}}-{\left[\text{Whi}5_{\text{A}}\right]}_{\text{n}}\right)$	(29)
$\frac{\text{d}{\left[\text{BUD}\right]}_{\text{n}}}{\text{d}t}=k_{\text{s},\text{bud},\text{e}}\cdot \left(e_{\text{bud},\text{n}3}\cdot {\left[\text{Cln}3\right]}_{\text{n}}+e_{\text{bud},\text{n}2}\cdot {\left[\text{Cln}2\right]}_{\text{n}}+e_{\text{bud},\text{b}5}\cdot {\left[\text{Clb}5\right]}_{\text{n}}\right)-k_{\text{d},\text{bud}}\cdot {\left[\text{BUD}\right]}_{\text{n}}$	(30)
**S/G**_**2**_**/M Module**	
$\begin{array}{l} \frac{\text{d}{\left[\text{Clb}5_{\text{T}}\right]}_{\text{n}}}{\text{d}t}=k_{\text{s},\text{clb}5}+k_{\text{s},\text{clb}5,\text{bf}}\cdot {\left[\text{SBF}\right]}_{\text{n}}\\ \;\;\;\;-\left(k_{\text{d},\text{clb}5}+k_{\text{d},\text{clb}5,20}\cdot {\left[\text{Cdc}20_{\text{A}}:{\text{ABC}}_{\text{P}}\right]}_{\text{n}}+k_{\text{d},\text{clb}5,20,\text{i}}\cdot {\left[\text{Cdc}20_{\text{A}}:\text{ABC}\right]}_{\text{n}}\right)\cdot {\left[\text{Clb}5_{\text{T}}\right]}_{\text{n}} \end{array}$	(31)
$\begin{array}{l} \frac{\text{d}{\left[\text{Clb}2_{\text{T}}\right]}_{\text{n}}}{\text{d}t}=\left(k_{\text{s},\text{clb}2}+k_{\text{s},\text{clb}2,\text{m}1}\cdot {\left[\text{Mcm}1_{\text{A}}\right]}_{\text{n}}\right)\cdot V_{\text{n}}-(k_{\text{d},\text{clb}2}+k_{\text{d},\text{clb}2,20}\cdot {\left[\text{Cdc}20_{\text{A}}:{\text{APC}}_{\text{P}}\right]}_{\text{n}}\\ \;\;\;\;\;\;+k_{\text{d},\text{clb}2,20,\text{i}}\cdot {\left[\text{Cdc}20_{\text{A}}:\text{APC}\right]}_{\text{n}}+k_{\text{d},\text{clb}2,\text{h}1}\cdot {\left[\text{Clb}1_{\text{A}}\right]}_{\text{n}})\cdot {\left[\text{Clb}2_{\text{T}}\right]}_{\text{n}} \end{array}$	(32)
$\frac{\text{d}{\left[{\text{CKI}}_{\text{T}}\right]}_{\text{n}}}{\text{d}t}=k_{\text{s},\text{cki}}+k_{\text{s},\text{cki},\text{swi}5}\cdot {\left[\text{Swi}5_{\text{A}}\right]}_{\text{n}}-k_{\text{d},\text{cki}}\cdot \left({\left[{\text{CKI}}_{\text{T}}\right]}_{\text{n}}-{\left[{\text{CKI}}_{\text{P}}\right]}_{\text{n}}\right)-k_{\text{d},\text{ckip}}\cdot {\left[{\text{CKI}}_{\text{P}}\right]}_{\text{n}}$	(33)
$\frac{\text{d}{\left[{\text{CKI}}_{\text{P}}\right]}_{\text{n}}}{\text{d}t}=\gamma_{\text{cki}}\cdot \left({\left[{\text{CKI}}_{\text{T}}\right]}_{\text{n}}\cdot H\left(\sigma \cdot W_{\text{cki}}\right)-{\left[{\text{CKI}}_{\text{P}}\right]}_{\text{n}}\right)-k_{\text{d},\text{ckip}}\cdot {\left[{\text{CKI}}_{\text{P}}\right]}_{\text{n}}$	(34)
$\begin{array}{l} W_{\text{cki}}=\omega_{\text{p},\text{cki},\text{n}3}\cdot {\left[\text{Cln}3\right]}_{\text{n}}+\omega_{\text{p},\text{cki},\text{k}2}\cdot {\left[\text{Bck}2\right]}_{\text{n}}+\omega_{\text{p},\text{cki},\text{n}2}\cdot {\left[\text{Cln}2\right]}_{\text{n}}+\omega_{\text{p},\text{cki},\text{b}5}\cdot {\left[\text{Clb}5\right]}_{\text{n}}\\ \;\;\;+\omega_{\text{p},\text{cki},\text{b}2}\cdot {\left[\text{Clb}2\right]}_{\text{n}}-\omega_{\text{dp},\text{cki}}-\omega_{\text{dp},\text{cki},14}\cdot {\left[\text{Cdc}14\right]}_{\text{n}} \end{array}$	(35)
${\left[\text{Clb5}\right]}_{\text{n}}=\text{max}\left(0,\frac{{\left[{\text{Clb5}}_{\text{T}}\right]}_{\text{n}}}{{\left[{\text{Clb5}}_{\text{T}}\right]}_{\text{n}}+{\left[{\text{Clb2}}_{\text{T}}\right]}_{\text{n}}}\cdot \left({\left[{\text{Clb5}}_{\text{T}}\right]}_{\text{n}}+{\left[{\text{Clb2}}_{\text{T}}\right]}_{\text{n}}-{\left[{\text{CKI}}_{\text{T}}\right]}_{\text{n}}\right)\right)$	(36)
${\left[\text{Clb2}\right]}_{\text{n}}=\text{max}\left(0,\frac{{\left[{\text{Clb2}}_{\text{T}}\right]}_{\text{n}}}{{\left[{\text{Clb5}}_{\text{T}}\right]}_{\text{n}}+{\left[{\text{Clb2}}_{\text{T}}\right]}_{\text{n}}}\cdot \left({\left[{\text{Clb5}}_{\text{T}}\right]}_{\text{n}}+{\left[{\text{Clb2}}_{\text{T}}\right]}_{\text{n}}-{\left[{\text{CKI}}_{\text{T}}\right]}_{\text{n}}\right)\right)$	(37)
$\frac{\text{d}{\left[\text{Swi}5_{\text{T}}\right]}_{\text{n}}}{\text{d}t}=k_{\text{s},\text{swi}5}+k_{\text{s},\text{swi}5,\text{m}1}\cdot {\left[\text{Mcm}1_{\text{A}}\right]}_{\text{n}}-k_{\text{d},\text{swi}5}\cdot {\left[\text{Swi}5_{\text{T}}\right]}_{\text{n}}$	(38)
${\left[{\text{Swi5}}_{\text{A}}\right]}_{\text{n}}={\left[{\text{Swi5}}_{\text{T}}\right]}_{\text{n}}\cdot H\left(\sigma \cdot W_{\text{swi5}}\right)$	(39)
$W_{\text{swi}5}=\omega_{\text{a},\text{swi}5,14}\cdot {\left[\text{Cdc}14\right]}_{\text{n}}-\omega_{\text{i},\text{swi}5,\text{b}2}\cdot {\left[\text{Clb}2\right]}_{\text{n}}$	(40)
$\frac{\text{d}{\left[\text{ORI}\right]}_{\text{n}}}{\text{d}t}=k_{\text{s},\text{ori},\text{e}}\cdot \left(e_{\text{ori},\text{b}5}\cdot {\left[\text{Clb}5\right]}_{\text{n}}+e_{\text{ori},\text{b}2}\cdot {\left[\text{Clb}2\right]}_{\text{n}}\right)-k_{\text{d},\text{ori}}\cdot {\left[\text{ORI}\right]}_{\text{n}}$	(41)
$\frac{\text{d}{\left[{\text{Cdh1}}_{\text{A}}\right]}_{\text{n}}}{\text{d}t}=\gamma \left({\left[{\text{Cdh1}}_{\text{T}}\right]}_{\text{n}}\cdot H\left(\sigma \cdot W_{\text{cdh1}}\right)-{\left[{\text{Cdh1}}_{\text{A}}\right]}_{\text{n}}\right)$	(42)
$\begin{array}{l} W_{\text{cdh}1}=\omega_{\text{a},\text{cdh}1}+\omega_{\text{a},\text{cdh}1,14}\cdot {\left[\text{Cdc}14\right]}_{\text{n}}\\ -\omega_{\text{i},\text{cdh}1,\text{e}}\cdot \left(\omega_{\text{i},\text{cdh}1,\text{n}3}\cdot {\left[\text{Cln}3\right]}_{\text{n}}+\omega_{\text{i},\text{cdh}1,\text{n}2}\cdot {\left[\text{Cln}2\right]}_{\text{n}}+\omega_{\text{i},\text{cdh}1,\text{b}5}\cdot {\left[\text{Clb}5\right]}_{\text{n}}+\omega_{\text{i},\text{cdh}1,\text{b}2}\cdot {\left[\text{Clb}2\right]}_{\text{n}}\right) \end{array}$	(43)
${\left[{\text{Mcm1}}_{\text{A}}\right]}_{\text{n}}={\left[{\text{Mcm1}}_{\text{T}}\right]}_{\text{n}}\cdot H\left(\sigma \cdot W_{\text{mcm1}}\right)$	(44)
$W_{\text{mcm1}}=\omega_{\text{a},\text{mcm}1,\text{b}2}\cdot {\left[\text{Clb}2\right]}_{\text{n}}-\omega_{\text{i},\text{mcm}1}$	(45)
$\frac{\text{d}{\left[{\text{APC}}_{\text{P}}\right]}_{\text{n}}}{\text{d}t}=\gamma_{\text{apc}}\left({\left[{\text{APC}}_{\text{T}}\right]}_{\text{n}}\cdot H\left(\sigma \cdot W_{\text{apc}}\right)-{\left[{\text{APC}}_{\text{P}}\right]}_{\text{n}}\right)$	(46)
$W_{\text{apc}}=\omega_{\text{a},\text{apc},\text{b}2}\cdot {\left[\text{Clb}2\right]}_{\text{n}}-\omega_{\text{i},\text{apc}}$	(47)
$\frac{\text{d}{\left[{\text{Mad2}}_{\text{A}}\right]}_{\text{n}}}{\text{d}t}=\gamma \cdot \left({\left[{\text{Mad2}}_{\text{T}}\right]}_{\text{n}}\cdot H\left(\sigma \cdot W_{\text{mad2}}\right)-{\left[{\text{Mad2}}_{\text{A}}\right]}_{\text{n}}\right)$	(48)
$W_{\text{mad2}}=\omega_{\text{a},\text{mad}2}\cdot B_{\text{udna}}\cdot \left(1-B_{\text{spc}}\right)-\omega_{\text{i},\text{mad}2}$	(49)
$\frac{\text{d}{\left[\text{SPN}\right]}_{\text{n}}}{\text{d}t}=k_{\text{s},\text{spn}}\cdot Heav\left({\left[\text{Clb2}\right]}_{\text{n}}-J_{\text{spn}}\right)-k_{\text{d},\text{spn}}\cdot {\left[\text{SPN}\right]}_{\text{n}},$	(50)
$\frac{\text{d}{\left[{\text{Cdc20}}_{\text{T}}\right]}_{\text{n}}}{\text{d}t}=k_{\text{s},\text{cdc}20}+k_{\text{s},\text{cdc}20,\text{m}1}\cdot {\left[{\text{Mcm1}}_{\text{A}}\right]}_{\text{n}}-k_{\text{d},\text{cdc}20}\cdot {\left[{\text{Cdc20}}_{\text{T}}\right]}_{\text{n}}$	(51)
${\left[{\text{Cdc20}}_{\text{A}}\right]}_{\text{n}}=\text{max}\left(0,{\left[{\text{Cdc20}}_{\text{T}}\right]}_{\text{n}}-{\left[{\text{Mad2}}_{\text{A}}\right]}_{\text{n}}\right)$	(52)
${\left[{\text{Cdc20}}_{\text{A}}\right]}_{\text{n}}=\text{min}\left({\left[{\text{Cdc20}}_{\text{A}}\right]}_{\text{n}},{\left[{\text{APC}}_{\text{P}}\right]}_{\text{n}}\right)$	(53)
${\left[{\text{Cdc20}}_{\text{A}}:\text{APC}\right]}_{\text{n}}=\text{min}\left({\left[{\text{Cdc20}}_{\text{A}}\right]}_{\text{n}}-{\left[{\text{Cdc20}}_{\text{A}}:{\text{APC}}_{\text{P}}\right]}_{\text{n}},\;{\left[{\text{APC}}_{\text{T}}\right]}_{\text{n}}-{\left[{\text{APC}}_{\text{P}}\right]}_{\text{n}}\right)$	(54)
**Exit Module**	
$\begin{array}{l} \frac{\text{d}{\left[\text{Pds}1_{\text{T}}\right]}_{\text{n}}}{\text{d}t}=k_{\text{s},\text{pds}1}-(k_{\text{s},\text{pds}1}\;+\;k_{\text{d},\text{pds}1,20}\cdot {\left[\text{Cdc}20_{\text{A}}:{\text{APC}}_{\text{P}}\right]}_{\text{n}}\\ \;\;\;\;\;\;+k_{\text{d},\text{pds}1,20,\text{i}}\cdot {\left[\text{Cdc}20_{\text{A}}:\text{APC}\right]}_{\text{n}}\cdot \;{\left[\text{Pds}1_{\text{T}}\right]}_{\text{n}} \end{array}$	(55)
${\left[\text{Esp1}\right]}_{\text{n}}=\;\text{max}(0,{\left[{\text{Esp1}}_{\text{T}}\right]}_{\text{n}}-{\left[{\text{Pds1}}_{\text{T}}\right]}_{\text{n}}$	(56)
$\frac{\text{d}{\left[{\text{PPX}}_{\text{A}}\right]}_{\text{n}}}{\text{d}t}=\gamma \cdot ({\left[{\text{PPX}}_{\text{T}}\right]}_{\text{n}}\cdot H(\sigma \cdot W_{\text{ppx}})-{\left[{\text{PPX}}_{\text{A}}\right]}_{\text{n}})$	(57)
$W_{\text{ppx}}=\omega_{\text{a},\text{ppx}}-\omega_{\text{i},\text{ppx},\text{p}1}\cdot {\left[\text{Esp1}\right]}_{\text{n}}$	(58)
$\frac{\text{d}{\left[{\text{Net1}}_{\text{A}}\right]}_{\text{n}}}{\text{d}t}=\gamma \cdot ({\left[{\text{Net1}}_{\text{T}}\right]}_{\text{n}}\cdot H(\sigma_{\text{net}1}\cdot W_{\text{net}1})-{\left[{\text{Net1}}_{\text{A}}\right]}_{\text{n}})$	(59)
$\begin{array}{l} W_{\text{net}1}=\omega_{\text{dp},\text{net}1}+\omega_{\text{dp},\text{net}1,14}\cdot {\left[\text{Cdc}14\right]}_{\text{n}}+\omega_{\text{dp},\text{net}1,\text{px}}\cdot {\left[{\text{PPX}}_{\text{A}}\right]}_{\text{n}}\\ \;\;\;\;\;-\omega_{\text{p},\text{net}1,\text{b}2}\cdot {\left[\text{C}1\text{b}2\right]}_{\text{n}}-\omega_{\text{p},\text{net}1,\text{en}}\cdot {\left[\text{Tem}1_{\text{A}}:\text{Cdc}15_{\text{A}}\right]}_{\text{n}}\\ \;\;\;\;\;-\omega_{\text{p},\text{net}1,15}\cdot ({\left[\text{Cdc}15_{\text{A}}\right]}_{\text{n}}:{\left[\text{Tem}1_{\text{A}}:\text{Cdc}15_{\text{A}}\right]}_{\text{n}}) \end{array}$	(60)
${\left[\text{Cdc14}\right]}_{\text{n}}=\text{max}(0,{\left[{\text{Cdc14}}_{\text{T}}\right]}_{\text{n}}-\rho_{14,\text{net}1}\cdot {\left[{\text{Net1}}_{\text{A}}\right]}_{\text{n}})$	(61)
$\frac{\text{d}{\left[{\text{Polo}}_{\text{T}}\right]}_{\text{n}}}{\text{d}t}=k_{\text{s},\text{polo},\text{m}1}\cdot {\left[{\text{Mcm1}}_{\text{A}}\right]}_{\text{n}}-(k_{\text{d},\text{polo}}+k_{\text{d},\text{polo},\text{h}1}\cdot {\left[{\text{Cdh1}}_{\text{A}}\right]}_{\text{n}})\cdot {\left[{\text{Polo}}_{\text{T}}\right]}_{\text{n}}$	(62)
$\frac{\text{d}{\left[{\text{Polo}}_{\text{A}}\right]}_{\text{n}}}{\text{d}t}=\gamma \cdot ({\left[{\text{Polo}}_{\text{T}}\right]}_{\text{n}}\cdot H(\sigma \cdot W_{\text{polo}})-{\left[{\text{Polo}}_{\text{A}}\right]}_{\text{n}})$	(63)
$W_{\text{polo}}=\omega_{\text{a},\text{polo},\text{b}2}\cdot {\left[\text{Clb2}\right]}_{\text{n}}-\omega_{\text{i},\text{polo}}$	(64)
$\frac{\text{d}{\left[{\text{Tem1}}_{\text{A}}\right]}_{\text{n}}}{\text{d}t}=\gamma_{\text{tem}1}\cdot ({\left[{\text{Tem1}}_{\text{T}}\right]}_{\text{n}}\cdot H(\sigma \cdot W_{\text{tem1}})-{\left[{\text{Tem1}}_{\text{A}}\right]}_{\text{n}})$	(65)
$W_{\text{tem}1}=\omega_{\text{a},\text{tem}1,\text{lo}}\cdot {\left[{\text{Polo}}_{\text{A}}\right]}_{\text{n}}-\omega_{\text{i},\text{tem}1}-\omega_{\text{i},\text{tem}1,\text{px}}\cdot {\left[{\text{PPX}}_{\text{A}}\right]}_{\text{n}}$	(66)
$\frac{\text{d}{\left[{\text{Cdc15}}_{\text{A}}\right]}_{\text{n}}}{\text{d}t}=\gamma \cdot ({\left[{\text{Cdc15}}_{\text{T}}\right]}_{\text{n}}\cdot H(\sigma \cdot W_{\text{cdc15}})-{\left[{\text{Cdc15}}_{\text{A}}\right]}_{\text{n}})$	(67)
$W_{\text{cds}15}=\omega_{\text{a},\text{cdc}15,14}\cdot {\left[\text{Cdc}14\right]}_{\text{n}}-\omega_{\text{i},\text{cdc}15}-\omega_{\text{i},\text{cdc}15,\text{b}2}\cdot {\left[\text{Clb}2\right]}_{\text{n}}$	(68)
${\left[{\text{Tem1}}_{\text{A}}:{\text{Cdc15}}_{\text{A}}\right]}_{\text{n}}=\text{min}({\left[{\text{Tem1}}_{\text{A}}\right]}_{\text{n}},{\left[{\text{Cdc15}}_{\text{A}}\right]}_{\text{n}})$	(69)
**Definitions:**	
$H\left(X\right)=1/\left(1+e^{-x}\right);\;Heav\left(x\right)=1,\;\text{if}\;x\geq 0,\;=\;\text{otherwise}$	

Rules:

1) Bud emerges when [BUD]_n_ = 1.

2) DNA synthesis starts when [ORI]_n_ = 1; provided that *B*_oriflag_ = 1. Then *B*_oriflag_ is reset to 0 and *B*_udna_ = 1.

3) Spindle assembly is complete and chromosomes are properly aligned when [SPN]_n_ = 1. Set *B*_spc_ = 1.

4) The cell divides asymmetrically between mother and daughter cells when [Clb2]_n_ drops below *K*_EZ_. The mother:daughter size ratio at birth, (1−*f*):*f*, is computed from the formula *f* = 0.3364 exp(22.2/*T*_d_), where *T*_d_ = mass-doubling time of the culture [43]. In glucose medium (*T*_d_ = 100 min) the ratio is 58:42 and in galactose and raffinose media (*T*_d_ = 150 min) the ratio is 61:39. At cell division, [BUD]_n_, [SPN]_n_, *B*_udna_ and *B*_spc_ are all reset to 0.

5) [ORI]_n_ is reset to 0 (origins of replication are relicensed) when [Clb2]_n_+[Clb5]_n_ drops below *K*_EZ2_. Set *B*_oriflag_ = 1.

#### Formulation of the Start module

Components involved in the Start transition (for a review, see [46]) are governed by Eqs 22–30. The molecular mechanism of the Start transition considered here (Fig 8A) is more complex than the test case (Fig 1C) considered earlier. First, instead of lumping Cln2 and Clb5 into one variable (ClbS), we separate them (Eqs 24 and 31) because they are controlled differently: unlike Clb5, Cln2 is not inhibited by CKI [47] and not degraded by Cdc20 [48]. Second, we add a component Bck2 (Eq 23), which is known to activate SBF in parallel to Cln3 [49, 50]. Third, the dephosphorylation of Whi5 by Hi5, an unknown phosphatase, is considered as a background dephosphorylation (*ω*_dp,whi5_) since we assume that the concentration of Hi5 is always constant. Finally, Cdc14, a component that is active in late mitosis, also contributes to dephosphorylation of Whi5 during mitotic exit [51].

Concentrations of Cln3, Bck2, and Cln2 are controlled by synthesis and degradation and described by class-1 variables (Table 4, Eqs 22, 23 and 24, respectively). The G_1_/S transition (Start) in budding yeast is known to be sensitively dependent on cell size [33], and Start is known to be strongly dependent on the expression of *CLN3* and *BCK2* genes [31, 34, 52]. To account for these facts, we assume that the synthesis rates of Cln3 and Bck2 proteins are proportional to cell size, *V*. (This assumption was also made in Chen-2004 [41] and in the stochastic model of Barik *et al*. [27].) The production of Cln2 is controlled by the SBF transcription factor.

The activity of SBF is controlled by binding to an inhibitor, Whi5 [29, 30], and by phosphorylation (inactivation) by Clb2 [53, 54]; see Table 4, Eqs 27, 28 and 29. In order to bind to SBF, Whi5 must also be in its unphosphorylated form. Therefore, functional SBF, [SBF]_n_ in Eq 29, is the amount of unphosphorylated SBF, denoted [SBF_A_]_n_, that is not bound to unphosphorylated Whi5, denoted [Whi5_A_]_n_. For simplicity, we assume that phosphorylated SBF does not bind to unphosphorylated Whi5.

In early G_1_, Clb2 level is low, so SBF is unphosphorylated, but it is not active because Whi5 is also unphosphorylated, and Whi5_A_ binds rapidly and strongly to SBF_A_ [29, 30]. As the cell grows, Cln3 and Bck2 accumulate and phosphorylate Whi5. Phosphorylated Whi5 dissociates from SBF and translocates from nucleus to cytoplasm [29, 30], leaving SBF free to do its job as a transcription factor for Cln2 and Clb5 (Table 4, Eqs 24 and 31. We assume that the phosphorylation and dephosphorylation of Whi5 are independent of its binding to SBF (Table 4, Eq 25).

Functional SBF promotes the synthesis of Cln2 and Clb5. As Cln2 accumulates, it further phosphorylates Whi5, enabling faster production of Cln2 [55]. Cln3, Cln2, and Clb5 all promote bud emergence [56]. The BUD variable represents progression to bud emergence, and we assume that a bud emerges when [BUD]_n_ = 1 (Table 4, Eq 30).

#### Formulation of the S/G_2_/M module

Fig 8B and Eqs 31–54 of Table 4 describe the S/G_2_/M module. CKI (the collective name of Sic1 and Cdc6) is a stoichiometric inhibitor that keeps, as we assume, Clb5 and Clb2 inactive during G_1_ phase. (In fact, Clb2 is inhibited by both Sic1 and Cdc6, whereas Clb5 is inhibited mainly by Sic1 and not by Cdc6 [47, 57, 58].) We track the total concentrations of Clb5, Clb2, and CKI by class-1 variables (Eqs 31, 32 and 33, respectively) and use class-3 variables to calculate free forms of Clb5 and Clb2 (Eqs 36 and 37). In these equations, we assume that CKI has comparable association and dissociation rates with both Clb2 and Clb5, so that CKI is distributed equally between them. (Again, note that this is not true for Cdc6 [57].)

The rise of Cln2 after the Start transition causes the phosphorylation and rapid degradation of CKI (Eqs 34 and 35) [59]. The falling level of CKI releases Clb5, which further phosphorylates CKI. Free Clb5 activates components responsible for DNA synthesis [60] (Eq 41). The ORI variable represents initiation of DNA replication, which starts (we assume) when [ORI]_n_ = 1.

Cln2 and Clb5 also inactivate Cdh1, a protein that degrades Clb2 during G_1_ phase [61, 62] (Eqs 42 and 43), allowing Clb2 to accumulate. However, at this point (in late G_1_-early S), the total concentration of Clb2 is low because its transcription factor, Mcm1, is inactive. Clb2 production ramps up as Clb2-dependent kinase activates its own transcription factor Mcm1 [53, 63] (Eqs 44 and 45). In addition, Clb2 phosphorylates and inactivates SBF [53, 54] (Eqs 27 and 28), thereby stopping synthesis of Cln2 and Clb5 and preparing the cell for mitotic exit.

Swi5 is the transcription factor for CKI [64] (Eq 33). We use a class-1 variable to track the total amount of Swi5 (Eq 38) and a class-2 variable to track its activity (Eq 39). Eq 39 calculates the activity of Swi5, [Swi5_A_]_n_, at steady state, assuming its phosphorylation and dephosphorylation are very rapid.

As the accumulation of Clb2 drives the cell into M phase, it also sets up conditions for mitotic exit by phosphorylating and activating the APC [65] (Eqs 46 and 47). APC activity—which requires the cooperation of an auxiliary subunit, Cdc20—is essential for chromosome segregation at the metaphase-anaphase transition [66, 67] and the initiation of mitotic cyclin degradation [68]. But, during early M phase, Cdc20 is kept inactive by the spindle assembly checkpoint (SAC) [69]. The SAC activates a checkpoint protein, Mad2, which sequesters and inactivates Cdc20. In our model, the Mad2-dependent checkpoint is invoked by the onset of DNA synthesis (when [ORI]_n_ = 1) and released once the mitotic spindle is fully assembled (when [SPN]_n_ = 1) (Eqs 48 and 49). The mitotic spindle starts to assemble, we assume, when the concentration of Clb2 exceeds a certain threshold (*J*_spn_) and is complete when [SPN]_n_ = 1 (Eq 50). We use a class-1 variable to describe the total concentration of Cdc20 (Eq 51) and then compute the concentration of Cdc20_A_ (unbound to the active form of Mad2) using a class-3 variable (Eq 52).

Cdc20_A_ binds to both the phosphorylated and unphosphorylated forms of APC. Because Cdc20_A_:APC is much less active than Cdc20_A_:APC_P_ in degrading Clb5 and Clb2, the degradation of Clb proteins at the end of the cycle is dependent on both the phosphorylation of APC and the release of the spindle assembly checkpoint. Both processes are promoted by Clb2. We calculate the concentrations of Cdc20_A_:APC_P_ and Cdc20_A_:APC by Eqs 53 and 54, which are alternative forms of a class-3 variable. We assume tight binding between Cdc20_A_ and APC_P_; so, the concentration of Cdc20_A_:APC_P_ is the concentration of either Cdc20_A_ or APC_P_, whichever is the lesser (Eq 53). Cdc20_A_ that is in excess of APC_P_ is assumed to bind tightly to APC (Eq 54). (We are assuming that Cdc20_A_ binds primarily to APC_P_ [65] and secondarily to unphosphorylated APC.)

#### Formulation of the Exit module

For a budding yeast cell to exit from mitosis and return to G_1_ it must transiently activate a phosphatase, Cdc14, which dephosphorylates many proteins that had been phosphorylated by cyclin-dependent kinases in S/G_2_/M. In particular, Cdc14 activates Cdh1 and CKI. These two factors stabilize G_1_ phase of the cell cycle by repressing the activity of Clb-dependent kinases. The activation of Cdc14, as described by the Exit module in Fig 8C, is accomplished by two pathways: FEAR (Cdc fourteen early anaphase release) and MEN (mitotic exit network) (for a review, see [70]). In the FEAR pathway, active Cdc20 cleaves Pds1 (securin) [66] and releases Esp1 (separase) [67]. Free Esp1 is responsible for chromatid separation [67] and inactivation of PPX (PP2A^Cdc55^), a phosphatase that dephosphorylates Net1 [71]. Subsequent phosphorylation of Net1 causes Cdc14 to be released from Net1:Cdc14 complexes in the nucleolus, and free Cdc14 is the phosphatase that drives exit from mitosis [71–75]. A schematic diagram of the FEAR pathway is: Cdc20 → Esp1—| PPX → Net1—| Cdc14.

We use a class-1 variable to describe the total concentration of Pds1 (Eq 55) and a class-3 variable to represent free Esp1, i.e., Esp1 that is not bound to Pds1 (Eq 56). The active forms of PPX and Net1 are described by class-2 variables (Eqs 57 and 59, respectively). The active (unphosphorylated) form of Net1 is a stoichiometric inhibitor of Cdc14, so we use a class-3 variable to represent free Cdc14 (Eq 61). In Eq 61 we introduce a stoichiometric factor, *ρ*_14,net1_, in order to simulate two mutants, *net1-ts* and *TAB6-1*, that show reduced association between Net1 and Cdc14 [74–76]. For wild-type cells we set *ρ*_14,net1_ = 1 and for *net1-ts* and *TAB6-1* cells we set *ρ*_14,net1_ < 1.

The FEAR pathway is not sufficient to return cells to G_1_ phase because Cdc14 activates Cdh1 which degrades Clb2, allowing Net1 to regain its ability to sequester Cdc14 in the nucleolus. To get robust phosphorylation of Net1 and full release of Cdc14 from the nucleolus, the FEAR pathway must be supplemented by the MEN pathway, whose role is to activate Cdc15 and Tem1 (see Fig 8C). Active Cdc15 then supports continued phosphorylation of Net1 even as Clb2-dependent kinase activity is dropping. Premature activation of Cdc15 is prevented by Clb2-dependent phosphorylation (inactivation) of Cdc15 in metaphase. Hence, Cdc15 activity is low as cells enter anaphase and rises abruptly as Clb2 is degraded by Cdc20_A_:APC_P_ and Cdc14 is activated by the FEAR pathway (Eqs 67 and 68) [77].

Meanwhile, Polo kinase, which was activated in prometaphase when Clb2-kinase activity was high (Eqs 62–64) [78], is able to activate Tem1 after PPX is inactivated by the FEAR pathway (Eqs 65 and 66) [71, 79]. Together, Tem1 and Cdc15 form an active complex (Tem1_A_:Cdc15_A_) (Eq 69) that phosphorylates Dbf2/Mob1 (not modeled explicitly), which then phosphorylates and inactivates Net1, resulting in full Cdc14 release. In this way, the transient release of Cdc14 by the FEAR pathway initiates a positive feedback loop between the activation of Tem1_A_:Cdc15_A_ (MEN) and further release of Cdc14 [42].

Full release of Cdc14 re-sets the cell to G_1_ by two complementary actions. First of all, Cdc14 dephosphorylates and activates Cdh1, and active Cdh1 (in association with APC) fully degrades Clb2 [68, 80, 81] and Polo kinase [82]. In addition, Cdc14 stabilizes CKI and activates its transcription factor, Swi5 [62, 83]. Abundant CKI and active Cdh1 are characteristic molecular signatures of G_1_ phase in budding yeast.

We assume that cell division occurs when the concentration of Clb2 drops below a threshold value, *K*_EZ_. Because budding yeast cells divide asymmetrically, we set the sizes of mother and daughter cells after division equal to 58% and 42% of the size of the dividing cell in glucose medium, which are close to the proportions observed by Di Talia *et al*. [36]. In galactose medium, for which cells grow more slowly and divide more asymmetrically [84], we take these proportions to be 61% and 39%.

BUD, ORI, and SPN, the dimensionless variables used to mark bud emergence, onset of DNA synthesis, and spindle assembly, are reset to 0 as the cell exits mitosis. We reset [BUD]_n_ and [SPN]_n_ to 0 at cell division, when [Clb2]_n_ = *K*_EZ_. We reset [ORI]_n_ to 0 when [Clb2]_n_+[Clb5]_n_ drops below *K*_EZ2_, because origins of replication are relicensed only if all Clb-dependent kinase activity is extinguished in G_1_ phase.

Full lists of initial conditions and kinetic constants used to simulate wild-type cells are given in Tables 3 and 5, respectively.

**Table 5:** Parameter values for wild-type cells.

**Rate constants** (min^−1^) (subscripts: “s” for synthesis, “d” for degradation)							
*k*_s,bck2_	0.00675	*k*_d,bck2_	0.5				
*k*_s,bud,e_	0.33	*k*_d,bud_	0.01				
*k*_s,cdc20_	0.006	*k*_s,cdc20,m1_	0.6	*k*_d,cdc20_	0.3		
*k*_s,cki_	0.024	*k*_s,cki,swi5_	0.24	*k*_d,cki_	0.005	*k*_d,ckip_	1.7
*k*_s,clb2_	0.0066	*k*_s,clb2,m1_	0.044				
*k*_d,clb2_	0.003	*k*_d,clb2,20_	0.14	*k*_d,clb2,20,i_	0.0366	*k*_d,clb2,h1_	0.6
*k*_s,clb5_	0.0006	*k*_s,clb5,bf_	0.029				
*k*_d,clb5_	0.012	*k*_d,clb5,20_	0.1	*k*_d,clb5,20,i_	0.015		
*k*_s,cln2_	0	*k*_s,cln2,bf_	0.27	*k*_d,cln2_	0.12		
*k*_s,cln3_	0.11	*k*_d,cln3_	0.4				
*k*_s,ori,e_	2	*k*_d,ori_	0.06				
*k*_s,pds1_	0.03	*k*_d,pds1_	0.05	*k*_d,pds1,20_	3	*k*_d,pds1,20,i_	0.3
*k*_s,polo,m1_	0.03	*k*_d,polo_	0.01	*k*_d,polo,h1_	0.1		
*k*_s,swi5_	0.005	*k*_s,swi5,m1_	0.08	*k*_d,swi5_	0.08		
*k*_s,spn_	0.1	*k*_d,spn_	0.06				
**Other time-scale factors** (min^−1^)							
*μ*	0.00693	(*mdt* = 100 min in glucose medium)					
*γ*	1	*γ*_cki_	2	*γ*_apc_	0.8	*γ*_tem1_	0.1
**Interaction coefficients** (dimensionless) (subscripts: “a” for activation, “i” for inactivation, “p” for phosphorylation, “dp” for dephosphorylation)							
*ω*_a,apc,b2_	0.65	*ω*_i,apc_	1				
*ω*_a,cdc15,14_	15	*ω*_i,cdc15_	1	*ω*_i,cdc15,b2_	0.25		
*ω*_a,cdh1_	1	*ω*_a,cdh1,14_	4.3	*ω*_i,cdh1,e_	1	*ω*_i,cdh1,n3_	1
*ω*_i,cdh1,n2_	0.6222	*ω*_i,cdh1,b5_	4.5	*ω*_i,cdh1,b2_	2.8		
*ω*_p,cki,n3_	4.2	*ω*_p,cki,k2_	0.4	*ω*_p,cki,n2_	0.3556	*ω*_p,cki,b5_	1.5
*ω*_p,cki,b2_	2.2	*ω*_dp,cki_	1	*ω*_dp,cki,14_	1.8		
*ω*_a,mad2_	20	*ω*_i,mad2_	0.4				
*ω*_a,mcm1,b2_	5	*ω*_i,mcm1_	3				
*ω*_p,net1,b2_	0.125	*ω*_p,net1,en_	1	*ω*_p,net1,15_	0.03		
*ω*_dp,net1_	0.1	*ω*_dp,net1,14_	0.1	*ω*_dp,net1,px_	3		
*ω*_a,polo,b2_	5	*ω*_i,polo_	1				
*ω*_a,ppx_	1	*ω*_i,ppx,pl_	3				
*ω*_p,sbf,b2_	2.5	*ω*_dp,sbf_	1				
*ω*_a,swi5,14_	2	*ω*_i,whi5,b2_	4.25				
*ω*_a,tem1,lo_	6	*ω*_i,tem1_	1	*ω*_i,tem1,px_	22		
*ω*_p,whi5,n2_	1.7778	*ω*_p,whi5,n3_	12	*ω*_p,whi5,k2_	16	*ω*_p,whi5,b5_	0
*ω*_dp,whi5_	1	*ω*_dp,whi5,14_	0.5				
**Total concentrations** (dimensionless)							
[APC_T_]_n_	25	[Cdc14_T_]_n_	2	[Cdc15_T_]_n_	1	[Cdh1_T_]_n_	1
[Esp1_T_]_n_	0.5	[Mcm1_T_]_n_	1	[Net1_T_]_n_	3.55	[PPX_T_]_n_	1
[SBF_T_]_n_	1	[Tem1_T_]_n_	2	[Whi5_T_]_n_	2.5	[Mad2_T_]_n_	25
**Other parameters** (dimensionless)							
*D*_cln3_	1	*e*_bud,b5_	0.25	*e*_bud,n2_	0.4444	*e*_bud,n3_	0.2
*e*_ori,b2_	0.35	*e*_ori,b5_	0.21	*J*_cln3_	6	*J*_spn_	0.26
*K*_EZ_	0.4	*K*_EZ2_	0.4	*ρ*_14,net1_	1		
*σ*	10	*σ*_net1_	8				
**Extra parameters used in stochastic simulations**							
*K*_flag_	0.8						
*k*_tr_	0.15 protein molecules per mRNA molecule per fL per min for all proteins						
*k*_dm_	0.7 min^–1^ for all mRNAs						
<*m*_min_>	5 mRNA molecules for *CLN2*, *CKI*, *CLB5*, *CLB2*, and *PDS1*, and 0 mRNA molecule for all other genes						

The full SCM (Table 4) tracks 33 protein species (not counting *V*, BUD, ORI and SPN) and requires ~100 parameter settings (Table 5). By comparison, Chen-2004 tracks the same number of protein species and requires ~120 parameter settings.

### 8. Deterministic simulations of wild-type and mutant cells

To simulate cell cycle progression in wild-type and mutant yeast cells, we solve the ODEs in Table 4 with the parameter values in Table 5. To simulate a broad selection of mutant yeast strains (S3 Table), we made appropriate changes to the “wild-type” parameter values, as outlined in S4 Table.

Our choice of wild-type parameter values (Table 5) was guided initially by the rate constant assignments in Chen *et al*. [41] and then adjusted manually to give a good fit to the observed phenotypes of the 133 mutant strains in S3 Table. We were able to account for the observed phenotypes of 125 of the 133 mutant strains (94%) in the data set we used to constrain the model. We tried automatic exploration of parameter space by a genetic algorithm (“Differential Evolution”) but could not find a set of parameter values that improves on the 94% success rate achieved manually [85].

**Fig 9:**
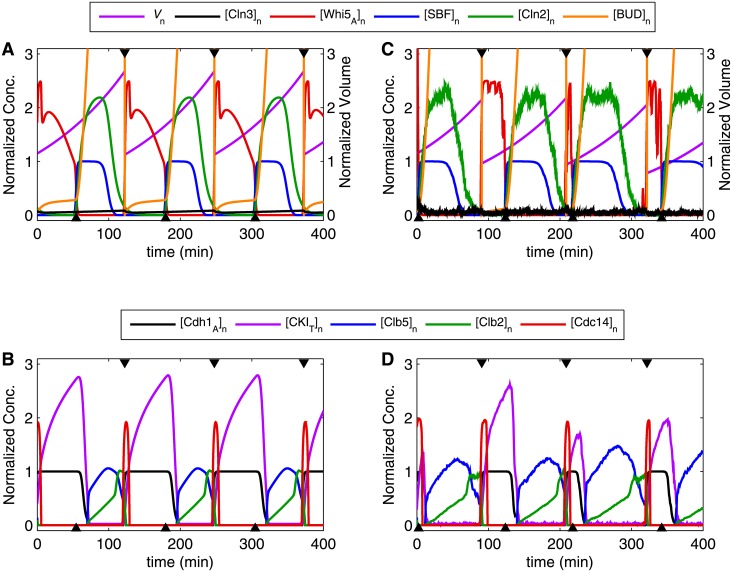
Simulations of wild-type cells. (A) Start: As the cell grows (increasing *V*_n_), Cln3 accumulates and phosphorylates Whi5. At a critical cell size, SBF is abruptly released from the inactive SBF:Whi5 complex and initiates a positive feedback loop between the accumulation of Cln2 and the phosphorylation of Whi5. (B) G_1_/S/G_2_/M: SBF also promotes the synthesis of Clb5. Once Clb5 titrates out CKI, then Clb5 and Cln2 together inactivate Cdh1, resulting in the accumulation of Clb2. Exit: Clb2 triggers many mitotic events, eventually leading to the release of Cdc14 during mitotic exit. When Clb2 drops below a normalized concentration of 0.4, the cell divides asymmetrically between daughter and mother cells. The daughter cell receives 42% of the cell size at division, and the mother cell (not shown here) receives the remaining 58%. (C and D) The stochastic model shows the typical fluctuations of protein concentrations around the average dynamics predicted by the corresponding deterministic model. For easier comparison to the deterministic simulation (A and B), we converted the numbers of molecules reported by the stochastic simulation to normalized concentrations. Start and division events are indicated by up-pointing and down-pointing black triangles, respectively.

The simulation of wild-type cells (Fig 9) shows oscillating patterns of cell cycle control variables that are in agreement with observations and expectations. The Start transition (Fig 9A) is initiated by the inactivation of Whi5 as Cln3 accumulates, followed by the positive feedback-driven expression of Cln2. Fig 9B shows an alternating pattern of G_1_ phase (during which G_1_ stabilizers CKI and Cdh1 are abundant) and S/G_2_/M phase (during which Clb5 and Clb2 are abundant). During mitotic exit, the release of Cdc14 resets the system back to G_1_ phase. Quantitative comparisons to experimental data will be discussed in the next section on stochastic simulations.

To verify our model, we computed the expected phenotypes of 133 mutant strains (S3 Table) and compared our results to the original model of Chen *et al*. [41] and to available experimental observations. (See our web site at http://mpf.biol.vt.edu for the observed phenotypes of mutant strains and references to the original literature.) Simulations that exhibit periodic cell division correspond to viable mutants, and simulations that arrest at some point in the cell cycle correspond to inviable mutants, with phenotypes assigned according to the rules in S5 Table. In our simulations, 125 mutants show phenotypic characteristics in agreement with the original model and experiments. (Notice that our SCM achieves the same accuracy as Chen-2004.) The eight mutant simulations that are not in agreement with observed phenotypes are listed in S6 Table and discussed in detail in S5 Text.

### 9. A stochastic SCM of the budding yeast cell cycle

In this section, we construct a stochastic version of our SCM, in order to address three questions. (1) Is our model of the cell cycle control system robust in the face of inevitable molecular fluctuations within single cells? (2) Is a stochastic version of our model consistent with quantitative measurements of cell cycle variability among wild-type cells? (3) Do stochastic models behave differently than deterministic models under some circumstances? With regard to the third question, we have in mind situations where mutations may make the regulatory mechanism less robust and more sensitive to molecular noise. For example, in the double mutant strain *CLB2*-*db*Δ *clb5*Δ (*CLB2* destruction box deletion and *CLB5* gene deletion) some cells are able to complete the cell cycle and divide whereas other cells become arrested in telophase and eventually die, as originally observed by Cross [86] and confirmed by Ball *et al*. [87]. This sort of partial viability is clearly incompatible with a deterministic model of the cell division cycle but may be accommodated by a stochastic model.

With regard to question (2), single-cell experimental techniques (e.g., flow cytometry and live-cell imaging of fluorescent proteins) have revealed much information about cell-to-cell variation among genetically identical cells. In budding yeast, fluorescence microscopy has been used to study proteins that regulate cell cycle progression and to determine the onset of cell cycle events such as Start, bud emergence, and cell division in individual cells [38, 88, 89]. In addition, fluorescence in situ hybridization (FISH) has been used recently to quantify mRNA levels in individual yeast cells [90], determining that a yeast cell carries roughly 5–15 mRNA molecules of each gene measured, including a sample of cell cycle control genes [91]. Since the number of mRNA molecules directly determines the production rate of its protein product, noise due to low, fluctuating numbers of mRNA molecules will be significantly amplified as fluctuations in protein abundance [39]. Consistent with this expectation, stochastic simulations by Kar et al. [92] suggested that mRNA noise is a major source of fluctuations in the budding yeast cell cycle control system. Therefore, to understand stochasticity in the system, inclusion of mRNA noise seems to be necessary. The stochastic version of SCM described above was designed specifically to deal with molecular noise derived from both protein and mRNA fluctuations.

#### Model conversion

In our deterministic model (Eqs 21–69) that we reformulated from Chen-2004, the variables ([Cln2]_n_, [Clb2]_n_, etc.) represent concentrations in “normalized” units (i.e., scaled with respect to a characteristic concentration of each component). Stochastic studies, on the other hand, measure protein abundances in terms of numbers of molecules. Before constructing the stochastic model, we first convert the normalized concentration units used in the deterministic SCM into numbers of molecules using the conversion process described in [93]. The details of the model conversion are discussed in S6 Text.

#### The stochastic model

After converting “normalized concentrations” to “numbers of molecules” we add molecular noise to class-1 variables by the Langevin formalism, as described above. For example, the stochastic rate equation for the total number of Clb5 molecules is:

$$\begin{array}{l} \frac{\text{d}Clb5_{\text{T}}}{\text{d}t}=A_{\text{clb}5}-B_{\text{clb}5}\cdot \text{Clb}5_{\text{T}}\\ \;+\sqrt{A_{\text{clb}5}+B_{\text{clb}5}\cdot Clb5_{\text{T}}}\cdot \frac{\zeta_{1}(t)}{\sqrt{\Delta t}}\\ \;+Clb5_{\text{T}}\sqrt{2B_{\text{clb}5}\frac{k_{\text{tr}}\cdot V_{\text{fL}}}{A_{\text{clb}5}+\langle m_{\text{min}}\rangle \cdot k_{\text{tr}}\cdot V_{\text{fL}}}\cdot \frac{B_{\text{clb}5}}{B_{\text{clb}5}+k_{\text{dm}}}}\cdot \frac{\zeta_{2}(t)}{\sqrt{\Delta t}},\\ \text{where}\;\;\;A_{\text{clb}5}=\varphi \cdot c_{\text{clb}5}\cdot k_{\text{s},\text{clb}5}+\frac{c_{\text{clb}5}}{c_{\text{sbf}}}\cdot k_{\text{s},\text{clb}5,\text{bf}}\cdot SBF+\mu \cdot Clb5_{\text{T}}\\ \text{and}\;\;\;B_{\text{clb}5}=k_{\text{d},\text{clb}5}+\frac{k_{\text{d},\text{clb}5,20}}{\varphi \cdot c_{\text{cdc}20}}\cdot Cdc20_{\text{A}}:APC_{\text{P}}+\frac{k_{\text{d},\text{clb}5,20,\text{i}}}{\varphi \cdot c_{\text{cdc}20}}\cdot Cdc20_{\text{A}}:APC, \end{array}$$ (70)

where *A*_clb5_ and *B*_clb5_ are the converted versions of the synthesis and degradation rates from the deterministic model (see S6 Text for details). The mRNA-inherited noise term in Eq 70 is slightly different from the term in Eq 19 because our full model does not account explicitly for the mRNA species (see S7 Text for the derivation of Eq 70). *k*_tr_ is the protein translation rate (the number of protein molecules produced per mRNA molecule per fL per min). *V*_fL_ = *V*_n_·*c*_vol_ is cell volume in fL, where *c*_vol_ is average cell size at birth of wild-type daughter cells (28 fL). ⟨*m*_min_⟩ is the minimum number of mRNA molecules of each gene always present in the cell. *k*_dm_ is the mRNA degradation rate. We treat *k*_tr_, ⟨*m*_min_⟩, and *k*_dm_ as tuning parameters. (See S8 Text for the discussion of the choices of ⟨*m*_min,*i*_⟩ in our model.) *ζ*_1_ (*t*) and *ζ*_2_ (*t*) are independent random variables chosen from a normal distribution N(0, 1) with mean = 0 and standard deviation = 1. A term, *μ*·*Clb5*_T_, is added the production rate to make for consistency between the deterministic and stochastic models (see discussion in S6 Text). *c*_clb5_, *c*_sbf_, and *c*_cdc20_ are the characteristic concentrations (in nmol/L) for the indicated proteins (listed in Table 3), and *c*_S_ ∙ *V*_fL_ ∙ *N*_A_ ∙ 10^−9^ ∙ 10^−15^ = *c*_S_ ∙ *φ* is the characteristic number of molecules of species S in volume *V*_fL_, where *φ* = 0.6 *V*_fL_.

In our deterministic model, the cell divides whenever the normalized concentration of Clb2 drops below a threshold *K*_EZ_. In the stochastic model, fluctuations around the threshold could cause multiple division events. To avoid this problem, we add an extra condition to the rule for cell division in the stochastic model: (1) a cell divides when [Clb2]_n_ drops below the threshold *K*_EZ_, and (2) the cell cannot divide again until [Clb2]_n_ increases above a higher threshold *K*_flag_ (*K*_flag_ *> K*_EZ_).

We do not add molecular noise to variables of classes 2 and 3, because we assume they change on much faster time scales than class-1 variables and therefore regress quickly to their mean values.

In addition to molecular noise from protein and mRNA fluctuations, budding yeast cells are also subject to variations in the cell division process itself. In our simulations, the daughter cell receives, on average, 42% of the volume and constituent proteins of the dividing cell, with a standard deviation of 5%.

The values of the extra parameters used in our stochastic simulations are listed in Table 5.

### 10. Stochastic simulations of wild-type and mutant cells

Stochastic simulations of wild-type cells (e.g., Fig 9C and 9D) show high variability in progression through the cell cycle. Following the lead of Cross and his group [38], we divide the cell cycle into three periods: *T*_1_, *T*_2_, and *T*_b_. *T*_1_ is the duration from cell birth to Start, which we identify with SBF reaching 50% of its maximum value. *T*_2_ is the period from Start to bud emergence, which we identify with [BUD]_n_ = 1. Finally, *T*_b_ is the duration of the “budded phase,” from bud emergence to the next cell division.

**Fig 10:**
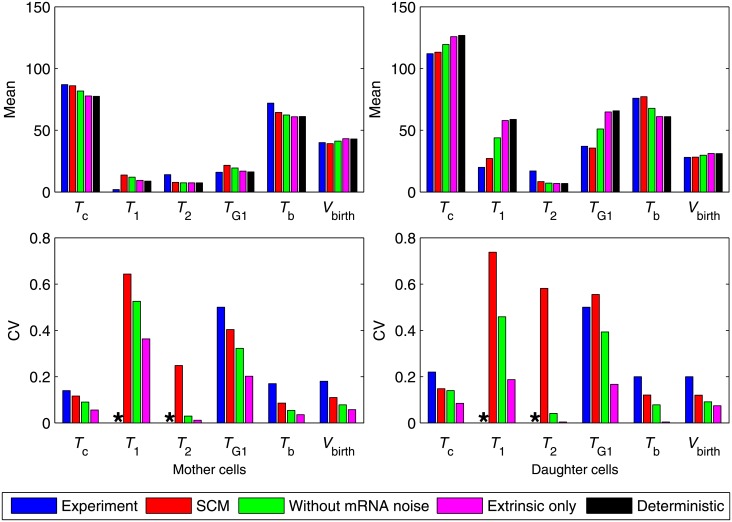
Statistical properties of cell cycle progression. Experimental observations for wild-type cells [38] (blue bars) are compared to simulations from four different cases of our model: the full stochastic SCM (red bars), the stochastic SCM without the mRNA-inherited noise term (green bars), the deterministic SCM with “extrinsic” noise only (magenta bars), and the completely deterministic SCM (black bars). *T*_c_ is cell cycle duration (min) = *T*_G1_ + *T*_b_ = *T*_1_ + *T*_2_ + *T*_b_. *T*_1_ is the duration from cell birth to Start (min), which we identify with SBF reaching 50% of its maximum value. *T*_2_ is the period from Start to bud emergence (min), which we identify with [BUD]_n_ = 1. *T*_G1_ = *T*_1_ + *T*_2_ = duration of the “unbudded phase” (min). *T*_b_ is the duration of the “budded phase,” from bud emergence to the next cell division (min). *V*_birth_ is cell size at birth (fL). Asterisks indicate unreported data.

From stochastic simulations of wild-type cells, we computed means and variances of *T*_1_, *T*_2_, *T*_b_, cell cycle duration, and cell size at birth, which we compare to experimental observations [38] in Fig 10. (The full distributions of *T*_1_, cell cycle duration and *V*_birth_, for both mother and daughter cells, are shown in S7 Fig.) Overall, our results show a reasonable agreement with the experimental data, although there are some quantitative discrepancies. Our simulated *T*_1_ is longer and our simulated *T*_2_ is shorter than observed in both mother and daughter cells, which may be attributed to a difference between our theoretical criterion for Start (SBF reaching 50% of its maximum activity) and the experimental criterion for Start (Whi5 disappearance from the nucleus). *T*_1_ + *T*_2_ = *T*_G1_ = duration of the “unbudded phase” is longer than observed in mother cells, but quite accurate for daughter cells. The predicted and observed CVs of *T*_G1_ are all ~50%. In our simulations the average delay from bud emergence (when [BUD]_n_ = 1) to the initiation of DNA synthesis (when [ORI]_n_ = 1) is ~12 min, which is inconsistent with the observation that the two events occur nearly simultaneously in wild-type cells.

#### Fluctuations in both mRNA and protein are needed to account for the variability observed in wild-type cells

In Fig 10, we decompose the effects of noise in our model by comparing four different cases: the full stochastic SCM, the stochastic SCM without the mRNA-inherited noise term in Eq 70, the deterministic SCM with “extrinsic” noise only (retain 5% variation in the cell division process, which is one component of extrinsic noise, but no intrinsic noise terms in Eq 70), and the completely deterministic SCM. (The full distributions for the first three cases are presented in S7 Fig). The full stochastic model with protein and mRNA fluctuations clearly shows the highest variations, which are comparable to the experimental values. Interestingly, noise contributes significantly to the behavior of wild-type daughter cells before Start, because the full stochastic simulation predicts an average *T*_1_ duration (27 min) that is markedly shorter than the result from the model without mRNA fluctuations (44 min), the extrinsic-only model (58 min), and the deterministic model (*T*_1_ = 59 min, no variability). In our model, the major source of intrinsic noise comes from fluctuations of Cln3 and Bck2, because the average total amount of Cln3 is quite small (~100 molecules) [31] and we assume that Bck2 has a similar abundance. Since the Start transition is driven by a bistable switch [36], high fluctuations of Cln3 and Bck2 combined with the positive feedback loop engaged by Cln2 can result in *T*_1_ duration that is much shorter than what is predicted by the deterministic system. This shortening of *T*_1_ is a genuine divergence of the stochastic model from the deterministic model. In the deterministic model, the cell cannot execute Start until it surpasses the saddle-node bifurcation point (Fig 2) where the pre-Start steady state disappears. In the stochastic model, however, molecular fluctuations can induce premature transitions from the “excitable” pre-Start steady state to the much more stable post-Start steady state before the system reaches the saddle-node bifurcation point [94].

#### The duration of G_1_ is negatively correlated with birth size in experiment and in simulation

**Fig 11:**
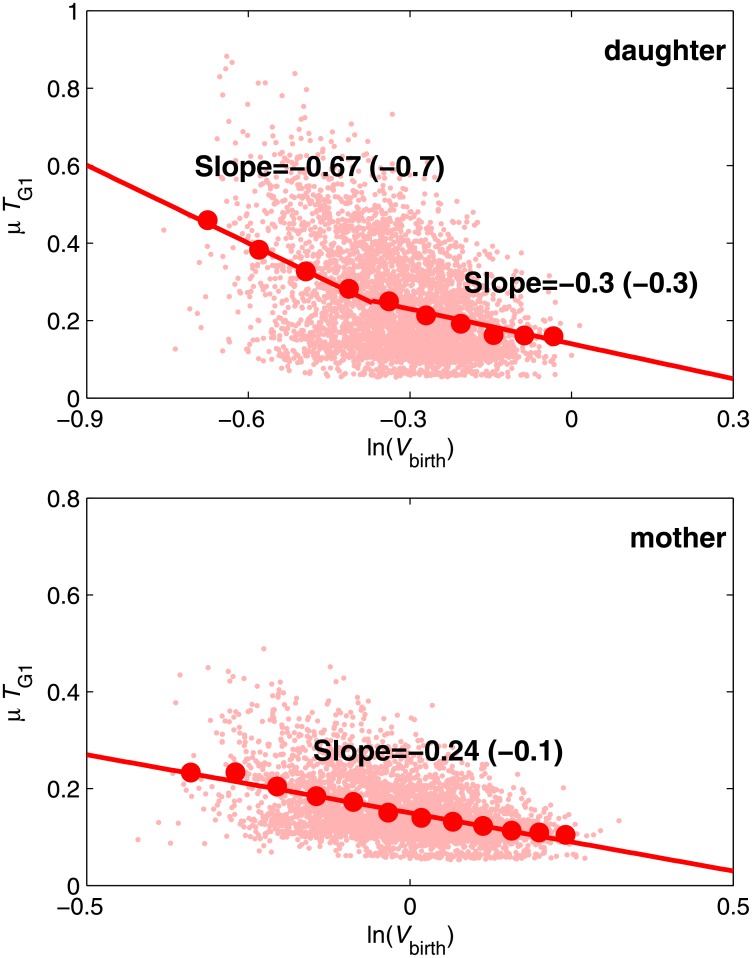
The joint distributions of cell size at birth and G_1_ phase duration predicted by our stochastic model of the full cell cycle control system. (Upper panel) Daughter cells. (Lower panel) Mother cells. *T*_G1_ is the duration from cell birth to bud emergence, and *μ* is the specific growth rate of the cell culture. In the plots, cell size is normalized by the average size of mother cells at birth and is plotted on a log scale. Small dots in the background represent data collected from simulations of individual cells. Large dots represent average *μ*·*T*_G1_ of the small dots binned in 2 fL intervals. For daughter cells, the binned data can be divided into two groups (small and large cells; break point at ln(*V*_birth_) = −0.37) and fitted by two straight lines with slopes of –0.67 and –0.30 (respectively). Mother cells can be fitted by one straight line with slope –0.24. The experimental slope values, as reported by Di Talia *et al*. [38], are shown in parentheses. The binned data for mother cells is slightly better fit by two straight lines with a break point at ln(*V*_birth_) = 0.

Next, we study the correlation between size at birth and duration of G_1_ phase (*T*_G1_). If cell cycle progression during G_1_ is perfectly controlled by cell size, then a plot of *μ T*_G1_ against ln(*V*_birth_) should give a straight line with slope = –1 [38], where *μ* is the specific growth rate and *V*_birth_ is the birth size of cells. In the experiment of Di Talia *et al*. [38], small daughter cells show the expected negative correlation (slope = –0.7), indicating a strong size-control mechanism operating in small budding yeast cells. Larger daughter cells show less negative correlation (slope = –0.3). Mother cells seem to operate with little or no size control (slope = –0.1). In good accord with experimental observations, our simulations (Fig 11) show that the small and large daughter cells exhibit correlations with slopes –0.67 and –0.30, respectively. The break between strong size control and weak size control occurs at a birth size of $\sim 0.69\times {\bar{V}}_{\text{m}}$, where ${\bar{V}}_{\text{m}}$ = average size of mother cell at birth. The mother cells show less correlation (slope = –0.24).

It is beyond the scope of this paper to provide a statistically rigorous analysis of the negative correlation between G_1_ duration and size at birth predicted by the SCM. In a separate study, we have fitted our simulations to the full experimental distributions (kindly provided by Stefano Di Talia). We subdivided the empirical joint distribution into 17 rectangles (“bins”) so that no bin contained more than about 20 cells or less than 3, and we subdivided the simulated joint distribution in an identical fashion. Then we computed the Hellinger distance between the two distributions (empirical and simulated) and minimized the distance by a quasi-Newton algorithm for stochastic optimization [95]. By using the full distribution (rather than the two-slope representation) we were able to improve the fit of the model to the data, but there remained a noticeable discrepancy in that the model predicts longer tails of G_1_ durations than are observed experimentally for both mother and daughter cells. Hence, although the model is qualitatively in accord with the observed negative correlation between *T*_G1_ and birth size, it exhibits long tails in the *T*_G1_ distributions that appear to be statistically significant deviations from observed G_1_ durations. These results will be reported in full in a later publication.

In S8 Fig we plot the correlations of cell size at birth with both *T*_G1_ and *T*_1_ and confirm the experimental observation [38] that *T*_2_ = *T*_G1_ –*T*_1_ is relatively constant. This observation indicates that the size control mechanism in budding yeast operates at the Start transition, i.e., at the phosphorylation and inactivation of Whi5. Whi5 inactivation frees up SBF to drive Cln2 and Clb5 production. The initial phosphorylation of Whi5 is the job of Cln3 and Bck2, and since their rates of accumulation are proportional to cell size, *V*, small cells take a longer time to inactivate Whi5, and large cells take a shorter time.

#### Simulated numbers of protein molecules for an asynchronous population are in agreement with observations

Next, we study the abundances of protein species in an asynchronous population, as might be measured experimentally. To this end, for each simulated cell we select a random time point between birth and division and record the number of each protein species present at that time. To obtain a sample of time points representative of an asynchronous population of cells, we must take into account that the number of newborn cells is twice the number of dividing cells. Hence, we select a random number *θ*, 0 ≤ *θ* ≤ 1, from an exponential probability density function

$$f(\theta )=\text{ln}(2)\cdot 2^{1-\theta }$$ (71)

and then calculate *t*_birth_ + (*t*_division_−*t*_birth_)·*θ* as a random time point in each complete cell division cycle. By this prescription, the probability of choosing a cell at birth is twice the probability of choosing a cell at division. (Eq 71 is not exactly correct for asymmetrically dividing cells, like budding yeast, but the errors introduced by this approximation are minor.)

This set of simulated protein abundances should be comparable to numerical values collected from an asynchronous population of yeast cells. In Table 6 we show that our computed average protein abundances are in good agreement with experimental values [31, 96], except for Clb5 and Clb2, whose predicted abundances are two-fold larger and smaller, respectively, than observed.

**Table 6:** Average protein abundances.

Protein name	Simulation (per haploid cell)	Experiment [96] (per haploid cell)	Experiment [31] (per diploid cell)
Cdc15	249	238	-
CKI (Sic1+Cdc6)	626	768*	214*
Clb1+Clb2	382	693	1,625
Clb5+Clb6	982	-	876
Cln1+Cln2	1,511	1,589	3,006
Cln3	83	-	216
Net1	1,991	1,590	-
PPX	3,116	3,170	-
Tem1	499	573	-
Whi5	1,714	1,440	-

*only Sic1 data is available.

#### Stochastic simulations of the mutant strain *CLB2-dbΔ clb5Δ* explain its partial viability when growing on raffinose medium

Next we study viability of the mutant strain *CLB2*-*db*Δ *clb5*Δ. In this strain, Clb2’s destruction box has been deleted from the *CLB2* gene, so Clb2 protein cannot be degraded by Cdc20 and only partially so by Cdh1 [86]. This mutant strain is inviable on glucose medium (mass-doubling time = *mdt* = 100 min) but can be partially rescued on raffinose medium, which supports slower growth (*mdt* = 150 min) [86, 87]. Obviously, “partial viability” (some cells complete the cell cycle and some do not) cannot be explained by a deterministic model in which all cells behave the same. A stochastic version of Chen's 2004 model [87] predicts that some mutant cells, growing in raffinose, divide properly while others fail to exit from mitosis. However, the model in [87] applied Gillespie's SSA directly to Chen's 2004 model without unpacking the complex rate laws into elementary steps, and it did not take into account transcription-translation coupling. Hence, the results of the model in [87], though suggestive, are not very reliable. It is challenging to test whether our SCM approach provides a better account of the properties of this mutant strain.

**Fig 12:**
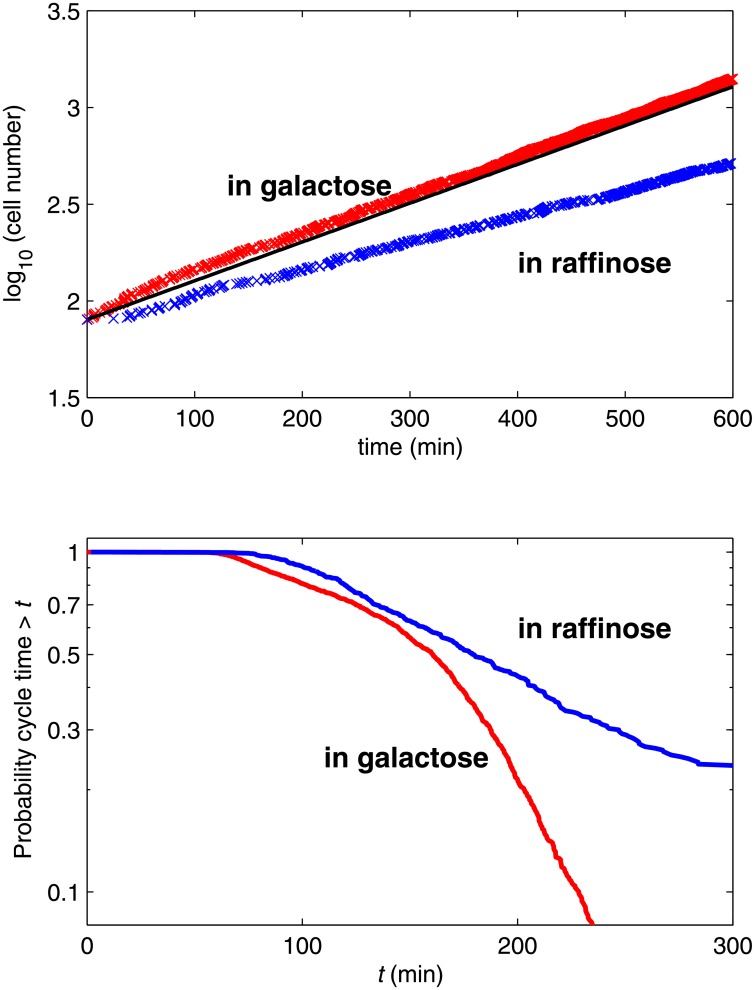
Growth curves and cycle time distributions for *CLB2*-*db*Δ *clb5*Δ *GAL-SIC1* cells. (Upper panel) Logarithm of the total number of cells is plotted against time. The increase in cell number for *CLB2*-*db*Δ *clb5*Δ *GAL-SIC1* cells growing in galactose (red crosses; Sic1 overexpressed) indicates exponential growth (black line) with the number doubling time = 150 min. The number doubling time of the same cells growing in raffinose (blue crosses; normal level of Sic1) is greater than 150 min, indicating that some cells do not complete the cell cycle when *GAL-SIC1* is not expressed. (In our simulations, the mutant cells are assumed to have mass doubling time = 150 min in both galactose and raffinose media.) (Lower panel) Cumulative distributions of cycle times for *CLB2*-*db*Δ *clb5*Δ *GAL-SIC1* cells growing in galactose medium (red line) or in raffinose medium (blue line). The ordinate, *P*(*t*), is the probability that a simulated cell has a cycle time greater than *t*.

Since the mutant cells cannot survive on glucose medium and are only partially viable on raffinose, the mutant strain is kept viable in the laboratory by introducing the *GAL-SIC1* gene into the *CLB2*-*db*Δ *clb5*Δ cells and growing the strain in galactose, where Sic1 is overexpressed. In Fig 12, top panel, we show predicted growth curves for *CLB2*-*db*Δ *clb5*Δ *GAL-SIC1* cells grown in media containing galactose (*GAL-SIC1* is “on”) or raffinose (*GAL-SIC1* is “off”). These cells proliferate in galactose with a population number-doubling time (*ndt*) ≈ 150 min, which is equal to the mass-doubling time (*mdt*) of individual cells. On the other hand, for the triple mutant cells growing in raffinose, *ndt* > 150 min, indicating that some cells are inviable. Cell viability is better illustrated in Fig 12, bottom panel, where we plot cumulative distribution functions for triple-mutant cells in galactose and in raffinose. Greater than 90% of the cells growing in galactose (*mdt* = 150 min and Sic1 overexpressed) complete the cell cycle within 250 min of birth, indicating that they are mostly viable. On the other hand, for the same cells growing in raffinose (*mdt* = 150 min but Sic1 not overexpressed), ~ 25% of the cells are undivided even 300 min after birth, indicating that ~25% of the cells growing in raffinose never complete the cell division cycle. This result agrees well with the experimental observation [87] that ~15–40% of these mutant cells never divide in raffinose growth medium. Furthermore, our simulations of mutant cells grown in glucose medium (*mdt* = 100 min and Sic1 not overexpressed) show less than 10% viability, which is consistent with the fact that these cells are inviable in glucose medium [86]. In Table 7, we compare the statistical properties of this mutant strain grown in raffinose from simulations and from experimental measurements [87].

**Table 7:** Statistical properties of simulated *CLB2*-*db*Δ *clb5*Δ cells in raffinose. Mean cycle time (in minutes) with standard deviation in parentheses.

	Our Simulation	Experiments [87]*			Simulation in [87]
		1	2	3	
Mother cells	150 (50)	151 (65)	165 (63)	145 (80)	142 (23)
Daughter cells	155 (52)	151 (63)	164 (53)	143 (82)	152 (27)

*Authors in [87] used the time between successive budding events to represent the cycle time.

As in the original model of Chen *et al*. [41], we assume that the synthesis of Clb2 is dependent on cell size. By this assumption, cell size at the onset of mitotic exit is critical for the cell's fate. Cells with larger size at the onset of mitotic exit have more Clb2 and are less likely to exit from mitosis. (Cells exit only if Clb2 is degraded below a threshold concentration.) In wild-type cells, the presence of both Cdc20 and Cdh1 ensures that Clb2 is fully degraded during mitotic exit. However, Clb2 protein encoded by the *CLB2*-*db*Δ gene lacks the sequence targeted by Cdc20 and can be only partially degraded by Cdh1. To exit from mitosis, *CLB2*-*db*Δ cells depend critically on the activation of Cdh1 and Sic1, but both Cdh1 and Sic1 are also inhibited by Clb2-dependent kinase. When grown on a rich medium such as glucose, the mutant strain has a high growth rate and Clb2 accumulates to such a high level that it keeps Cdh1 and Sic1 inactive, resulting in telophase arrest. On a slower growth medium such as raffinose or galactose, a cell may exit from mitosis if Cdh1 and Sic1 can suppress Clb2. This explains why cells show high viability when Sic1 is overexpressed in galactose. In raffinose, however, without the help of Sic1 overexpression, noise plays a major role in determining whether or not a cell can exit from mitosis and divide. Cells with higher activities of Sic1 and/or Cdh1 or lower levels of Clb2 may be able to divide, but cells at the other extreme will be arrested in anaphase.

In S9 Fig we show that *CLB2*-*db*Δ *GAL-SIC1* (*CLB5* in place) mutant cells growing in raffinose (Sic1 not produced) have comparable viability to *CLB2*-*db*Δ *clb5*Δ *GAL-SIC1* cells growing in raffinose, because Clb5 protein is mostly degraded by APC/Cdc20 by the time the *CLB2*-*db*Δ *CLB5* cells are exiting (or not) from mitosis. Although *CLB2*-*db*Δ *CLB5 GAL-SIC1* cells are inviable in glucose medium (rapidly growing cells that are not producing Sic1), there is anecdotal evidence that these cells are partially viable when cultured on poor growth medium, like raffinose [86]. So, our stochastic model is consistent with a conclusion that *CLB2*-*db*Δ *CLB5* cells are on the cusp of viability/inviability when growing on poor carbon sources.

## Discussion

We have presented a “standard component modeling” strategy for protein regulatory networks by grouping proteins into three classes according to the presumptive time scales of the reactions involved. We assume that class-1 proteins change slowly in time due to synthesis and degradation, and we describe these changes by pseudo-linear ODEs. With good estimates for the rate constants of synthesis and degradation, the dynamics of class-1 variables can be compared directly to experimental data (i.e., time-series data). Class-2 proteins change more rapidly in time due to protein modifications and are described by nonlinear ODEs employing the soft-Heaviside function, *H*(*x*) = 1/(1+*e*^−*x*^). The soft-Heaviside function gives us many of the advantages of hybrid Boolean-ODE models (e.g., the piecewise linear ODE models of Leon Glass and coworkers [8]) while retaining the powerful analytical tools available for nonlinear ODEs (e.g., bifurcation theory). Class-3 variables describe strongly bound protein complexes that form rapidly from two subunits; hence, the total amounts of the bound and free forms can be easily computed using the “max” function.

The SCM approach has many advantages. Its basic assumptions are natural because these three classes of protein species are commonly encountered in PRNs. The modularity of the components allows modelers to build mathematical representations of complicated networks in a controlled and logical fashion. Modularity also renders the models easily modifiable and extensible. Because the models are rendered in terms of nonlinear ODEs, the modeler has access to well-tested software for simulation, analysis (bifurcation theory, sensitivity analysis), and parameter estimation. Furthermore, the SCM formalism is readily adapted to stochastic simulation by chemical Langevin equations (CLEs), and we have shown how to incorporate transcription-translation coupling into the CLEs to account for fluctuations at the mRNA level without necessarily having explicit mRNA species in the model.

The vulnerabilities of the approach should also be clearly recognized. Although the soft-Heaviside function conveniently represents a sigmoidal signal-response curve, it has no basis in biochemical reaction mechanisms, and the interaction coefficients (*ω*_*ji*_’s) are not related to any measurable reaction rate constants. The use of the “max” function to describe class-3 variables is even more restrictive. Strictly speaking it applies only to strongly bound dimeric complexes that form rapidly and reversibly. It can be extended to other special cases, but it is easy to imagine realistic situations in which the max-function approximation gives unsatisfactory results.

Nonetheless, the modularity of an SCM allows problematic situations to be spotted and readily repaired. For example, if protein components in a mechanism form competing binary and ternary (and higher) complexes, the max functions proposed here can be replaced by a set of nonlinear algebraic equations (the equilibrium binding equations) for the amounts of the complexes. In this case, the SCM becomes a system of differential-algebraic equations (DAEs), and there are well-tested numerical algorithms (e.g., DASSL [97]) for solving DAEs accurately and efficiently. Alternately, one could replace the equilibrium binding equations by ODEs for the complexes themselves, and solve the larger system of nonlinear ODEs by a suitable “stiff” ODE solver. It may also be advisable in some circumstances to replace the soft-Heaviside function, governing class-2 variables, by a more realistic (biochemically based) nonlinearity, e.g., a Hill function for transcription factor-binding to gene promoters. The modularity of SCMs makes such replacements easy.

Indeed, we do not want to give the impression that SCM is a take-it-or-leave-it approach. It would be quite reasonable for parts of a model to be SCM-like and other parts more biochemically realistic. A model may start life as a Boolean network capturing the gross qualitative features of a physiological trait, be translated into an SCM to provide more quantitative details for comparison to experiments, and later get fleshed out with full biochemical verisimilitude. Alternatively, we may wish to extend a biochemically detailed model, like Chen-2004 for the budding yeast cell cycle, to new aspects of the control system. Mechanistic proposals for these new aspects can be grafted on to the full model using the SCM approach for a quick appraisal. If biochemical details are lacking, the new parts of the model will coexist quite peacefully with the original ODE model. If the biochemistry is known and relevant, the SCM modules can be swapped out for something more realistic.

We have applied this approach to a detailed molecular mechanism of cell cycle control in budding yeast. Compared to a model based on traditional biochemical kinetics [41], our new model based on “standard components” has fewer parameters that need to be estimated from experimental data. Furthermore, in our experience, the standard component model (SCM) is easier to build, easier to modify and extend, and easier to parameterize by fitting the model to experimental data. Nonetheless, the SCM is just as accurate as the detailed biochemical model [41], reproducing the phenotypes of 94% of the mutant budding yeast strains in our test collection. A major advantage of the SCM is that it can easily be converted to a stochastic model that can account for cell-to-cell variability in wild-type and mutant strains of budding yeast. The stochastic SCM accounts for the impact of mRNA fluctuations on protein fluctuations, without requiring explicit modeling of mRNA dynamics. Because the stochastic SCM is formulated in terms of stochastic differential equations of the Langevin type, it can be simulated very efficiently compared to Gillespie’s “stochastic simulation algorithm” [14] applied to a fully detailed biochemical kinetic model.

Although the SCM approach has proved successful in describing cell cycle regulation by cyclin-dependent kinases, its potential for describing other PRNs must await future attempts to apply the approach to other problems (e.g., for circadian rhythms, epidermal growth factor signaling, epithelial-to-mesenchymal cell transition).

## Methods

See S9 Text for our simulation methods. All simulations (both deterministic and stochastic) were done by Euler’s explicit, first-order method with step size Δ*t* = 0.01 min. In S10 Text we provide evidence that this step size is small enough to obtain reliable results.

## Supporting Information

S1 Fig The soft-Heaviside function. *H*(*σW*) = 1/1(1 + *e*^−*σW*^) varies smoothly from 0 to 1 as a function of increasing *W*. The parameter *σ* determines the steepness of the function. The soft-Heaviside function approaches the true Heaviside function as *σ* **→** ∞.
(EPS)

S2 Fig Two-parameter bifurcation diagrams for the Start models. Upper panel: The two-parameter bifurcation diagrams show similar regions of bistability between the two models when the synthesis rate of Cln3 (*k*_s,n3_) and (fixed) cell volume are varied. Lower panel: However, at large synthesis rates of ClbS (*k*_s,bS_), the values of (fixed) cell volume that exhibit bistability are different between the two models. The value of *k*_s,bS_ used in both models is 0.3 fL^−1^ min^−1^ (taken from the value used by Barik *et al*. [27]). At this value, both models show a similar bistability region when (fixed) cell volume is varied.
(PDF)

S3 Fig *T*_2_ durations of cells with different initial cell size (*V*_0_) from the Start models. In both models, the gap between Start and the G_1_/S transition, *T*_2_ = *T*_G1_ –*T*_1_, is nearly independent of birth size.
(EPS)

S4 Fig Steady-state distributions of Cln3, SBF and ClbS in Start models at fixed cell size. Black lines: MultiP model in S1 Text; red lines: stochastic SCM model in S3 Text; blue lines: stochastic SCM without mRNA noise (i.e., all *ζ*_2_(*t*) = 0 in S3 Text); green lines: stochastic SCM with explicit mRNA species, as described in S4 Text. The steady-state distributions at fixed *V* are shown as the Kernal density estimation. *V* = 10 fL is a very small yeast cell, *V* = 35 fL corresponds to cells that have executed the Start transition. At large *V*, the distributions from the SCM without mRNA noise are much narrower than the distributions from the other models. Means of SBF molecules from the three SCM models deviate from the SBF mean from the MultiP model (panel C).
(EPS)

S5 Fig Stochastic simulations of the relation between initial cell size (*V*_0_) and average cell age at the Start transition (*T*_1_) (upper panel) and average cell age at the G_1_/S transition (*T*_G1_) (lower panel) for the Start models. The average values of *T*_1_ and *T*_G1_ agree well between the models and show similar patterns to the deterministic simulations in Fig 4 of the main text.
(EPS)

S6 Fig Distributions of cell age at the Start transition (*T*_1_) and at the G_1_/S transition (*T*_G1_) from the Start models. Distributions of *T*_1_ and *T*_G1_ with initial cell size (*V*_0_) of 10, 35, and 50 fL from Figs 6 and 7 of the main text are shown as the Kernal density estimation. Similar to what we found in Figs 6 and 7, the distributions from the SCM (red) and the MultiP model (black) agree well at small *V*_0_ (*e*.*g*., *V*_0_ = 10 fL). The SCM without mRNA noise (blue) always exhibits narrower deviation than the other models. The SCM with explicit mRNA species (green) shows better agreement to the MultiP model than the SCM and the SCM without mRNA noise at large *V*_0_ (*e*.*g*., *V*_0_ = 35 and 50 fL).
(EPS)

S7 Fig Distributions of cell cycle properties from the full cell cycle SCM. Distributions of some cell cycle properties from Fig 10 of the main text: cell cycle time (*T*_c_), time from cell birth to Start (*T*_1_), and volume at cell birth (*V*_birth_), are shown as the Kernal density estimation. *T*_1_ duration predicted from the full stochastic simulation (Panel D, black) is markedly shorter than the results from the model without mRNA fluctuations (Panel D, red) and the extrinsic-only model (Panel D, blue) (see main text for discussion).
(EPS)

S8 Fig Average values of G_1_ phase (*T*_G1_) and Start phase (*T*_1_) durations of daughter cells calculated from data collected from simulations of individual cells binned in 2 fL volume intervals from our stochastic simulations of the full cell cycle model. Red dots: *T*_G1_ from Fig 11 (main text); blue dots: *T*_1_, the time from cell birth to SBF reaching 50% of its maximal activity. The gap between the two lines confirms the experimental observation that *T*_2_ = *T*_G1_ –*T*_1_ is relatively constant.
(EPS)

S9 Fig Cycle time distributions for *CLB2*-*db*Δ *GAL-SIC1* cells (*CLB5* in place) in raffinose medium. Cumulative distributions of cycle times for *CLB2*-*db*Δ *GAL-SIC1* cells (dashed line) and *CLB2*-*db*Δ *clb5*Δ *GAL-SIC1* cells (solid line; from Fig 12 of the main text) growing in raffinose medium (*GAL-SIC1* is “off”). The ordinate, *P*(*t*), is the probability that a simulated cell has a cycle time greater than *t*. Our stochastic model is consistent with Cross’s suggestion that the inviability of *CLB2*-*db*Δ *GAL-SIC1* cells growing on glucose medium is partially rescued by growth on alternative poor-carbon sources (e.g., raffinose rather than glucose) [86].
(EPS)

S1 File C++ and XPP codes. (ZIP)

S1 Table Parameter values for the multisite phosphorylation model of the Start transition. (DOCX)

S2 Table Initial conditions for simulations of the multisite phosphorylation model of the Start transition in Figs 3 and 5. (DOCX)

S3 Table List of mutant strains used to test our deterministic model of the full cell cycle system. (DOCX)

S4 Table Parameter changes and initial conditions used to simulate mutant alleles. (DOCX)

S5 Table Rules for inviable mutant phenotypes. (DOCX)

S6 Table Inconsistencies between simulations and observations. (DOCX)

S1 Text Equations for the multisite phosphorylation model of the Start transition. (DOC)

S2 Text Derivation of the mRNA-inherited noise term. (DOC)

S3 Text Equations for the stochastic SCM of the Start transition. (DOC)

S4 Text Equations for the stochastic SCM of the Start transition with explicit mRNA species. (DOC)

S5 Text Mutant simulations and discussion of problems. (DOC)

S6 Text Model conversion. (DOC)

S7 Text The mRNA-inherited noise term of the full budding yeast cell cycle model. (DOC)

S8 Text The effects of the parameters <*m*_min_> on the model. (DOC)

S9 Text Simulation methods. (DOC)

S10 Text The effects of the integration step size (Δ*t*) on the model. (DOC)

## Abbreviations

CLE: chemical Langevin equation
MultiP: multisite phosphorylation
ODE: ordinary differential equation
PRN: protein regulatory network
SCM: standard component model
SSA: stochastic simulation algorithm
