# Supplementary figures and images for: A Model of Yeast Cell-Cycle Regulation Based on a Standard Component Modeling Strategy for Protein Regulatory Networks
Source: PLoS One. 2016 May 17;11(5):e0153738. doi: 10.1371/journal.pone.0153738 (PMC4871373; doi:10.1371/journal.pone.0153738)

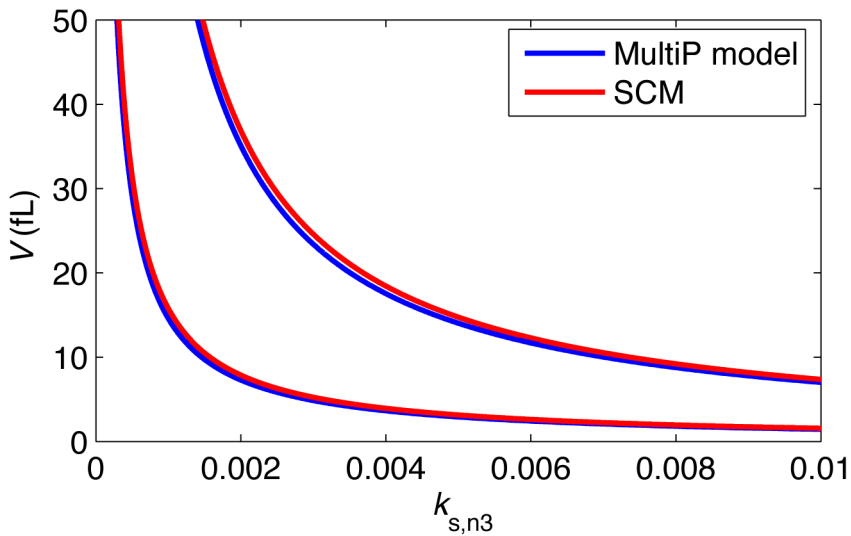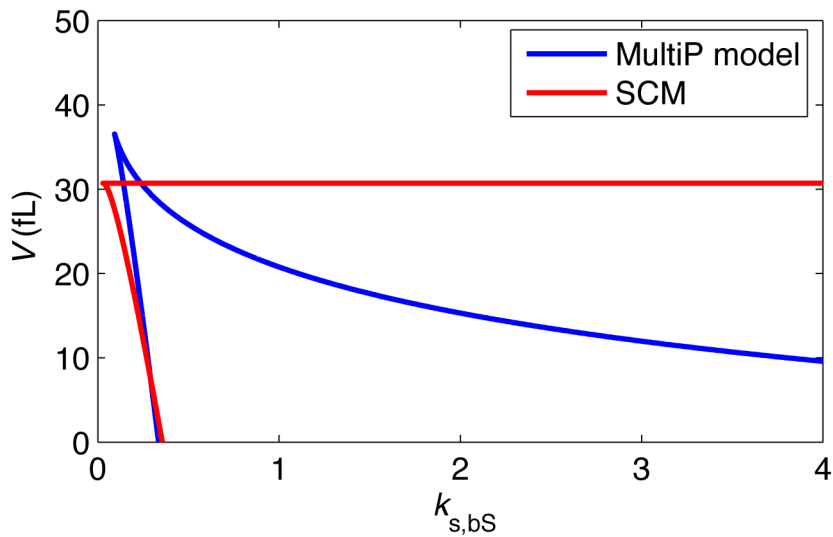

Supplement: S2 Fig — Upper panel: The two-parameter bifurcation diagrams show similar regions of bistability between the two models when the synthesis rate of Cln3 (ks,n3) and (fixed) cell volume are varied. Lower panel: However, at large synthesis rates of ClbS (ks,bS), the values of (fixed) cell volume that exhibit bistability are different between the two models. The value of ks,bS used in both models is 0.3 fL−1 min−1 (taken from the value used by Barik et al. [27]). At this value, both models show a similar bistability region when (fixed) cell volume is varied. (PDF) [file pone.0153738.s002.pdf]
