# Supplementary material for: A Model of Yeast Cell-Cycle Regulation Based on a Standard Component Modeling Strategy for Protein Regulatory Networks
Source: PLoS One. 2016 May 17;11(5):e0153738. doi: 10.1371/journal.pone.0153738 (PMC4871373; doi:10.1371/journal.pone.0153738)
Supplement: S1 Table — (DOCX) [file pone.0153738.s011.docx]

S1 Table. Parameter values for the multisite phosphorylation model of the Start transition.

| Parameter | Description | Value |
| --- | --- | --- |
| *k*_a,g_, *k*_d,bf_, etc. | Common parameters with SCM | Same as SCM |
| *k*_a,c_ | Rate constant for association of SBF and Whi5 | 0.1 fL molec^−1^ min^−1^ |
| *k*_dp,i5_ | Rate constant for dephos’n of Whi5 by Hi5 | 0.0096 fL molec^−1^ min^−1^ |
| *k*_p,i5_ | Rate constant for phos’n of Whi5 by Cln3 | 0.26 fL molec^−1^ min^−1^ |
| *k*^'^_p,i5_ | Rate constant for phos’n of Whi5 by ClbS | 0.02 fL molec^−1^ min^−1^ |
