# Supplementary material for: A Model of Yeast Cell-Cycle Regulation Based on a Standard Component Modeling Strategy for Protein Regulatory Networks
Source: PLoS One. 2016 May 17;11(5):e0153738. doi: 10.1371/journal.pone.0153738 (PMC4871373; doi:10.1371/journal.pone.0153738)
Supplement: S2 Table — (DOCX) [file pone.0153738.s012.docx]

S2 Table. Initial conditions for simulations of the multisite phosphorylation model of the Start transition in Figs 3 and 5.

| Variable | Number | Concentration |
| --- | --- | --- |
| *ClbS* | 0 | 0 nM |
| *Cln3* | 0 | 0 nM |
| *Cmp* | 1530 | 255 nM |
| *CmpP_i_* (*i* = 1, 2) | 0 | 0 nM |
| *Hi5* | 1275 | 213 nM |
| *m*_bS_ | 0 | 0 nM |
| *m*_hi5_ | 10 | 1.67 nM |
| *m*_i5_ | 8 | 1.33 nM |
| *m*_n3_ | 8 | 1.33 nM |
| *SBF* | 0 | 0 nM |
| *Whi5* | 5363 | 894 nM |
| *Whi5_i_* (*i* = 1–6) | 0 | 0 nM |
| *G*_a_ | 0 |  |
| *V* | 10 fL |  |
