# Supplementary material for: A Model of Yeast Cell-Cycle Regulation Based on a Standard Component Modeling Strategy for Protein Regulatory Networks
Source: PLoS One. 2016 May 17;11(5):e0153738. doi: 10.1371/journal.pone.0153738 (PMC4871373; doi:10.1371/journal.pone.0153738)
Supplement: S3 Table — (DOCX) [file pone.0153738.s013.docx]

**S3 Table. List of mutant strains used to test our deterministic model of the full cell cycle system.**

Numbers in parentheses indicate size at division compared to a wild-type cell.

| **Wild-type** | Simulation | Experiment |
| --- | --- | --- |
| In glucose | Viable (1.0) | Viable (1.0) |
| In galactose | Viable (0.86) | Viable (0.73) |

| **Cln3 and Bck2 mutants** | Simulation | Experiment |
| --- | --- | --- |
| $cln3\Delta$ | Viable (2.8) | Viable (1.8–2.7) |
| $bck2\Delta$ | Viable (1.5) | Viable (1.3–1.5) |
| $cln3\Delta\text{ }bck2\Delta$ | G_1_ arrest | G_1_ arrest |
| $cln3\Delta\text{ }bck2\Delta\text{ multicopy }CLN2$ | G_1_ arrest | G_1_ arrest |
| $cln3\Delta\text{ }bck2\Delta\text{ }sic1\Delta$ | G_1_ arrest | G_1_ arrest |
| $cln3\Delta\text{ }bck2\Delta\text{ }whi5\Delta\text{ (*)}$ | Viable (0.72) | Viable (1.4) |
| $GAL\text{-}CLN3$ | Viable (0.82) | Viable (0.44) |
| $\text{Multicopy }BCK2$ | Viable (0.72) | Viable (0.8) |

| **Cln1,2 mutants** | Simulation | Experiment |
| --- | --- | --- |
| $cln1\Delta\text{ }cln2\Delta$ | Viable (3.42) | Viable (3.2) |
| $cln1\Delta\text{ }cln2\Delta\text{ }bck2\Delta$ | Viable (4.02) | Viable (1.7) |
| $cln1\Delta\text{ }cln2\Delta\text{ }sic1\Delta$ | Viable | Viable |
| $cln1\Delta\text{ }cln2\Delta\text{ }cki\Delta$ | Viable | Viable |
| $cln1\Delta\text{ }cln2\Delta\text{ }GAL\text{-}SIC1$ | G_1_ arrest | G_1_ arrest |
| $cln1\Delta\text{ }cln2\Delta\text{ }GAL\text{-}CLN2$ | Viable (0.83) | Viable (0.5) |
| $\begin{matrix} cln1\Delta\text{ }cln2\Delta\text{ }GAL\text{-}SIC1\text{ } \\ GAL\text{-}CLN2 \end{matrix}$ | Viable | Viable |
| $cln1\Delta\text{ }cln2\Delta\text{ }cdh1\Delta$ | Viable | Viable |
| $\begin{matrix} cln1\Delta\text{ }cln2\Delta\text{ }cdh1\Delta\\ GAL\text{-}SIC1 \end{matrix}$ | G_1_ arrest | G_1_ arrest |
| $\begin{matrix} cln1\Delta\text{ }cln2\Delta\text{ }cdh1\Delta\\ GAL\text{-}CLN2 \end{matrix}$ | Viable | Viable |
| $\begin{matrix} cln1\Delta\text{ }cln2\Delta\text{ }cdh1\Delta\\ GAL\text{-}SIC1\text{ }GAL\text{-}CLN2 \end{matrix}$ | Viable | Viable |

| **Cln1,2 Cln3 mutants** | Simulation | Experiment |
| --- | --- | --- |
| $cln1\Delta\text{ }cln2\Delta\text{ }cln3\Delta$ | G_1_ arrest | G_1_ arrest |
| $\begin{matrix} cln1\Delta\text{ }cln2\Delta\text{ }cln3\Delta\\ GAL\text{-}CLN2 \end{matrix}$ | Viable | Viable |
| $\begin{matrix} cln1\Delta\text{ }cln2\Delta\text{ }cln3\Delta\\ GAL\text{-}CLN3 \end{matrix}$ | Viable | Viable |
| $cln1\Delta\text{ }cln2\Delta\text{ }cln3\Delta\text{ }sic1\Delta$ | Viable | Viable |
| $\begin{matrix} cln1\Delta\text{ }cln2\Delta\text{ }cln3\Delta\\ \text{multicopy }BCK2 \end{matrix}$ | Viable | Viable |
| $cln3\Delta\text{ }bck2\Delta\text{ }GAL-CLN2$ | Viable | Viable |
| $\begin{matrix} cln1\Delta\text{ }cln2\Delta\text{ }cln3\Delta\\ \text{multicopy }CLB5 \end{matrix}$ | Viable | Viable |
| $\begin{matrix} cln1\Delta\text{ }cln2\Delta\text{ }cln3\Delta\\ GAL\text{-}CLB5 \end{matrix}$ | Viable | Viable |
| $\begin{matrix} cln1\Delta\text{ }cln2\Delta\text{ }cln3\Delta\\ GAL\text{-}CLB2 \end{matrix}$ | G_1_ arrest | G_1_ arrest |
| $cln1\Delta\text{ }cln2\Delta\text{ }cln3\Delta\text{ }cdh1\Delta$ | Telophase | Inviable |
| $cln1\Delta\text{ }cln2\Delta\text{ }cln3\Delta\text{ }apc\text{-}ts$ | Metaphase arrest | Metaphase arrest |

| **CKI and Cdh1 mutants** | Simulation | Experiment |
| --- | --- | --- |
| $cdh1\Delta$ | Viable | Viable |
| $CDH1\text{ constitutively active}$ | S/G_2_ arrest | G_2_ arrest |
| $sic1\Delta$ | Viable | Viable |
| $GAL\text{-}SIC1$ | Viable | Viable |
| $GAL\text{-}SIC1\text{-}db\Delta$ | G_1_ arrest | G_1_ arrest |
| $cki\Delta$ | Viable | Viable |
| $swi5\Delta$ | Viable | Viable |
| $sic1\Delta\text{ }cdh1\Delta$ | Inviable | Inviable |
| $cki\Delta\text{ }cdh1\Delta$ | Inviable | Inviable |
| $swi5\Delta\text{ }cdh1\Delta$ | Inviable | Inviable |
| $\begin{matrix} swi5\Delta\text{ }cdh1\Delta\\ GAL\text{-}SIC1 \end{matrix}$ | Viable | Viable |

| **Clb5,6 mutants** | Simulation | Experiment |
| --- | --- | --- |
| $clb5\Delta\text{ }clb6\Delta$ | Viable | Viable |
| $clb5\Delta\text{ }clb6\Delta\text{ }cln1\Delta\text{ }cln2\Delta$ | G_1_ arrest | G_1_ arrest |
| $CLB5\text{-}db\Delta$ | Viable | Viable |
| $CLB5\text{-}db\Delta\text{ }sic1\Delta$ | Origin relicensing problems | Origin relicensing problems |
| $GAL\text{-}CLB5$ | Viable | Viable |
| $GAL\text{-}CLB5\text{ }sic1\Delta$ | Origin relicensing problems | Origin relicensing problems |
| $GAL\text{-}CLB5\text{ }cdh1\Delta$(**) | Viable | Expected to be viable |
| $GAL\text{-}CLB5\text{-}db\Delta\text{ (*)}$ | Telophase arrest | Origin relicensing problems |

| **Clb1,2 mutants** | Simulation | Experiment |
| --- | --- | --- |
| $clb1\Delta\text{ }clb2\Delta$ | S/G_2_ arrest | G_2_ arrest |
| $\begin{matrix} clb1\Delta\text{ }clb2\Delta\\ clb5\Delta\text{ }clb6\Delta\end{matrix}$ | G_1_ arrest | G_1_ arrest |
| $GAL\text{-}CLB2$ | Viable | Viable |
| $\text{Multicopy }GAL\text{-}CLB2$ | Telophase arrest | Telophase arrest |
| $GAL\text{-}CLB2\text{ }sic1\Delta$ | Telophase arrest | Telophase arrest |
| $GAL\text{-}CLB2\text{ }cdh1\Delta$ | Telophase arrest | Telophase arrest |
| $GAL\text{-}CLB2\text{ }swi5\Delta$ | Telophase arrest | Telophase arrest |
| $CLB2\text{-}db\Delta$ | Telophase arrest | Telophase arrest |
| $\begin{matrix} CLB2\text{-}db\Delta\\ \text{in raffinose} \end{matrix}$ | Telophase arrest | Telophase arrest |
| $\begin{matrix} CLB2\text{-}db\Delta\\ GAL\text{-}SIC1 \end{matrix}$ | Viable | Viable |
| $\begin{matrix} CLB2\text{-}db\Delta\\ \text{multicopy }SIC1 \end{matrix}$ | Viable | Viable |
| $CLB2\text{-}db\Delta\text{ }clb5\Delta\text{ }clb6\Delta$ | Telophase arrest | Telophase arrest |
| $\begin{matrix} CLB2\text{-}db\Delta\text{ }clb5\Delta\text{ }clb6\Delta\\ \text{in raffinose} \end{matrix}$ | Viable | Viable |
| $\begin{matrix} CLB2\text{-}db\Delta\text{ }clb5\Delta\text{ }clb6\Delta\\ GAL\text{-}SIC1 \end{matrix}$ | Viable | Viable |
| $GAL\text{-}CLB2\text{-}db\Delta$ | Telophase arrest | Telophase arrest |
| $CLB1\text{ }clb2\Delta$ | Viable | Viable |
| $CLB1\text{ }clb2\Delta\text{ }cdh1\Delta\text{ (*)}$ | Viable | Inviable |
| $CLB1\text{ }clb2\Delta\text{ }pds1\Delta\text{ (*)}$ | Viable | Inviable |

| **Cdc20 mutants** | Simulation | Experiment |
| --- | --- | --- |
| $cdc20\text{-}ts$ | Metaphase arrest | Metaphase arrest |
| $clb5\Delta\text{ }clb6\Delta\text{ }cdc20\Delta\text{ }$ | Metaphase arrest | Metaphase arrest |
| $cdc20\Delta\text{ }pds1\Delta$ | Telophase arrest | Telophase arrest |
| $clb5\Delta\text{ }clb6\Delta\text{ }cdc20\Delta\text{ }pds1\Delta$ | Viable | Viable |
| $CLB5\text{-}db\Delta\text{ }cdc20\Delta\text{ }pds1\Delta$ | Telophase arrest | Telophase arrest |
| $CLB5\text{-}db\Delta\text{ }pds1\Delta$ | Viable | Viable |
| $GAL\text{-}CDC20$ | Mitotic catastrophe | Mitotic catastrophe |
| $GALL\text{-}CDC20\text{ }sic1\Delta\text{ }cdh1\Delta$ | Viable | Viable |
| $GALL\text{-}CDC20\text{ }cki\Delta\text{ }cdh1\Delta$ | Viable | Viable |

| **APC mutants** | Simulation | Experiment |
| --- | --- | --- |
| $APC\text{-}A$ | Viable | Viable |
| $APC\text{-}A\text{ }sic1\Delta$ | Viable | Viable |
| $APC\text{-}A\text{ }cki\Delta$ | Viable | Viable |
| $APC\text{-}A\text{ }cdh1\Delta$ | Telophase arrest | Telophase arrest |
| $APC\text{-}A\text{ }cdh1\Delta\text{ in galactose}$ | Telophase arrest | Telophase arrest |
| $APC\text{-}A\text{ }cdh1\Delta\text{ }GAL\text{-}SIC1$ | Viable | Viable |
| $\begin{matrix} APC\text{-}A\text{ }cdh1\Delta\text{ } \\ \text{multicopy }SIC1 \end{matrix}$ | Viable | Viable |
| $\begin{matrix} APC\text{-}A\text{ }cdh1\Delta\text{ } \\ \text{multicopy }CDC20 \end{matrix}$ | Viable | Viable |
| $APC\text{-}A\text{ }GAL\text{-}CLB2\text{ }$ | Telophase arrest | Telophase arrest |

| **FEAR pathway mutants** | Simulation | Experiment |
| --- | --- | --- |
| $pds1\Delta$ | Viable | Viable |
| $PDS1\text{-}db\Delta$ | Inviable | Inviable |
| $GAL\text{-}PDS1\text{-}db\Delta$ | Inviable | Inviable |
| $esp1\text{-}ts$ | Inviable | Inviable |
| $GAL\text{-}PDS1\text{-}db\Delta\text{ }esp1\text{-}ts$ | Inviable | Inviable |
| $GAL\text{-}ESP1\text{ }cdc20\text{-}ts$ | Inviable | Inviable |
| $cdc55\Delta$ | Viable | Viable |
| $GAL\text{-}CDC55$ | Viable | Viable |

| **MEN pathway mutants** | Simulation | Experiment |
| --- | --- | --- |
| $tem1\Delta$ | Telophase arrest | Telophase arrest |
| $net1\text{-}ts$ | Viable | Viable |
| $tem1\Delta\text{ }net1\text{-}ts$ | Viable | Viable |
| $GAL\text{-}TEM1$ | Viable | Viable |
| $tem1\text{-}ts\text{ }GAL\text{-}CDC15$ | Viable | Viable |
| $\begin{matrix} tem1\text{-}ts\text{ } \\ \text{multicopy }CDC14 \end{matrix}$ | Viable | Viable |
| $\text{Multicopy }CDC15$ | Viable | Viable |
| $\begin{matrix} tem1\text{-}ts \\ \text{multicopy }CDC15 \end{matrix}$ | Viable | Viable |
| $net1\text{-}ts\text{ }cdc20\text{-}ts$ | Inviable | Inviable |
| $cdc15\Delta$ | Telophase arrest | Telophase arrest |
| $cdc15\Delta\text{ }net1\text{-}ts$ | Viable | Viable |
| $cdc15\Delta\text{ }net1\text{-}ts\text{ }cdh1\Delta\text{ (*)}$ | Inviable | Viable |
| $cdc15\text{-}ts\text{ multicopy }TEM1$ | Telophase arrest | Telophase arrest |
| $cdc15\text{-}ts\text{ multicopy }CDC14$ | Viable | Viable |
| $TAB6\text{-}1$ | Viable | Viable |
| $TAB6\text{-}1\text{ }cdc15\Delta$ | Viable | Viable |
| $TAB6\text{-}1\text{ }clb5\Delta\text{ }clb6\Delta$ | G_1_ arrest | G_1_ arrest |
| $TAB6\text{-}1\text{ }CLB1\text{ }clb2\Delta$ | Viable | Viable |
| $cdc14\text{-}ts$ | Telophase arrest | Telophase arrest |
| $cdc14\text{-}ts\text{ }sic1\Delta$ | Telophase arrest | Telophase arrest |
| $cdc14\text{-}ts\text{ }cdh1\Delta$ | Telophase arrest | Telophase arrest |
| $cdc14\text{-}ts\text{ }GAL\text{-}SIC1$ | Telophase arrest | Telophase arrest |
| $cdc14\text{-}ts\text{ }GAL\text{-}CLN2$ | Telophase arrest | Telophase arrest |
| $GAL\text{-}NET1$ | Telophase arrest | Telophase arrest |
| $GAL\text{-}CDC14$ | G_1_ arrest | G_1_ arrest |
| $GAL\text{-}NET1\text{ }GAL\text{-}CDC14$ | Viable | Viable |

| **Checkpoint mutants** | Simulation | Experiment |
| --- | --- | --- |
| $mad2\Delta$ | Viable | Viable |
| $bub2\Delta$ | Viable | Viable |
| $mad2\Delta\text{ }bub2\Delta$ | Viable | Viable |
| $bub2\Delta\text{ }cdc20\text{-}ts$ | Metaphase arrest | Metaphase arrest |
| $mad2\Delta\text{ }cdc20\text{-}ts$ | Metaphase arrest | Metaphase arrest |
| $\begin{matrix} \text{Wild-type cells} \\ \text{in nocodazole} \end{matrix}$ | Metaphase arrest | Metaphase arrest |
| $mad2\Delta\text{ in nocodazole}$ | Not arrested | Not arrested |
| $bub2\Delta\text{ in nocodazole (*)}$ | Arrested | Not arrested |
| $\begin{matrix} mad2\Delta\text{ }bub2\Delta\text{ } \\ \text{in nocodazole} \end{matrix}$ | Not arrested | Not arrested |
| $pds1\Delta\text{ in nocodazole}$ | Arrested | Arrested |
| $\begin{matrix} mad2\Delta\text{ }pds1\Delta\text{ } \\ \text{in nocodazole} \end{matrix}$ | Not arrested | Not arrested |
| $\begin{matrix} bub2\Delta\text{ }pds1\Delta\text{ } \\ \text{in nocodazole} \end{matrix}$ (*) | Arrested | Not arrested |
| $net1\text{-}ts\text{ in nocodazole (*)}$ | Arrested | Not arrested |
| $\begin{matrix} mad2\Delta\text{ }GAL\text{-}TEM1\text{ } \\ \text{in nocodazole} \end{matrix}$ | Not arrested | Not arrested |

* These simulated mutant stains do not agree with experimental observations. They are collected for closer examination in S6 Table and discussed in detail in S5 Text.

** *GAL-CLB5* *cdh1*Δ cells were reported to go through several doublings in galactose, but were ultimately inviable without known causes.
