# Supplementary material for: A Model of Yeast Cell-Cycle Regulation Based on a Standard Component Modeling Strategy for Protein Regulatory Networks
Source: PLoS One. 2016 May 17;11(5):e0153738. doi: 10.1371/journal.pone.0153738 (PMC4871373; doi:10.1371/journal.pone.0153738)
Supplement: S4 Table — (DOCX) [file pone.0153738.s014.docx]

**S4 Table. Parameter changes and initial conditions used to simulate mutant alleles.**

For mutant strains that carry combinations of these alleles, we used combinations of these parameter changes.

| Mutant allele | Parameter change(s) |
| --- | --- |
| $GAL\text{-}SIC1$* | $\text{Galactose*, }k_{\text{s,cki}}=0.36\text{ (}=1.5\times\text{WT }k_{\text{s,cki,swi5}})$ |
| $GAL\text{-}CLN2$ | $\text{Galactose, }k_{\text{s,cln2}}=0.27\text{ (}=\text{WT }k_{\text{s,cln2,bf}})$ |
| $GAL\text{-}CLN3$ | $\text{Galactose, }D_{\text{cln3}}=20\text{ (20}\times\text{WT})$ |
| $GAL\text{-}CLB5$ | $\text{Galactose, }k_{\text{s,clb5}}=0.058\text{ (}=2\times\text{WT }k_{\text{s,clb5,bf}})$ |
| $GAL\text{-}CLB2$ | $\text{Galactose, }k_{\text{s,clb2}}=0.308\text{ (}=7\times\text{WT }k_{\text{s,clb2,m1}})$ |
| $GAL\text{-}CDC20$ | $\text{Galactose, }k_{\text{s,cdc20}}=7\text{ (}=11.6\times\text{WT }k_{\text{s,cdc20,m1}})$ |
| $GALL\text{-}CDC20$ | $\text{Galactose, }k_{\text{s,cdc20}}=5\text{ (}=8.3\times\text{WT }k_{\text{s,cdc20,m1}})$ |
| $GAL\text{-}ESP1$ | $\text{Galactose, [Esp1}_{\text{T}}\text{]}_{\text{n}}=1\text{ (2}\times\text{WT})$ |
| $GAL\text{-}PDS1$ | $\text{Galactose, }k_{\text{s,pds1}}=0.099\text{ (3.3}\times\text{WT})$ |
| $GAL\text{-}CDC55$ | $\text{Galactose, [PPX}_{\text{T}}\text{]}_{\text{n}}=2\text{ (2}\times\text{WT})$ |
| $GAL\text{-}CDC15$ | $\text{Galactose, [Cdc15}_{\text{T}}\text{]}_{\text{n}}=12\text{ (12}\times\text{WT})$ |
| $GAL\text{-}NET1$ | $\text{Galactose, [Net1}_{\text{T}}\text{]}_{\text{n}}=24.85\text{ (7}\times\text{WT})$ |
| $GAL\text{-}CDC14$ | $\text{Galactose, [Cdc14}_{\text{T}}\text{]}_{\text{n}}=6\text{ (3}\times\text{WT})$ |
| $\text{Multicopy }BCK2$ | $k_{\text{s,bck2}}=\text{0.11475 (17 copies)}$ |
| $\text{Multicopy }CLN2$ | $k_{\text{s,cln2,bf}}=\text{0.54 (2 copies)}$ |
| $\text{Multicopy }CLB5$ | $k_{\text{s,clb5}}=\text{0.003, }k_{\text{s,clb5,bf}}=\text{0.145 (5 copies)}$ |
| $\text{Multicopy }SIC1$ | $k_{\text{s,cki}}=\text{0.432, }k_{\text{s,cki,swi5}}=\text{4.32 (18 copies)}$ |
| $\text{Multicopy }CDC20$ | $k_{\text{s,cdc20}}=0.012\text{, }k_{\text{s,cdc20,m1}}=1.2\text{ (2 copies)}$ |
| $\text{Multicopy }CDC15$ | $\text{[Cdc15}_{\text{T}}\text{]}_{\text{n}}=3\text{ (3 copies)}$ |
| $\text{Multicopy }TEM1$ | $\text{[Tem1}_{\text{T}}\text{]}_{\text{n}}=4\text{ (2 copies)}$ |
| $\text{Multicopy }CDC14$ | $\text{[Cdc14}_{\text{T}}\text{]}_{\text{n}}=4\text{ (2 copies)}$ |
| **$cln3\Delta$** | **$D_{\text{cln3}}=\text{[Cln3]}_{\text{n}}=0$** |
| $bck2\Delta$ | $k_{\text{s,bck2}}=\text{[Bck2]}_{\text{n}}=0$ |
| $sic1\Delta$** | $k_{\text{s,cki}}=0.003,\text{ }k_{\text{s,cki,swi5}}=0.03,\text{ [CKI}_{\text{T}}\text{]}_{\text{n}}=\text{[CKI}_{\text{P}}\text{]}_{\text{n}}=0$ |
| $cki\Delta$ | $k_{\text{s,cki}}=\text{ }k_{\text{s,cki,swi5}}=\text{ [CKI}_{\text{T}}\text{]}_{\text{n}}=\text{ [CKI}_{\text{P}}\text{]}_{\text{n}}=0$ |
| $whi5\Delta$ | $\text{[Whi5}_{\text{T}}\text{]}_{\text{n}}=\text{ [Whi5}_{\text{A}}\text{]}_{\text{n}}=0$ |
| $cln2\Delta$ | $k_{\text{s,cln2}}=\text{ }k_{\text{s,cln2,bf}}=\text{ [Cln2]}_{\text{n}}=0$ |
| $cdh1\Delta$ | $\text{[Cdh1}_{\text{T}}\text{]}_{\text{n}}=\text{ [Cdh1}_{\text{A}}\text{]}_{\text{n}}=0$ |
| $apc\text{-}ts$ | $k_{\text{s,cdc20}}=\text{ }k_{\text{s,cdc20,m1}}=\text{ [Cdc20}_{\text{T}}\text{]}_{\text{n}}=\text{[Cdh1}_{\text{T}}\text{]}_{\text{n}}=\text{[Cdh1}_{\text{A}}\text{]}_{\text{n}}=0\text{ }$ |
| *CDH1* constitutively active | $\text{Galactose, [Cdh1}_{\text{T}}\text{]}_{\text{n}}=3,\text{ }\omega_{\text{i,cdh1,e}}=0$ |
| $GAL\text{-}SIC1\text{-}db\Delta$ | $GAL\text{-}SIC1,\text{ }k_{\text{d,ckip}}=0$ |
| $swi5\Delta$ | $k_{\text{s,swi5}}=\text{ }k_{\text{s,swi5,m1}}=\text{ [Swi5}_{\text{T}}\text{]}_{\text{n}}=0\text{ }$ |
| $clb5\Delta$ | $k_{\text{s,clb5}}=\text{ }k_{\text{s,clb5,bf}}=\text{ [Clb5}_{\text{T}}\text{]}_{\text{n}}=0\text{ }$ |
| $CLB5\text{-}db\Delta$ | $k_{\text{d,clb5,20}}=\text{ }k_{\text{d,clb5,20,i}}=0$ |
| $GAL\text{-}CLB5\text{-}db\Delta$ | $GAL\text{-}CLB5\text{, }k_{\text{d,clb5,20}}=\text{ }k_{\text{d,clb5,20,i}}=0$ |
| $clb1\Delta clb2\Delta$ | $k_{\text{s,clb2}}=\text{ }k_{\text{s,clb2,m1}}=\text{ [Clb2}_{\text{T}}\text{]}_{\text{n}}=0\text{ }$ |
| $\text{multicopy }GAL\text{-}CLB2$ | 2 copies of *GAL-CLB2* |
| $CLB2\text{-}db\Delta$ | $k_{\text{d,clb2,20}}=\text{ }k_{\text{d,clb2,20,i}}=0,\text{ }k_{\text{d,clb2,h1}}=8.5\%\text{ of WT}$ |
| $CLB1\text{ }clb2\Delta$ | $k_{\text{s,clb2}}=33\%\text{ of WT},\text{ }k_{\text{s,clb2,m1}}=33\%\text{ of WT}$ |
| $cdc20\Delta$ | $k_{\text{s,cdc20}}=\text{ }k_{\text{s,cdc20,m1}}=\text{ [Cdc20}_{\text{T}}\text{]}_{\text{n}}=0\text{ }$ |
| $cdc20\text{-}ts$ | $cdc20\Delta$ |
| $APC\text{-}A$ | $\omega_{\text{a,apc,b2}}=0,\text{ [APC}_{\text{P}}]_{\text{n}}=0$ |
| $pds1\Delta$ | $k_{\text{s,pds1}}=\text{ [Pds1}_{\text{T}}\text{]}_{\text{n}}=0\text{ }$ |
| $PDS1\text{-}db\Delta$ | $k_{\text{d,pds1,20}}=\text{ }k_{\text{d,pds1,20,i}}=0\text{ }$ |
| $GAL\text{-}PDS1\text{-}db\Delta$ | $GAL\text{-}PDS1,\text{ }k_{\text{d,pds1,20}}=\text{ }k_{\text{d,pds1,20,i}}=0\text{ }$ |
| $esp1\text{-}ts$ | $\text{[Esp1}_{\text{T}}\text{]}_{\text{n}}=0\text{ }$ |
| $cdc55\Delta$ | $\text{[PPX}_{\text{T}}\text{]}_{\text{n}}=\text{ [PPX}_{\text{A}}\text{]}_{\text{n}}=0\text{ }$ |
| $tem1\Delta$ | $\text{[Tem1}_{\text{T}}\text{]}_{\text{n}}=\text{ [Tem1}_{\text{A}}\text{]}_{\text{n}}=0\text{ }$ |
| $tem1\text{-}ts$ | $tem1\Delta$ |
| $net1\text{-}ts$*** | $\rho_{\text{14,net1}}=0.45$ |
| $cdc15\Delta$ | $\text{[Cdc15}_{\text{T}}\text{]}_{\text{n}}=\text{ [Cdc15}_{\text{A}}\text{]}_{\text{n}}=0\text{ }$ |
| $cdc15\text{-}ts$ | $cdc15\Delta$ |
| $TAB6\text{-}1$*** | $\rho_{\text{14,net1}}=0.48$ |
| $cdc14\text{-}ts$ | $\text{[Cdc14}_{\text{T}}\text{]}_{\text{n}}=0$ |
| $mad2\Delta$ | $\omega_{\text{a,mad2}}=\text{ [Mad2}_{\text{A}}\text{]}_{\text{n}}=0$ |
| $bub2\Delta$ | $\omega_{\text{i,tem1}}=\omega_{\text{i,tem1,px}}=0$ |
| $\text{Cell in nocodazole}$ | $k_{\text{s,spn}}=0$ |

* In galactose medium, *mdt* =150 min, and mother:daughter size ratio at birth = 61:39.

** Since the ratio between Sic1 and Cdc6 is 1:2, the synthesis rate of CKI in $sic1\Delta$ cells should drop to 2/3. However, we reduce the synthesis rate of CKI in this mutant to ~1/8 to account for the fact that Cdc6 does not bind to Clb5 and Clb2 as strongly as Sic1 does.

*** We set the parameter *ρ*_14,net1_ < 1 to reproduce the weaker interaction between Net1 and Cdc14 in strains carrying these alleles.
