# Supplementary material for: A Model of Yeast Cell-Cycle Regulation Based on a Standard Component Modeling Strategy for Protein Regulatory Networks
Source: PLoS One. 2016 May 17;11(5):e0153738. doi: 10.1371/journal.pone.0153738 (PMC4871373; doi:10.1371/journal.pone.0153738)
Supplement: S5 Table — (DOCX) [file pone.0153738.s015.docx]

**S5 Table. Rules for inviable mutant phenotypes.**

| Phase | Condition |
| --- | --- |
| G_1_ arrest | [ORI]_n_ does not reach 1 by the time *V*_n_ reaches 8.64* for cells in glucose medium and 8.19* for cells in galactose or raffinose medium |
| S/G_2_ arrest | Clb2 is not activated (i.e., [Clb2]_n_ < 0.2) by the time *V*_n_ reaches 8.64* for cells in glucose medium and 8.19* for cells in galactose or raffinose medium |
| Metaphase arrest | Esp1 is not activated (i.e., [Esp1]_n_ < 0.1) and cells do not divide |
| Telophase arrest | Esp1 is activated (i.e., [Esp1]_n_ ≥ 0.1) but cells do not divide |
| Origin relicensing problems | [Clb5]_n_ +[Clb2]_n_ never goes below *K*_EZ2_ as cells exit the previous mitosis |
| Mitotic catastrophe | Esp1 is activated before spindle assembly is complete (i.e., [Esp1]_n_ ≥ 0.1 when SPN(*t*) = 1). |

*A newborn daughter cell has $V_{\text{n}}\approx1$(28 fL). *V*_n_ = 8.64 is equal to 242 fL, which is 4.5× size at which [ORI]_n_ reaches 1 in WT cells in glucose. *V*_n_ = 8.19 is equal to 229 fL, which is 4.5× size at which [ORI]_n_ reaches 1 in WT cells in galactose or raffinose.
