# Supplementary material for: A Model of Yeast Cell-Cycle Regulation Based on a Standard Component Modeling Strategy for Protein Regulatory Networks
Source: PLoS One. 2016 May 17;11(5):e0153738. doi: 10.1371/journal.pone.0153738 (PMC4871373; doi:10.1371/journal.pone.0153738)
Supplement: S6 Table — (DOCX) [file pone.0153738.s016.docx]

**S6 Table. Inconsistencies between simulations and observations.**

| Genotype | Simulated phenotype | Observed phenotype |
| --- | --- | --- |
| $cln3\Delta\text{ }bck2\Delta\text{ }whi5\Delta$ | Mutant cells are smaller than wild-type cells (70%) | Mutant cells are bigger than wild-type cells (140%) |
| $GAL\text{-}CLB5\text{-}db\Delta$ | Cells arrest in telophase during the first cycle | Cells exit mitosis but die in the next cycle due to DNA replication problems |
| $CLB1\text{ }clb2\Delta\text{ }cdh1\Delta$ | Cells are viable | Cells are inviable |
| $CLB1\text{ }clb2\Delta\text{ }pds1\Delta$ | Cells are viable | Cells are inviable |
| $cdc15\Delta\text{ }net1\text{-}ts\text{ }cdh1\Delta$ | Cells arrest in telophase | Cells are viable |
| $bub2\Delta\text{ in nocodazole}$ | Cells arrest in metaphase | Cells exit mitosis without chromosome segregation |
| $\begin{matrix} bub2\Delta\text{ }pds1\Delta\text{ } \\ \text{in nocodazole} \end{matrix}$ | Cells arrest in telophase | Cells exit mitosis without chromosome segregation |
| $net1\text{-}ts\text{ in nocodazole}$ | Cells arrest in metaphase | Cells exit mitosis without chromosome segregation |
