# Supplementary material for: A Model of Yeast Cell-Cycle Regulation Based on a Standard Component Modeling Strategy for Protein Regulatory Networks
Source: PLoS One. 2016 May 17;11(5):e0153738. doi: 10.1371/journal.pone.0153738 (PMC4871373; doi:10.1371/journal.pone.0153738)
Supplement: S1 Text — (DOC) [file pone.0153738.s017.doc]

**S1 Text. Equations for the multisite phosphorylation model of the Start transition**

The variables *Cln3*, *ClbS*, *Whi5*, etc. refer to numbers of protein molecules, and the variables *m*_n3_, *m*_bS_, *m*_i5_, etc. refer to numbers of mRNA molecules. The variable *G*_a_ is the probability that the *CLBS* gene is actively transcribed to *m*_bS_. Hi5 is the phosphatase that dephosphorylates Whi5. *V*(*t*) is cell volume in fL. The relationship between number of molecules of species *C*, concentration [C] in nM, and volume in fL is *C* = 0.6∙*V*∙[C]. The parameter values and initial conditions used for simulations of the MultiP model are given in S1 and S2 Tables, respectively.

**Equations:**

$$\frac{\text{d}V}{\text{d}t}=\mu\cdot V$$

$$\frac{\text{d}m_{\text{n3}}}{\text{d}t}=k_{\text{s,mn3}}-k_{\text{d,mn3}}\cdot m_{\text{n3}}$$

$$\frac{\text{d}Cln3}{\text{d}t}=k_{\text{s,n3}}\cdot V^{2}\cdot m_{\text{n3}}-k_{\text{d,n3}}\cdot Cln3$$

$$\frac{\text{d}m_{\text{bS}}}{\text{d}t}=k_{\text{s,mbS}}\cdot G_{\text{a}}-k_{\text{d,mbS}}\cdot m_{\text{bS}}$$

$$\frac{\text{d}ClbS}{\text{d}t}=k_{\text{s,bS}}\cdot V\cdot m_{\text{bS}}-k_{\text{d,bS}}\cdot ClbS$$

$$\frac{\text{d}G_{\text{a}}}{\text{d}t}=\frac{k_{\text{a,g}}}{V}\cdot SBF\cdot(1-G_{\text{a}})-k_{\text{d,g}}\cdot G_{\text{a}}$$

$$\frac{\text{d}m_{\text{hi5}}}{\text{d}t}=k_{\text{s,mhi5}}-k_{\text{d,mhi5}}\cdot m_{\text{hi5}}$$

$$\frac{\text{d}Hi5}{\text{d}t}=k_{\text{s,hi5}}\cdot V\cdot m_{\text{hi5}}-k_{\text{d,hi5}}\cdot Hi5$$

$$\frac{\text{d}m_{\text{i5}}}{\text{d}t}=k_{\text{s,mi5}}-k_{\text{d,mi5}}\cdot m_{\text{i5}}$$

**Rules:**

1) The Start transition occurs when [SBF] increases above 15 nM; i.e., $SBF/(0.6\cdot V)>15$.

2) The G_1_/S transition occurs when [ClbS] increases above 37.5 nM; i.e., $ClbS/(0.6\cdot V)>37.5$.
