# Supplementary material for: A Model of Yeast Cell-Cycle Regulation Based on a Standard Component Modeling Strategy for Protein Regulatory Networks
Source: PLoS One. 2016 May 17;11(5):e0153738. doi: 10.1371/journal.pone.0153738 (PMC4871373; doi:10.1371/journal.pone.0153738)
Supplement: S2 Text — (DOC) [file pone.0153738.s018.doc]

S2 Text. Derivation of the mRNA-inherited noise term

In this section, we show how to add a noise term to the deterministic ODE for protein production that approximately reproduces the CV^2^ of protein variation at steady state due to noise originating from the unmodeled mRNA production. For a stationary process, the stochastic equation can be described as an Ornstein-Uhlenbeck process

| $\frac{\text{d}u}{\text{d}t}=-\lambda(u-u^{*})+\sqrt{2\lambda V_{\text{u}}}\cdot\xi(t).$ | (A1) |
| --- | --- |

Here *u* drifts exponentially toward its mean value *u** at rate $\lambda$. The second term is the diffusion term, where *V*_u_ represents the variance of the fluctuations of *u*(*t*) at steady state and $\xi(t)=\frac{dW(t)}{dt}$ is the derivative of the Wiener process. When we numerically integrate Eq. A1 by Euler’s method with fixed step size $\Delta t$, then $\xi(t)\Delta t$ is a Gaussian random variable with mean zero and standard deviation $\sqrt{\Delta t}$.

In our case, we consider fluctuations of class-1 variables of the form

| $\frac{\Delta X_{i}}{\Delta t}=-B_{i}\left(X_{i}-\left\langle X_{i}\right\rangle\right)+\sqrt{2B_{i}V_{\text{X}}}\cdot\frac{\zeta(t)}{\sqrt{\Delta t}},$ | (A2) |
| --- | --- |

where *ζ*(*t*) is a Gaussian random variable with zero mean and unit standard deviation, *X_i_* represents the number of molecules of the component, $\left\langle\text{X}_{i}\right\rangle$ is the steady state of *X_i_* and *B_i_* is the degradation rate as described in Eq. 3 in the main text. We need to choose *V*_X_ to give the correct CV^2^ in protein numbers at steady state. It was shown by Pedraza & Paulsson that the CV^2^ of protein numbers caused by mRNA fluctuations (at steady state) follows the equation

| $\text{CV}_{\text{mRNA-inherited noise}}^{2}=\frac{1}{\left\langle m\right\rangle}\cdot\frac{\tau_{\text{m}}}{\tau_{\text{m}}+\tau_{\text{p}}},$ | (A3) |
| --- | --- |

where $\left\langle m\right\rangle$ is the average number of mRNA molecules at steady state, and $\tau_{\text{m}}$ and $\tau_{\text{p}}$ are half-lives of mRNAs and proteins, respectively.

Substituting $V_{\text{X}}=\sigma_{\text{X}}^{2}=\text{CV}^{2}\cdot\left\langle X_{i}\right\rangle^{2}$ into Eq. A2 yields

| $\begin{matrix} \frac{\Delta X_{i}}{\Delta t}=-B_{i}\left(X_{i}-\left\langle X_{i}\right\rangle\right)+\left\langle X_{i}\right\rangle\sqrt{2B_{i}\cdot\text{CV}^{2}}\cdot\frac{\zeta(t)}{\sqrt{\Delta t}} \\ =-B_{i}\left(X_{i}-\left\langle X_{i}\right\rangle\right)+\left\langle X_{i}\right\rangle\sqrt{2B_{i}\cdot\frac{1}{\left\langle m_{i}\right\rangle}\cdot\frac{\tau_{\text{m}}}{\tau_{\text{m}}+\tau_{\text{p}}}}\cdot\frac{\zeta(t)}{\sqrt{\Delta t}}. \end{matrix}$ | (A4) |
| --- | --- |

Then we set

| $\tau_{\text{m}}=\frac{\ln(2)}{k_{\text{dm}}}\text{ and }\tau_{\text{p}}=\frac{\ln(2)}{B_{i}},$ | (A5) |
| --- | --- |

where *k*_dm_ and *B_i_* represent the mRNA degradation rate and the protein degradation rate, respectively. This yields

| $\frac{\Delta X_{i}}{\Delta t}=-B_{i}\left(X_{i}-\left\langle X_{i}\right\rangle\right)+X_{i}\sqrt{2B_{i}\cdot\frac{1}{\left\langle m_{i}\right\rangle}\cdot\frac{B_{i}}{B_{i}+k_{\text{dm}}}}\cdot\frac{\zeta(t)}{\sqrt{\Delta t}},$ | (A6) |
| --- | --- |

where, in the second term, we approximate $\left\langle X_{i}\right\rangle\approx X_{i}$ for simplicity.

Equation (17) of the main text has “protein noise term” in addition to the “mRNA-inherited noise term” derived here. Since the deterministic part of the chemical Langevin equation is linear, the effects of the two noise terms are additive. Since the terms are zero-mean and uncorrelated, the variance of the sum is the sum of the variances. So the CV^2^ of the resulting Langevin equation is approximately the CV^2^ due to the protein noise plus the CV^2^ due the mRNA term. This is not exact, as we made the approximation <*X_i_*> ≈ *X_i_*, but will be close if the CV of *X_i_* is not too large.

**References**

1. Kampen NV. Stochastic processes in physics and chemistry: Amsterdam: Boston: Elsevier; 2007. 463 p.

2. Doering CR. Modeling Complex Systems: Stochastic Processes, Stochastic Differential Equations, and Fokker-Planck Equations. In **Lectures in Complex Systems: SFI Studies in the Sciences of Complexity,** Vol. III. Edited by L Nadel & D Stein: Addison-Wesley1991.

3. Pedraza JM, Paulsson J. Effects of molecular memory and bursting on fluctuations in gene expression. Science. 2008;319(5861):339-43.
