# Supplementary material for: A Model of Yeast Cell-Cycle Regulation Based on a Standard Component Modeling Strategy for Protein Regulatory Networks
Source: PLoS One. 2016 May 17;11(5):e0153738. doi: 10.1371/journal.pone.0153738 (PMC4871373; doi:10.1371/journal.pone.0153738)
Supplement: S3 Text — (DOC) [file pone.0153738.s019.doc]

**S3 Text. Equations for the stochastic SCM of the Start transition**

$$\frac{\text{d}V}{\text{d}t}=\mu\cdot V$$

$$\begin{matrix} \frac{\text{d}Cln3}{\text{d}t}=A_{\text{n3}}-B_{\text{n3}}\cdot Cln3+\sqrt{A_{\text{n3}}+B_{\text{n3}}\cdot Cln3}\cdot\frac{\zeta_{1}(t)}{\sqrt{\Delta t}} \\ +Cln3\sqrt{2B_{\text{n3}}\frac{1}{\left\langle m_{\text{n3}}\right\rangle+\left\langle m_{\min,\text{n3}}\right\rangle}\cdot\frac{B_{\text{n3}}}{B_{\text{n3}}+k_{\text{d,mn3}}}}\cdot\frac{\zeta_{2}(t)}{\sqrt{\Delta t}} \end{matrix}$$

$$\begin{matrix} A_{\text{n3}}=k_{\text{s,n3}}\cdot V^{2}\cdot\frac{k_{\text{s,mn3}}}{k_{\text{d,mn3}}} \\ B_{\text{n3}}=k_{\text{d,n3}} \\ \left\langle m_{\text{n3}}\right\rangle=\frac{k_{\text{s,mn3}}}{k_{\text{d,mn3}}} \end{matrix}$$

$$\begin{matrix} \frac{\text{d}ClbS}{\text{d}t}=A_{\text{bS}}-B_{\text{bS}}\cdot ClbS+\sqrt{A_{\text{bS}}+B_{\text{bS}}\cdot ClbS}\cdot\frac{\zeta_{1}(t)}{\sqrt{\Delta t}} \\ +ClbS\sqrt{2B_{\text{bS}}\frac{1}{\left\langle m_{\text{bS}}\right\rangle+\left\langle m_{\min,\text{bS}}\right\rangle}\cdot\frac{B_{\text{bS}}}{B_{\text{bS}}+k_{\text{d,mbS}}}}\cdot\frac{\zeta_{2}(t)}{\sqrt{\Delta t}} \end{matrix}$$

$$\begin{matrix} A_{\text{bS}}=k_{\text{s,bS}}\cdot V\cdot\frac{k_{\text{s,mbS}}}{k_{\text{d,mbS}}}\cdot\left(\frac{SBF}{SBF+\frac{k_{\text{d,g}}\cdot V}{k_{\text{a,g}}}}\right) \\ B_{\text{bS}}=k_{\text{d,bS}} \\ \left\langle m_{\text{bS}}\right\rangle=\frac{k_{\text{s,mbS}}}{k_{\text{d,mbS}}}\cdot\left(\frac{SBF}{SBF+\frac{k_{\text{d,g}}\cdot V}{k_{\text{a,g}}}}\right) \end{matrix}$$

$$\frac{\text{d}Whi5_{\text{A}}}{\text{d}t}=\gamma\cdot(Whi5_{\text{T}}\cdot H(\sigma\cdot W_{\text{i5}})-Whi5_{\text{A}})$$

$$\begin{matrix} \frac{\text{d}Whi5_{\text{T}}}{\text{d}t}=A_{\text{i5}}-B_{\text{i5}}\cdot Whi5_{\text{T}}+\sqrt{A_{\text{i5}}+B_{\text{i5}}\cdot Whi5_{\text{T}}}\cdot\frac{\zeta_{1}(t)}{\sqrt{\Delta t}} \\ +Whi5_{\text{T}}\sqrt{2B_{\text{i5}}\frac{1}{\left\langle m_{\text{i5}}\right\rangle+\left\langle m_{\min,\text{i5}}\right\rangle}\cdot\frac{B_{\text{i5}}}{B_{\text{i5}}+k_{\text{d,mi5}}}}\cdot\frac{\zeta_{2}(t)}{\sqrt{\Delta t}} \end{matrix}$$

$$\begin{matrix} A_{\text{i5}}=k_{\text{s,i5}}\cdot V\cdot\frac{k_{\text{s,mi5}}}{k_{\text{d,mi5}}} \\ B_{\text{i5}}=k_{\text{d,i5}} \\ \left\langle m_{\text{i5}}\right\rangle=\frac{k_{\text{s,mi5}}}{k_{\text{d,mi5}}} \end{matrix}$$

$$\begin{matrix} \frac{\text{d}Hi5}{\text{d}t}=A_{\text{hi5}}-B_{\text{hi5}}\cdot Hi5+\sqrt{A_{\text{hi5}}+B_{\text{hi5}}\cdot Hi5}\cdot\frac{\zeta_{1}(t)}{\sqrt{\Delta t}} \\ +Hi5\sqrt{2B_{\text{hi5}}\frac{1}{\left\langle m_{\text{hi5}}\right\rangle+\left\langle m_{\min,\text{hi5}}\right\rangle}\cdot\frac{B_{\text{hi5}}}{B_{\text{hi5}}+k_{\text{d,mhi5}}}}\cdot\frac{\zeta_{2}(t)}{\sqrt{\Delta t}} \end{matrix}$$

$$\begin{matrix} A_{\text{hi5}}=k_{\text{s,hi5}}\cdot V\cdot\frac{k_{\text{s,mhi5}}}{k_{\text{d,mhi5}}} \\ B_{\text{hi5}}=k_{\text{d,hi5}} \\ \left\langle m_{\text{hi5}}\right\rangle=\frac{k_{\text{s,mhi5}}}{k_{\text{d,mhi5}}} \end{matrix}$$

$$\begin{matrix} \frac{\text{d}SBF_{\text{T}}}{\text{d}t}=k_{\text{s,bf}}\cdot V-k_{\text{d,bf}}\cdot SBF_{\text{T}}+\sqrt{k_{\text{s,bf}}\cdot V+k_{\text{d,bf}}\cdot SBF_{\text{T}}}\cdot\frac{\zeta(t)}{\sqrt{\Delta t}} \\ SBF=\max(0,SBF_{\text{T}}-Whi5_{\text{A}}) \end{matrix}$$

$$H(x)=\frac{1}{\left(1+e^{-x}\right)}$$
