# Supplementary material for: A Model of Yeast Cell-Cycle Regulation Based on a Standard Component Modeling Strategy for Protein Regulatory Networks
Source: PLoS One. 2016 May 17;11(5):e0153738. doi: 10.1371/journal.pone.0153738 (PMC4871373; doi:10.1371/journal.pone.0153738)
Supplement: S4 Text — (DOC) [file pone.0153738.s020.doc]

**S4 Text. Equations for the stochastic SCM of the Start transition with explicit mRNA species**

The variables *m*_n3_, *m*_bS_, *m*_i5_, and *m*_hi5_ refer to numbers of mRNA molecules of Cln3, ClbS, Whi5, and Hi5, respectively.

$$\frac{\text{d}V}{\text{d}t}=\mu\cdot V$$

$$\frac{\text{d}Cln3}{\text{d}t}=k_{\text{s,n3}}\cdot V^{2}\cdot m_{\text{n3}}-k_{\text{d,n3}}\cdot Cln3+\sqrt{k_{\text{s,n3}}\cdot V^{2}\cdot m_{\text{n3}}+k_{\text{d,n3}}\cdot Cln3}\cdot\frac{\zeta(t)}{\sqrt{\Delta t}}$$

$$\frac{\text{d}m_{\text{n3}}}{\text{d}t}=k_{\text{s,mn3}}-k_{\text{d,mn3}}\cdot m_{\text{n3}}+\sqrt{k_{\text{s,mn3}}+k_{\text{d,mn3}}\cdot m_{\text{n3}}}\cdot\frac{\zeta(t)}{\sqrt{\Delta t}}$$

$$\frac{\text{d}ClbS}{\text{d}t}=k_{\text{s,bS}}\cdot V\cdot m_{\text{bS}}-k_{\text{d,bS}}\cdot ClbS+\sqrt{k_{\text{s,bS}}\cdot V\cdot m_{\text{bS}}+k_{\text{d,bS}}\cdot ClbS}\cdot\frac{\zeta(t)}{\sqrt{\Delta t}}$$

$$\frac{\text{d}m_{\text{bS}}}{\text{d}t}=k_{\text{s,mbS}}\cdot\left(\frac{SBF}{SBF+\frac{k_{\text{d,g}}\cdot V}{k_{\text{a,g}}}}\right)-k_{\text{d,mbS}}\cdot m_{\text{bS}}+\sqrt{k_{\text{s,mbS}}\cdot\left(\frac{SBF}{SBF+\frac{k_{\text{d,g}}\cdot V}{k_{\text{a,g}}}}\right)+k_{\text{d,mbS}}\cdot m_{\text{bS}}}\cdot\frac{\zeta(t)}{\sqrt{\Delta t}}$$

$$\frac{\text{d}Whi5_{\text{A}}}{\text{d}t}=\gamma\cdot(Whi5_{\text{T}}\cdot H(\sigma\cdot W_{\text{i5}})-Whi5_{\text{A}})$$

$$\frac{\text{d}Whi5_{\text{T}}}{\text{d}t}=k_{\text{s,i5}}\cdot V\cdot m_{\text{i5}}-k_{\text{d,i5}}\cdot Whi5_{\text{T}}+\sqrt{k_{\text{s,i5}}\cdot V\cdot m_{\text{i5}}+k_{\text{d,i5}}\cdot Whi5_{\text{T}}}\cdot\frac{\zeta(t)}{\sqrt{\Delta t}}$$

$$\frac{\text{d}m_{\text{i5}}}{\text{d}t}=k_{\text{s,mi5}}-k_{\text{d,mi5}}\cdot m_{\text{i5}}+\sqrt{k_{\text{s,mi5}}+k_{\text{d,mi5}}\cdot m_{\text{i5}}}\cdot\frac{\zeta(t)}{\sqrt{\Delta t}}$$

$$\frac{\text{d}Hi5}{\text{d}t}=k_{\text{s,hi5}}\cdot V\cdot m_{\text{hi5}}-k_{\text{d,hi5}}\cdot Hi5+\sqrt{k_{\text{s,hi5}}\cdot V\cdot m_{\text{hi5}}+k_{\text{d,hi5}}\cdot Hi5}\cdot\frac{\zeta(t)}{\sqrt{\Delta t}}$$

$$\frac{\text{d}m_{\text{hi5}}}{\text{d}t}=k_{\text{s,mhi5}}-k_{\text{d,mhi5}}\cdot m_{\text{hi5}}+\sqrt{k_{\text{s,mhi5}}+k_{\text{d,mhi5}}\cdot m_{\text{hi5}}}\cdot\frac{\zeta(t)}{\sqrt{\Delta t}}$$

$$SBF=\max(0,SBF_{\text{T}}-Whi5_{\text{A}})$$

$$\frac{\text{d}SBF_{\text{T}}}{\text{d}t}=k_{\text{s,bf}}\cdot V-k_{\text{d,bf}}\cdot SBF_{\text{T}}+\sqrt{k_{\text{s,bf}}\cdot V+k_{\text{d,bf}}\cdot SBF_{\text{T}}}\cdot\frac{\zeta(t)}{\sqrt{\Delta t}}$$
