# Supplementary material for: A Model of Yeast Cell-Cycle Regulation Based on a Standard Component Modeling Strategy for Protein Regulatory Networks
Source: PLoS One. 2016 May 17;11(5):e0153738. doi: 10.1371/journal.pone.0153738 (PMC4871373; doi:10.1371/journal.pone.0153738)
Supplement: S5 Text — (DOC) [file pone.0153738.s021.doc]

**S5 Text. Mutant simulations and discussion of problems**

We simulated mutant strains of budding yeast using the Eqs. 21–69 in Table 4 with initial conditions from Table 3 and wild-type parameter values (Table 5), except for certain parameter values that were adjusted to represent the effects of the specific alleles in each mutant strain. For example, we simulated the $cln2\Delta$ allele (deletion of the *CLN2* gene) by setting synthesis rates of Cln2 equal to 0. The list of all mutant strains simulated by our deterministic model is given in S3 Table. The adjusted parameters for each mutant allele are given in S4 Table. Cells are considered to be arrested in a particular phase of the cell cycle according to the rules in S5 Table. The simulated mutant strains that do not agree with experimental observations are marked with asterisks in S3 Table and collected for closer examination in S6 Table.

*cln3*Δ *bck2*Δ *whi5*Δ

In our model, the SBF component represents both SBF and MBF (transcription factors for Cln1/Cln2 and Clb5/Clb6) and inactivation of Whi5 by Cln3 and Bck2 is the only requirement for the Start transition. Therefore, without Whi5, the Start transition occurs without the need of Cln3 and Bck2, and these mutant cells (in our simulation) enter G_1_ earlier than wild-type cells and at a smaller size. In reality, Whi5 does not interact with MBF, and the activation of MBF at Start depends on processes other than the inactivation of Whi5. If, as is likely, Cln3 and Bck2 participate in the activation of MBF, then the Start transition in *cln3*Δ *bck2*Δ *whi5*Δ will be delayed, and this will explain the larger than WT size for the mutant.

*GAL-CLB5-db*Δ

In our simulation, *GAL-CLB5-db*Δ mutant cells accumulate a lot of Clb5, which suppresses the activity of Cdh1 during mitotic exit, and the cells arrest in telophase as a result. If we reduce the inhibition of Cdh1 by Clb5, then the mutant cells will exit from mitosis and arrest in the next cycle because they fail to relicense origins of replication. However, the parameter changes that fix the phenotype of *GAL-CLB5-db*Δ cells cause problems for the simulations of other mutant strains: notably, *cdc20*Δ *pds1*Δ and *cdc20* *CLB5db*Δ *pds1*Δ, both of which would be viable instead of arresting in telophase if the inhibition of Cdh1 by Clb5 were too low.

*CLB1* *clb2*Δ *cdh1*Δ and *CLB1* *clb2*Δ *pds1*Δ

As in the original model , these two mutant strains are not in agreement with observed phenotypes. In we suggested that during mitotic exit Clb2 might have roles that are not included in our model and are not carried out by Clb1.

*cdc15*Δ *net1*-*ts cdh1*Δ

The *cdc15*Δ mutant is inviable since Cdc14 is not released during mitotic exit. The arrest is rescued by the partial release of Cdc14 in *cdc15*Δ *net1*-*ts* cells. However, when *CDH1* is deleted from this strain (*cdc15*Δ *net1*-*ts cdh1*Δ), our model cannot account for the viability of the triple mutant. Without Cdh1, cells must rely on the activity of CKI for mitotic exit. In our model, the partial release of Cdc14 in the *cdc15*Δ *net1*-*ts cdh1*Δ mutant is not enough to enable CKI-dependent mitotic exit.

*net1-ts* in nocodazole

According to experimental observations, *net1-ts cdc20*Δ cells arrest in metaphase but *net1-ts* cells are not arrested in nocodazole. We cannot account for both observations, because nocodazole arrest prevents the activation of Cdc20; so *net1-ts* cells in nocodazole should have the same phenotype as *net1-ts cdc20*Δ cells.

*bub2*Δ in nocodazole and *bub2*Δ *pds1*Δ in nocodazole

In both of these strains, although the BUB2 pathway is defective (*ω*_i,tem1,px_ = 0), the MAD2 pathway is still functional, so Cdc20 is expected to be inactive in these cells in nocodazole. Therefore, for both strains in nocodazole, although Tem1 should be active, Cdc15 should be inactive because Clb2-kinase activity remains high (Cdc20 is inactive) and Cdc14 activity remains low. (In the *bub2*Δ *pds1*Δ strain, there is a transient release of Cdc14 by the FEAR pathway but not enough to activate Cdc15 in the face of high Clb2-kinase.) Hence, for both mutant strains in nocodazole, the MEN cannot be activated, and the cells cannot exit from mitosis. This is the case in our simulations; nonetheless, both mutant strains are observed to exit from mitosis in nocodazole. In we suggested that the BUB2 pathway may cross-talk with the MAD2 pathway; perhaps a signal from the BUB2 pathway promotes the activity of Mad2. In that case, without Bub2, Mad2 would have less activity, allowing the mutant cells to exit from mitosis even though mitotic spindles are not properly assembled.

**References**

1. Chen KC, Calzone L, Csikasz-Nagy A, Cross FR, Novak B, Tyson JJ. Integrative analysis of cell cycle control in budding yeast. Mol Biol Cell. 2004;15(8):3841-62.
