# Supplementary material for: A Model of Yeast Cell-Cycle Regulation Based on a Standard Component Modeling Strategy for Protein Regulatory Networks
Source: PLoS One. 2016 May 17;11(5):e0153738. doi: 10.1371/journal.pone.0153738 (PMC4871373; doi:10.1371/journal.pone.0153738)
Supplement: S6 Text — (DOC) [file pone.0153738.s022.doc]

**S6 Text. Model conversion**

In this section, we explain how to convert the normalized concentration units used in the deterministic SCM of the full system of the budding yeast cell cycle into numbers of molecules using the conversion process described in . Briefly, the first step is to convert the normalized concentrations into real concentrations

| $[\text{S}]=c_{\text{S}}\cdot[\text{S}]_{\text{n}},$ | (A7) |
| --- | --- |

where [S] is the concentration of protein S in nM, [S]_n_ is the normalized concentration used in the deterministic model, and *c*_S_ is the characteristic concentration (the scaling factor) for protein S. The characteristic concentrations for all variables are listed in Table 3. These values are obtained from with minor changes to match the numbers of molecules calculated from our model with the numbers reported in the literature .

With this substitution the ODE for a normalized (dimensionless) variable

| $\frac{\text{d}[\text{S}]_{\text{n}}}{\text{d}t}=k_{\text{s,S}}+k_{\text{s,S,B}}\cdot[\text{B}]_{\text{n}}-k_{\text{d,S}}\cdot[\text{S}]_{\text{n}}-k_{\text{d,S,C}}\cdot[\text{C}]_{\text{n}}\cdot[\text{S}]_{\text{n}}$ | (A8) |
| --- | --- |

is converted to an ODE for protein concentration in nM of the form

| $\frac{\text{d}[\text{S}]}{\text{d}t}=c_{\text{S}}\cdot k_{\text{s,S}}+\frac{c_{\text{S}}}{c_{\text{B}}}\cdot k_{\text{s,S,B}}\cdot[\text{B}]-k_{\text{d,S}}\cdot[\text{S}]-\frac{k_{\text{d,S,C}}}{c_{\text{C}}}\cdot[\text{C}]\cdot[\text{S}].$ | (A9) |
| --- | --- |

*k*_s,S_, *k*_s,S,B_, *k*_d,S_, and *k*_d,S,C_ are the original parameter constants from the (normalized) deterministic model. They represent the basal synthesis rate, the synthesis rate regulated by the transcription factor B, the basal degradation rate, and the degradation rate regulated by protein C, respectively. *c*_S_, *c*_B_, and *c*_C_ are the characteristic concentrations of proteins S, B, and C, respectively.

Next we convert the real concentration of protein S to numbers of molecules (*S*)

|  | (A10) |
| --- | --- |

Here, [S] is concentration in nM, *V*_n_ is (normalized) cell volume, *c*_vol_ is average cell size at birth of wild-type daughter cells (28 fL), and *N*_A_ = Avogadro’s number = 6.02×10^23^ molecules/mol. With this change of variables, Eq. A9 becomes

|  | (A11) |
| --- | --- |

In this equation, the term +*µ∙S* comes from the exponential increase of *V*_n_ in the model (*µ* = specific growth rate ≈ 0.007 min^−1^), and it should be cancelled by a “dilution” term −*µ∙S*, which should be present in Eqs. A8 and A9. In Chen’s model and in most deterministic models of the cell cycle, these dilution terms are ignored because they are usually negligible compared to the rates of proteolytic turnover of unstable proteins in the model. In order that our “molecule rate equations” (Eq. A11) will be consistent with our “normalized rate equations” (Eq. A8), we must either retain the +*µ∙S* terms in molecule rate equations or add −*µ∙*[S]_n_ terms in normalized rate equations. Since we are already committed to normalized rate equations as they are usually written without −*µ∙*[S]_n_ terms, we retain the +*µ∙S* terms in molecule rate equations.

In like manner, we transform the ODEs for class-2 variables in normalized variables [Z]_n_

| $\begin{matrix} \frac{\text{d}[\text{Z}]_{\text{n}}}{\text{d}t}=\gamma\cdot\left(\frac{[\text{Z}_{\text{T}}]_{\text{n}}}{1+e^{-\sigma\cdot W_{\text{Z}}}}-[\text{Z}]_{\text{n}}\right), \\ \text{where }W_{\text{Z}}=\omega_{0}+\omega_{\text{B}}\cdot[\text{B}]_{\text{n}}-\omega_{\text{C}}\cdot[\text{C}]_{\text{n}} \end{matrix}$ | (A12) |
| --- | --- |

to real concentrations [Z] (in nM)

|  | (A13) |
| --- | --- |

to numbers of molecules per cell *Z*

|  | (A14) |
| --- | --- |

where *ω*_0_, *ω*_B_, and *ω*_C_ are original parameter values from the (normalized) deterministic model.

Class-3 variables are calculated as functions of the other two classes by equations of the form

| $Z=\max(0,\text{ }Z_{\text{T}}-I_{\text{T}}),$ | (A15) |
| --- | --- |

where *Z* is the number of free Z molecules, *Z*_T_ represents the total number of Z molecules, and *I*_T_ is the total number of the stoichiometric inhibitor molecules. *Z*_T_ and *I*_T_ can be variables of either class 1 or 2.

**References**

1. Wang P, Randhawa R, Shaffer CA, Cao Y, Baumann WT. Converting macromolecular regulatory models from deterministic to stochastic formulation. Proceedings of the 2008 Spring simulation multiconference; Ottawa, Canada. 1400608: Society for Computer Simulation International; 2008. p. 385-92.

2. Ball DA, Ahn TH, Wang PY, Chen KC, Cao Y, Tyson JJ, et al. Stochastic exit from mitosis in budding yeast: model predictions and experimental observations. Cell Cycle. 2011;10(6):999-1009.

3. Cross FR, Archambault V, Miller M, Klovstad M. Testing a mathematical model of the yeast cell cycle. Mol Biol Cell. 2002;13(1):52-70.

4. Ghaemmaghami S, Huh WK, Bower K, Howson RW, Belle A, Dephoure N, et al. Global analysis of protein expression in yeast. Nature. 2003;425(6959):737-41.

5. Chen KC, Calzone L, Csikasz-Nagy A, Cross FR, Novak B, Tyson JJ. Integrative analysis of cell cycle control in budding yeast. Mol Biol Cell. 2004;15(8):3841-62.
