# Supplementary material for: A Model of Yeast Cell-Cycle Regulation Based on a Standard Component Modeling Strategy for Protein Regulatory Networks
Source: PLoS One. 2016 May 17;11(5):e0153738. doi: 10.1371/journal.pone.0153738 (PMC4871373; doi:10.1371/journal.pone.0153738)
Supplement: S7 Text — (DOC) [file pone.0153738.s023.doc]

**S7 Text. The mRNA-inherited noise term of the full budding yeast cell cycle model**

From S2 Text and Eq. 19 of the main text, we derived the mRNA-inherited noise term as

where $X_{i}$ represents the number of molecules of the protein $\text{X}_{i}$. $A_{i}$ and $B_{i}$ are the production and degradation rates of protein $\text{X}_{i}$. $\left\langle m_{i}\right\rangle$ is the average number of mRNA molecules encoding protein X*_i_* at steady state, and $k_{\text{dm}}$ is the rate constant for mRNA degradation.$\left\langle m_{\min}\right\rangle$, the minimum number of mRNA molecules always present in the cell, is a parameter introduced to limit the magnitude of mRNA-inherited noise when $\left\langle m_{i}\right\rangle$ is small. $\zeta(t)$ is a random variable chosen from a normal distribution N(0,1) with mean = 0 and standard deviation = 1, and $\Delta t$ is the step size of the numerical integration.

Since our full model of the budding yeast cell cycle does not include any information about mRNA species, we set $\left\langle m_{i}\right\rangle=A_{i}/(k_{\text{tr}}\cdot V_{\text{fL}})$, where $A_{i}$ is the protein production rate (the number of protein molecules produced per min), $k_{\text{tr}}$ is the protein translation rate (the number of protein molecules produced per mRNA molecule per fL per min), and $V_{\text{fL}}$ is cell volume in fL. (We assume that the number of ribosome molecules increases as the cell grows.) This yields Eq. 70 in the main text:

where we treat $k_{\text{tr}}$, $\left\langle m_{\text{min}}\right\rangle$, and $k_{\text{dm}}$ as constant parameters.
