# Supplementary material for: A Model of Yeast Cell-Cycle Regulation Based on a Standard Component Modeling Strategy for Protein Regulatory Networks
Source: PLoS One. 2016 May 17;11(5):e0153738. doi: 10.1371/journal.pone.0153738 (PMC4871373; doi:10.1371/journal.pone.0153738)
Supplement: S8 Text — (DOC) [file pone.0153738.s024.doc]

**S8 Text. The effects of the parameters <*m*_min_> on the model**

In our models, when <*m*_i_> and *A_i_* in our Start and full models, respectively, are too small, the Langevin approximation accounting for the effect of mRNA noise breaks down. Especially for the case of ClbS in the SCM of the Start transition, when <*m*_bS_> is 0 (no transcription factor; *SBF*=0), we encounter the problem of a number getting divided by 0. We, therefore, assign the value of <*m*_min,bS_> to be 1 molecule. In figures below we compare the stochastic simulation results when <*m*_min,bS_> is set to be 1, 0.01, and 5. Overall, the different values of <*m*_min,bS_> do not have significant effects on the statistics of the simulation results. However, setting <*m*_min,bS_> = 0.01 caused the G_1_ transition to occur before the Start transition (*T*_G1_<*T*_1_) in some of the simulation runs (7 from 100 runs). The incorrect order is not observed in the MultiP model and the SCM with <*m*_min,bS_> = 1 or 5.

**Mean values of *T*_1_ and *T*_G1_ from stochastic simulations of the Start SCM compared between models with different values of <*m*_min,bS_>.**

**Coefficient of variation values of *T*_1_ and *T*_G1_ from stochastic simulations of the Start SCM compared between models with different values of <*m*_min,bS_>.**

In our full model of the budding yeast cell cycle, we check if wild-type cells always execute all cell cycle events in an orderly progression: cell division, origins of replication relicensing, bud emergence/DNA synthesis initiation, spindle assembly completion, and another cell division. Since we did not constrain the sequence of these events in our model, noise could drive the system into an incorrect order of events. We found that high fluctuations in numbers of molecules of Cln2, Clb2, Clb5, CKI, and Pds1 due to our mRNA-inherited noise (e.g., by setting <*m*_min_,*_i_*> of these components = 0 or 1) result in some cells that execute the wrong sequence of the normal cell cycle. This is because cell cycle events in our model, such as relicensing of origins of replication, bud formation, and spindle assembly, are strictly controlled by these components. We can resolve this problem by setting <*m*_min_,*_i_*> of these components = 5 molecules to suppress their fluctuations. In figures below, we compare the statistical properties of the cell cycle when <*m*_min_,*_i_*> of these components is set at 5, 1, and 0.01. Setting <*m*_min_,*_i_*> of the components = 1 or 0.01 obviously increased variations of *T*_1_, *T*_2_, and *T*_G1_ in mother cells but it also caused some simulated cells to not execute cell cycle events in an orderly progression.

**Statistical properties of cell cycle progression of mother cells from the SCM of the full budding yeast cell cycle system compared between models with different values of <*m*_min_,*_i_*> for *CLN2*, *CLB2*, *CLB5*, *CKI*, and *PDS1*. Asterisks indicate unreported data.**

**Statistical properties of cell cycle progression of daughter cells from the SCM of the full budding yeast cell cycle system compared between models with different values of <*m*_min_,*_i_*> for *CLN2*, *CLB2*, *CLB5*, *CKI*, and *PDS1*. Asterisks indicate unreported data.**
