# Supplementary material for: A Model of Yeast Cell-Cycle Regulation Based on a Standard Component Modeling Strategy for Protein Regulatory Networks
Source: PLoS One. 2016 May 17;11(5):e0153738. doi: 10.1371/journal.pone.0153738 (PMC4871373; doi:10.1371/journal.pone.0153738)
Supplement: S9 Text — (DOC) [file pone.0153738.s025.doc]

**S9 Text. Simulation methods**

We used the first-order explicit Euler method with step size (∆*t*) = 0.01 min as the numerical method for both deterministic and stochastic simulations of the SCMs (Start and full cell cycle models) and for deterministic simulations of the MultiP model. (In S10 Text we discuss the effects of the integration step size on the models.) Stochastic simulations of the MultiP model employed Gillespie's SSA. The bifurcation diagrams in Fig 2 of the main text and S2 Fig were calculated by XPP-AUTO, available from <http://www.math.pitt.edu/~bard/xpp/xpp.html>.

All simulations of the full model were based on Eqs. 21–69 of Table 4, with parameter values for wild-type cells given in Table 5, and initial conditions specified in Table 3. Parameter values for mutant strains were derived from Table 5 by making relevant changes. For example, to simulate *cln2*∆ (*CLN2* deletion) cells, we set the synthesis rate constants of Cln2 (*k*_s,cln2_ and *k*_s,cln2,bf_) equal to 0. The parameter values used for each mutant strain are listed in S4 Table.

For stochastic simulations of wild-type cells, we started each simulation at the initial conditions in Table 3 and simulated for 2000 min, following the lineage of mother cells in 40 simulations and the lineage of daughter cells in another 40 simulations. At the end of 2000 min, we recorded the state of the last cell, obtaining 80 different cell states that are representative of a sample of cells drawn from an asynchronous population of proliferating yeast cells. For each of these 80 states, we simulated for another 600 min, following both mother and daughter cell at each division. For each complete cell cycle (sample size ~5000), we recorded cycle time, *T*_1_, *T*_2_, and *T*_b_ durations, and size at birth, and we used this data to calculate statistical properties of the cell cycle (Fig 10 of the main text).

A similar procedure was used to simulate *CLB2*-*db*∆ *clb5*∆ *GAL-SIC1* cells. We created 80 initial cell states by simulating mutant cells, growing in galactose medium with Sic1 overexpression, for 2000 min, to get a representative sample of mutant cells kept alive (by Sic1 overexpression) on galactose medium. We then followed these 80 initial cells for another 600 min in three different conditions: (1) in galactose, (2) in raffinose, and (3) in glucose. We recorded the cell cycle time from each complete cell cycle. For cycles that were not completed by the end of the simulation, we recorded the time from birth as a “minimal” cycle time. Only data from complete cycles was used to calculate statistical properties (Table 7). On the other hand, both complete cycle times and minimal cycle times were used to calculate the conditional probability functions in Fig 12, bottom panel. The plots were calculated by the Kaplan-Meier estimator using the ecdf function in MATLAB. In these plots, minimal cycle times were treated as censored data.

In deterministic simulations of the *CLB2*-*db*∆ *clb5*∆ strain, the degradation rate of Clb2 regulated by Cdh1 (*k*_d,clb2,h1_) is reduced to 8.5% due to the remaining KEN box . However, in our stochastic model, we can fit the plot in Fig 12, bottom panel, better if we reduce the Cdh1-dependent Clb2 degradation rate to 7%. In deterministic simulations, this new value of *k*_d,clb2,h1_ predicts that *CLB2*-*db*∆ *clb5*∆ cells are inviable in raffinose, but the mutant strain shows ~75% viability in stochastic simulations. Note that the changed value of *k*_d,clb2,h1_ affects only mutant strains in the *CLB2-db*∆ group.

In our deterministic simulations of cells growing in galactose and raffinose media (*mdt* = 150 min), daughter and mother cells are given 39% and 61%, respectively, of the progenitor cell size at division, because it is well known that daughter cells growing in galactose are smaller than daughter cells growing in glucose. However, to compare our stochastic simulations of *CLB2*-*db*∆ *clb5*∆ cells to the results in , we adopted the values used in for our stochastic simulations (daughter cells receive 48% and mother cells receive 52% of the size at division in galactose and raffinose).

**References**

1. Pfleger CM, Kirschner MW. The KEN box: an APC recognition signal distinct from the D box targeted by Cdh1. Genes Dev. 2000;14(6):655-65.

2. Ball DA, Ahn TH, Wang PY, Chen KC, Cao Y, Tyson JJ, et al. Stochastic exit from mitosis in budding yeast: model predictions and experimental observations. Cell Cycle. 2011;10(6):999-1009.
