# Supplementary material for: A Model of Yeast Cell-Cycle Regulation Based on a Standard Component Modeling Strategy for Protein Regulatory Networks
Source: PLoS One. 2016 May 17;11(5):e0153738. doi: 10.1371/journal.pone.0153738 (PMC4871373; doi:10.1371/journal.pone.0153738)
Supplement: S10 Text — (DOC) [file pone.0153738.s026.doc]

**S10 Text. The effects of the integration step size (Δ*t*) on the model**

The integration step size used in our models of the Start transition and the full cell cycle system is 0.01 min. In figures below we compare the simulation results from the Start and the full models using Δ*t* =0.01, 0.001, and 0.5. Overall, the changes in Δ*t* around the default value (0.01) do not show significant effects on the simulations.

**Mean values of *T*_1_ and *T*_G1_ from stochastic simulations of the Start models. The MultiP model was simulated using Gillespie’s stochastic simulation algorithm. The SCM was simulated by an explicit Euler method with different integration step sizes (Δ*t*).**

**Coefficient of variation values of *T*_1_ and *T*_G1_ from stochastic simulations of the Start models. The MultiP model was simulated using Gillespie’s stochastic simulation algorithm. The SCM was simulated by an explicit Euler method with different integration step sizes (Δ*t*).**

**Statistical properties of cell cycle progression of mother cells from the SCM of the full budding yeast cell cycle system, for Euler integration of the Langevin equations with different integration step sizes (Δ*t*). Asterisks indicate unreported data.**

**Statistical properties of cell cycle progression of daughter cells from the SCM of the full budding yeast cell cycle system, for Euler integration of the Langevin equations with different integration step sizes (Δ*t*). Asterisks indicate unreported data.**
